# Design and synthesis of multivalent α-1,2-trimannose-linked bioerodible microparticles for applications in immune response studies of *Leishmania major* infection

**DOI:** 10.3762/bjoc.15.58

**Published:** 2019-03-11

**Authors:** Chelsea L Rintelmann, Tara Grinnage-Pulley, Kathleen Ross, Daniel E K Kabotso, Angela Toepp, Anne Cowell, Christine Petersen, Balaji Narasimhan, Nicola Pohl

**Affiliations:** 1 Department of Chemistry, Indiana University Bloomington, 800 E. Kirkwood Ave., Bloomington, Indiana 47405-7102, USA; 2 Department of Epidemiology, College of Public Health, University of Iowa, 105 River Street, S444 CPHB, Iowa City, Iowa 52242, USA; 3 Center for Emerging Infectious Diseases, University of Iowa Research Park, 2500 Crosspark Road, MTF B166 Coralville, Iowa 52241, USA; 4 Nanovaccine Institute, Iowa State University, 2114 Sweeney Hall, Ames, Iowa 50011-2230, USA; 5 Department of Chemical and Biological Engineering, Iowa State University, 618 Bissell Road, Ames, Iowa 50011-2230, USA

**Keywords:** adjuvant, carbohydrates, *L. major*, microparticle, PAMP

## Abstract

Leishmaniasis, a neglected tropical disease, currently infects approximately 12 million people worldwide with 1 to 2 million new cases each year in predominately underdeveloped countries. The treatment of the disease is severely underdeveloped due to the ability of the *Leishmania* pathogen to evade and abate immune responses. In an effort to develop anti-leishmaniasis vaccines and adjuvants, novel carbohydrate-based probes were made to study the mechanisms of immune modulation. In this study, a new bioerodible polyanhydride microparticle was designed and conjugated with a glycodendrimer molecular probe. This molecular probe incorporates a pathogen-like multivalent display of α-1,2-trimannose, for which a more efficient synthesis was designed, with a tethered fluorophore. Further attachment of the glycodendrimer to a biocompatible, surface eroding microparticle allows for targeted uptake and internalization of the pathogen-associated oligosaccharide by phagocytic immune cells. The α-1,2-trimannose-linked bioerodible microparticles were found to be safe after administration into the footpad of mice and demonstrated a similar response to α-1,2-trimannose-coated latex beads during *L. major* footpad infection. Furthermore, the bioerodible microparticles allowed for investigation of the role of pathogen-associated oligosaccharides for recognition by pathogen-recognition receptors during *L. major-*induced leishmaniasis.

## Introduction

Recognition of parasite cell surface molecules by host immune cells initiates the first step in the immune response [1–2]. The host’s immune system recognizes parasite surface glycoconjugates, or pathogen-associated molecular patterns (PAMPs), to build an immune response against the parasite and impede disease progression. Antigen presenting cells (APCs) recognize these PAMPs as pathogenic as compared to host glycans making these moieties feasible targets for both parasite detection and vaccine development [3].

*Leishmania*, an obligate intracellular parasite with varying surface glycoconjugates, infects an estimated 12 million people worldwide and has 350 million people at risk in endemic areas (predominately in underdeveloped countries). The World Health Organization estimates that this neglected tropical disease causes between 1–2 million new cases annually [4]. Leishmaniasis manifests into three different forms, cutaneous, mucosal, or visceral, depending on parasite species, host immune system and location of infection [5]. Cutaneous, the most common form, causes severe nodular and ulcerative skin lesions, while mucocutaneous destroys mucus membranes, and the most deadly form, visceral leishmaniasis, often results in organ failure [6]. *L. major*, the parasite used in our in vivo model, causes the cutaneous disease. In each pathogenesis, the parasite infects and propagates in immune cells, the very cells responsible for host protection. Through a range of mechanisms not entirely understood, *Leishmania* subverts activation of antimicrobial (nitric oxide) and cytokine inducible functions that initiate effective immunity [5]. There are no approved anti-leishmanial vaccines and the current therapeutics are toxic, expensive and prone to induce resistance, such as, amphotericin B (AmB), paromomycin and antimony-based chemotherapeutics [7–9]. With growing resistance to antileishmanial drugs and poor patient compliance, the development of safe, efficacious and inexpensive alternatives to current therapeutics is needed. Understanding the mechanisms by which *Leishmania* regulates host immune responses are crucial for the development of vaccines, which may induce adaptive immunity that may prove beneficial for combating other intracellular pathogens, such as *Mycobacterium tuberculosis* [10–11].

**Figure 1:**
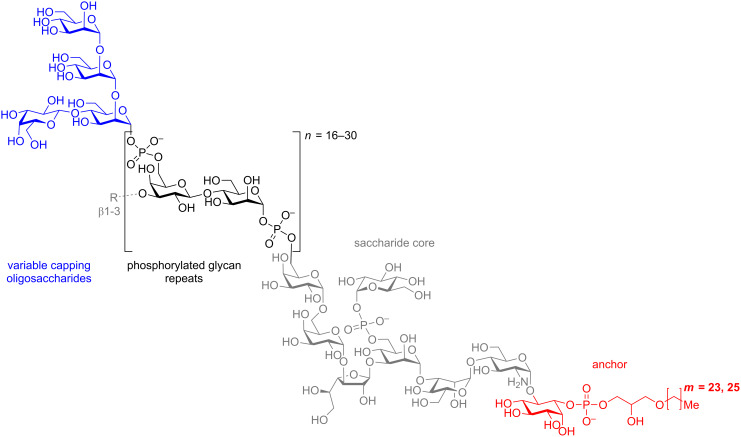
*Leishmania* lipophosphoglycan (LPG), with Gal-β-(1→4)-[Man-α-(1→2)-Man-α-(1→2)-Man] variant capping structure highlighted in blue and R = H or species dependent oligosaccharide [25].

Carbohydrate-based probes provide one method to investigate parasitic mechanisms of immune suppression and evasion. The cell surface glycoconjugates on *Leishmania* have been implicated in the ability of the parasite to infect host cells, then evade and suppress host immune responses [5,12–15]. The most prominent of these cell surface glycoconjugates, lipophosphoglycan (LPG), consists of a glycosylphosphatidylinositol (GPI) anchor and variable sugar capping structures (Figure 1) [15–20]. The capping sugar tropisms vary with *Leishmania* species (>20 species) and life stage of the pathogen, making it challenging to define how the capping sugars interact and modulate host immune responses [21–24]. Since these cell-surface glycans are structurally diverse and difficult to isolate in appreciable quantities, synthetic pathogen-associated carbohydrate probes are necessary to understand the LPG structure-to-function relationship. These synthetic oligosaccharides also provide a structurally homogeneous standard to study how these glycans interact with the host immune system in an effort to develop effective anti-*Leishmania* vaccines.

Previously, discrete structural components of LPG have been synthesized [26–38] and their involvement in modulating an immune response of these reduced motifs has been investigated [34–36,39]. The Pohl and Petersen groups have previously investigated how truncated oligosaccharide capping structures of LPG interact with macrophage pattern recognition receptors (PRRs) to modulate an innate immune response to *L. major*-induced leishmaniasis in vitro and in vivo [11,40–42]. These studies showed that a neutral α-1,2-trimannose capping structure alone is sufficient to modulate an innate immune response through toll-like receptor 2 (TLR-2) and mannose receptor (MR)-dependent pathways by increasing the production of IL-12, a key cytokine involved in recruiting a T cell-mediated adaptive immune response [11,40,42]. Furthermore, our acid-labile probe was found to increase T cell production, suggesting α-1,2-trimannose antigen presentation at the major histocompatibility complex II (MHC II) [41].

**Figure 2:**
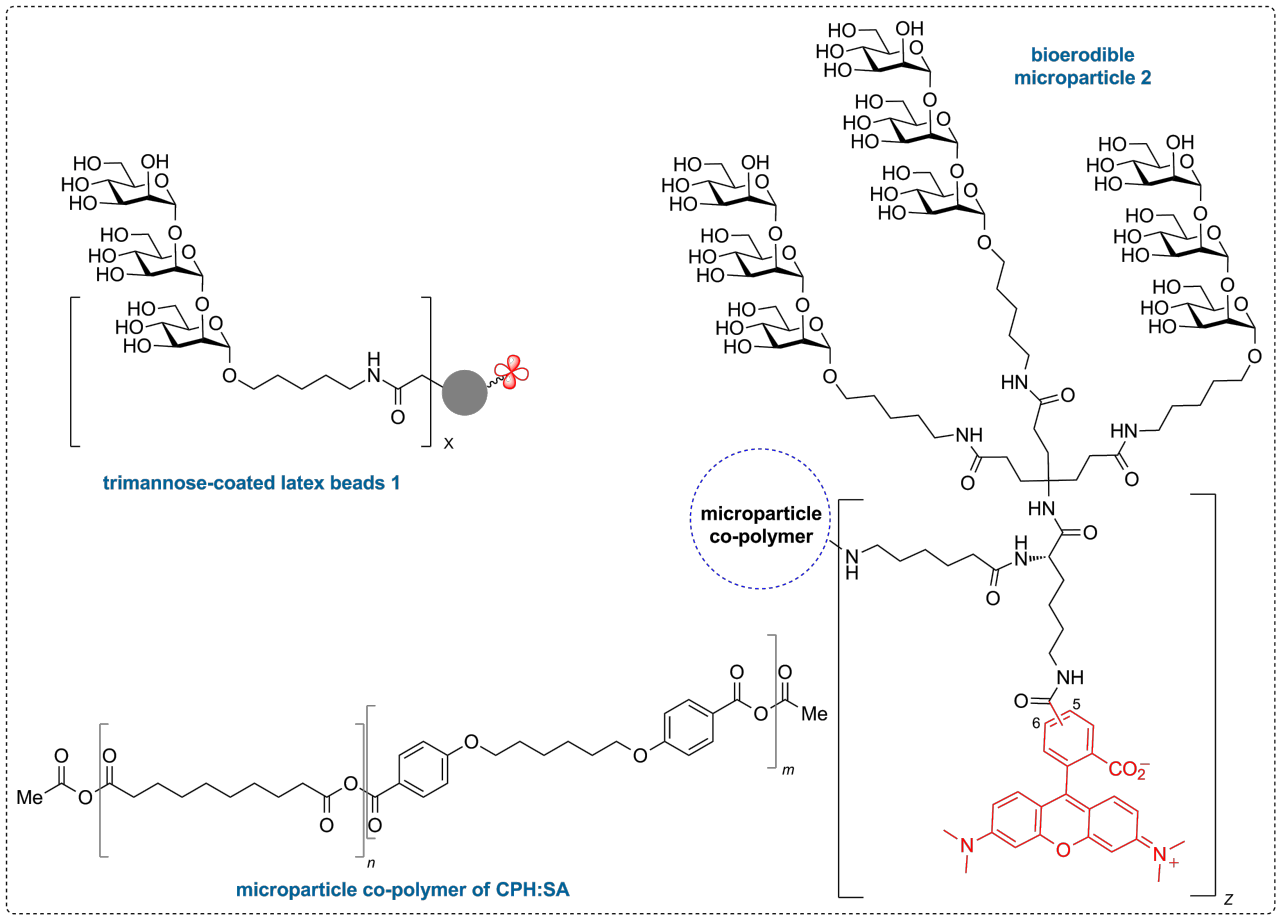
Pathogen-associated bioerodible microparticles **2** and trimannose-coated latex beads **1**.

Based on our previous studies [11,40–42], we sought to design and synthesize a new *Leishmania*-associated molecular probe that could allow intracellular monitoring and validation of T cell antigen presentation. This new probe would also be linked to the surface of a polyanhydride copolymer microparticle based on 1,6-bis(*p*-carboxyphenoxy)hexane (CPH) and sebacic anhydride (SA) to allow for enhanced internalization and uptake of the immunogenic glycoconjugate through recognition by phagocytic cells [43–45]. Here we discuss the design and synthesis of this new bioerodible microparticle **2** compared to our previously designed [11] trimannose-coated latex bead construct **1** (Figure 2) and evaluate the biocompatibility of the microparticle in spared dose administration to *L. major* infected mice (Figure 4).

## Results and Discussion

**Figure 3:**
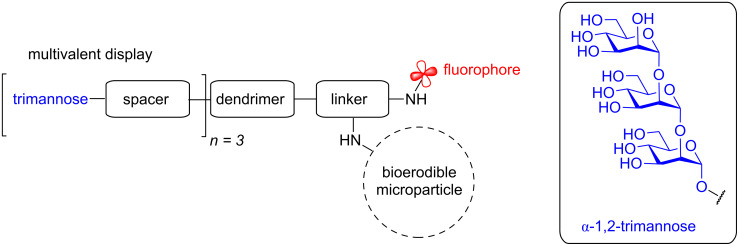
General design of the bioerodible microparticles.

To further probe the biological mechanisms responsible for *L. major* immune regulation, a new adjuvant-like pathogen-associated molecular probe was designed to promote pathogen recognition receptor (PRR)-mediated internalization and slow erosion in the acidic phagolysosome of macrophages. This bioerodible microparticle **2** design evolved from earlier synthetic probes with improved translatability for therapeutic development [11,41–42]. To mimic the *Leishmania* pathogen, the size of our probe needed to be approximately the same size as the pathogen (1 μm in diameter) and provide a multivalent display of the synthetic α-1,2-trimannose similar to the *Leishmania* promastigote cell-wall glycans. Furthermore, a tethered fluorophore would allow for immunofluorescence assays, intracellular trafficking, and validation of T cell-mediated antigen recognition. To allow for both surface functionalization of this glycan to the bioerodible microparticles and conjugation of the fluorophore, two amine handles would be necessary (Figure 3). The design would also benefit from the development of a more efficient synthesis of the mannose oligomer sidechain.

From the general design elements in Figure 3, we proposed the following synthetic motifs. For PRR binding, a dendrimeric scaffold provides a multivalent display of the α-1,2-trimannose glycoconjugate, similar to *Leishmania* LPG and additionally may promote glycodendrimer antigen presentation when hydrolyzed in the acidic phagolysosome [39,41,46]. To improve the overall synthetic sequence from our previous syntheses [11,41–42], an amino alcohol spacer was utilized to connect the trimannose reducing end to the dendrimer by means of amide bond coupling. Furthermore, the amino alcohol spacer provides a handle for the incorporation of a fluorous-tag for ease of purification by fluorous solid phase-extraction (FSPE). The use of the fluorous tag enables solution-phase automated synthesis of α-1,2-trimannose in the future as shown previously with a related spacer and fluorous tag [47]. Incorporation of the commercially available rhodamine dye, TAMRA (555_ex_/580_em_), to the dendrimer would allow for standard immunofluorescence assays, to track the microparticle following cellular uptake and intracellular processing. TAMRA’s high photostability, low pH sensitivity and ease of incorporation through amide bond coupling, facilitates synthetic manipulation and biocompatibility [48–49].

In an effort to make a direct comparison to our previous biological studies, trimannose-coated latex beads also were synthesized using 1 μm FluoSpheres^©^ carboxylate-modified latex beads labeled with a red fluorophore (emission maximum 580 nm). The latex-bead synthesis was modified slightly from previous synthesis for improved ease of synthesis of trimannose and comparability to this study’s bioerodible microparticles by coupling the reducing end of trimannose with the aforementioned amino alcohol spacer [11,41]. Biological analysis of the trimannose-coated latex beads was described previously [42] and used to compare the new construct.

### Synthesis of α-1,2-trimannose

**Scheme 1:**
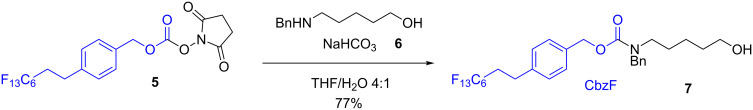
Synthesis of fluorous CbzF-protected aminopentanol spacer **7**.

The synthesis of α-1,2-trimannose, required for both the glycodendrimer **2** and latex beads **1**, was achieved through iterative glycosylation with known trichloroacetimidate mannosyl donors **3** and **4** [47,50] and the use of a fluorous tag containing an amino alcohol spacer rather than an alkene that would require late-stage modifications. The aminopentanol spacer **6** used at the reducing end to conjugate to the dendrimer was first protected with fluorous CbzF-NHS **5** to allow for purification by FSPE of the mannosyl intermediates and to facilitate future automated syntheses to produce α-1,2-trimannose in appreciable quantities similar to previous efforts (Scheme 1) [11,47,51].

**Scheme 2:**
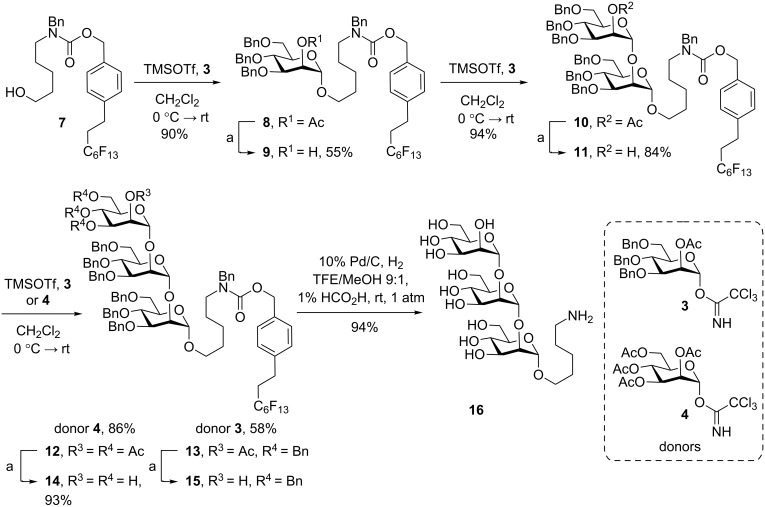
Iterative synthesis of α-1,2-trimannose **16**. Reagents and conditions: a) 1 M NaOH, MeOH or NaOMe.

To synthesize the oligosaccharide, activation of TCA donor **3** with TMSOTf and glycosylation of the CbzF-protected aminopentanol **7** afforded the monosaccharide **8** (Scheme 2). The 2-*O*-position was then deacylated, followed by iterative glycosylation/deacetylation with donor **3** to provide dimannoside acceptor **11**. TCA-mannose donor **3** was initially used to cap the dimannoside **11**. However, hydrogenolysis of the benzyl ethers under continuous flow (0.3–1 mL/min) and H_2_ pressure (30–60 bar) using an H-cube apparatus was found to be effective at removing the benzyl ethers, but arduously slow (>48 h) from the limited surface-mediated interactions with the Pd/C cartridge. By comparison, batch hydrogenolysis at 1 atm pressure hydrogen afforded the fully deprotected trimanose **16** in just 24 h. To further reduce the total number of benzyl groups, the simpler peracylated TCA donor **4** was used to cap the oligosaccharide.

The benzyl-protected nitrogen proved unnecessary and was difficult to deprotect under these conditions. Therefore, future applications to automated syntheses will exclude this nitrogen protecting group. The light chain fluorous CbzF tag was advantageous in reducing the number of steps to a readily conjugateable molecule for particle functionalization compared to that of our previously used alkene fluorous tag [11,52–53]. The CbzF tag was also removed during global deprotection to readily provide an amine handle for conjugation to the dendrimeric core, avoiding further synthetic manipulations.

### Synthesis of glycodendrimer

**Scheme 3:**
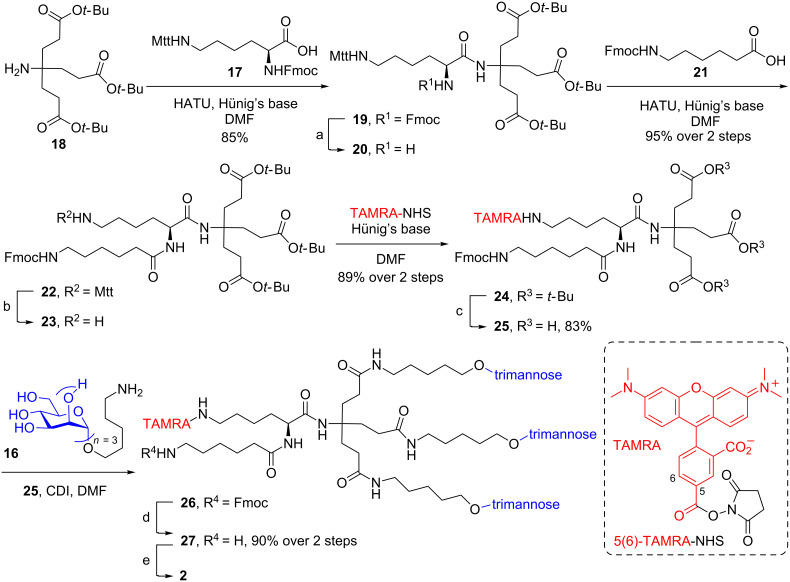
Synthesis of bioerodible microparticle **2**. Reagents and conditions: a) Et_2_NH, CH_3_CN, b) CH_2_Cl_2_/TFE/AcOH 3:1:1, c) TFA/THF/H_2_O 90:8:2, d) Et_2_NH, DMF, e) CPH/SA microparticles 20:80, EDCI, NHS, H_2_O.

Attachment of the 5(6)-TAMRA fluorophore to the dendrimeric core allowed for various potential linkage options in addition to providing the amine handle for microparticle surface functionalization. Unfortunately, the sterically encumbered tertiary amine of the dendrimer prevented simple coupling of commercially available *N*^α^*-*TAMRA-*N*^ε^-Teoc-L-lysine to the dendrimer. Therefore, we settled on first coupling *N*^α^-Fmoc-*N*^ε^-(4-methyltrityl)-L-lysine (**17**) to the dendrimeric amine **18** by standard HATU amide coupling conditions (Scheme 3).

The protecting groups were chosen specifically for selective deprotection and compatibility with the TAMRA fluorophore, which is stable to acidic and basic conditions, but incompatible with reducing conditions. To conserve the relatively expensive TAMRA fluorophore and prevent loss through the synthetic sequence, amino linkers were first conjugated to the dendrimer core. The *N*^α^-Fmoc group of **19** was first removed and coupled to *N*^ε^-Fmoc-aminohexanoic acid **21** to afford the elaborated dendrimer core **22**. The aminohexanoic acid would eventually provide the amino handle for the attachment to the surface of the microparticle and was considered sufficient in chain length to perform the functionalization. The 4-methyltrityl (Mtt) group was selectively hydrolyzed with CH_2_Cl_2_/TFE/AcOH 3:1:1 and trapped by triethylsilane, providing free amine **23** [54]. At this point, the epsilon amine **23** was coupled with TAMRA-NHS, followed by acidic deprotection of the *tert*-butyl esters to afford the triacid **25**. Then, the successful coupling of the deprotected trimannose **16** to the dendrimeric core **25** provided the glycodendrimer **26**. For the attachment of the glycodendrimer to the microparticle surface, the amino Fmoc was removed then coupled via EDCI/NHS to afford the desired bioerodible glycodendrimer microparticle **2**.

### In vivo assessment of bioerodible glycodendrimer

**Figure 4:**
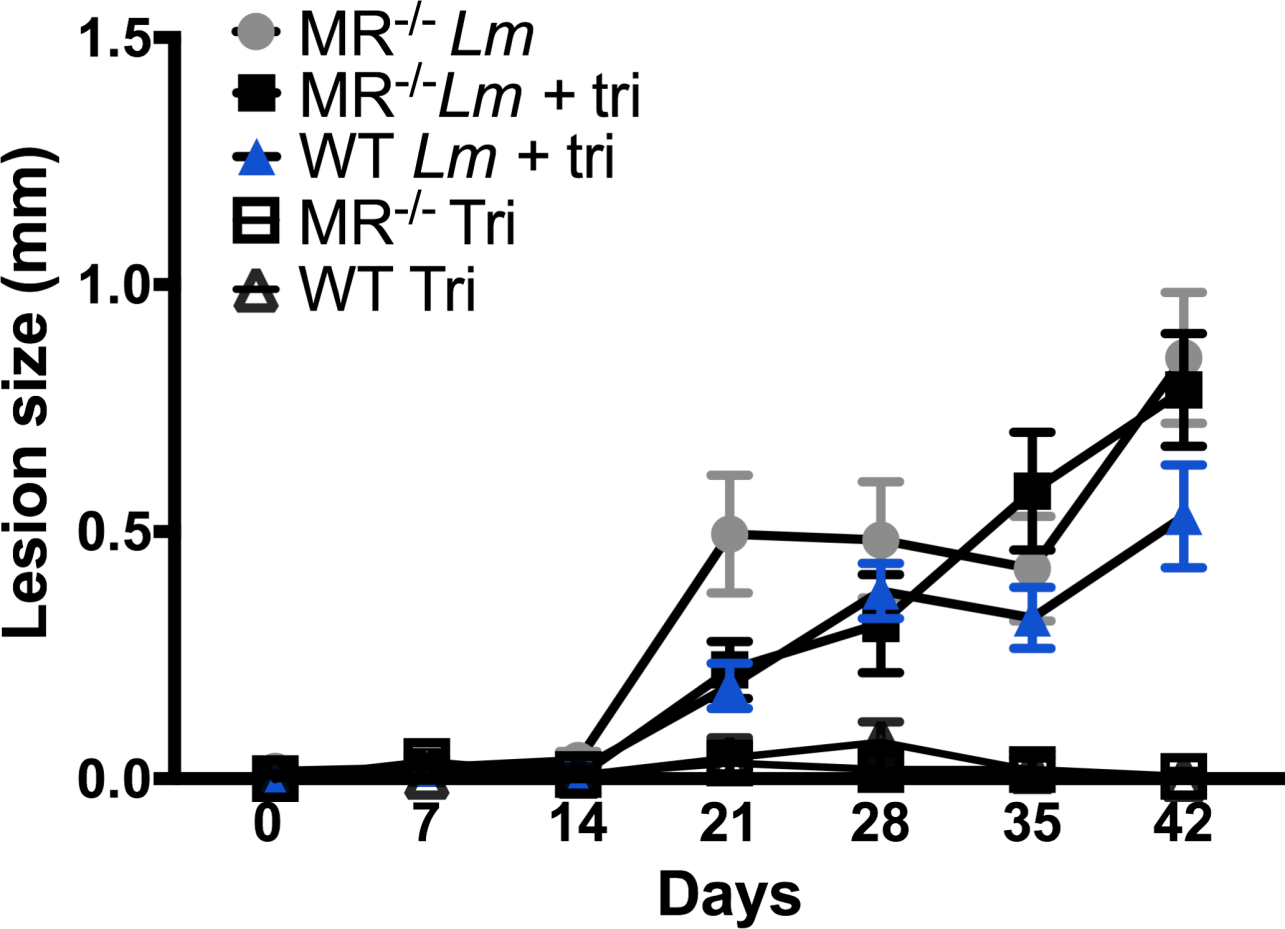
Trimannose-linked bioerodible microparticle **2** treatment of *Leishmania major* infected wild-type and mannose receptor deleted C57BL/6 mice reduces footpad lesion size. Footpad lesion size is in millimeters for wild-type (WT) and mannose receptor deleted (MR^−/−^) mice inoculated with *L. major* alone (*Lm*, grey circle MR^−/−^), *L. major* and trimannose bioerodible microparticles **2** (*Lm* + tri: blue triangle WT, black square MR^−/−^), or trimannose bioerodible microparticles **2** alone (Tri: unfilled squares MR^−/−^, unfilled triangles WT). Footpad lesion size was determined by calculating difference between inoculated left hind footpad and non-inoculated right hind footpad. *n* = 6–10 mice per group. One-way ANOVA with Tukey’s post-hoc test.

To evaluate the safety and feasibility of the trimannose bioerodible microparticles **2** in vivo during *L. major* infection, we inoculated C57BL/6 wild type (WT) and mannose receptor deficient (MR^−/−^) mice with *L. major* parasites alone, trimannose bioerodible microparticles **2** alone, or *L. major* parasites and trimannose bioerodible microparticles **2** in a single inoculation on day 0 in a manner similar to previous studies using trimannose-coated latex beads **1** [42]. Lesions were measured weekly and allowed to progress to day 42 post-infection to provide a time course comparable to previous studies [42]. Mice inoculated with bioerodible microparticles **2** in the absence of *L. major* parasites showed insignificant changes in footpad thickness indicating no untoward response to these particles (Figure 4, unfilled squares and triangles). This is consistent with prior studies which have demonstrated that polyanhydride microparticles do not cause significant levels of inflammation [55], as well as prior studies that the trimannose on a latex bead **1** is not inflammatory in the absence of an infection [40,42]. Fortunately, the newly designed probe with TAMRA did not elicit a large inflammatory response that would preclude its use as a biological probe.

As shown by Akilov et al. [56] and Grinnage-Pulley et al. [42], mannose receptor deleted mice develop *L. major* infections similar to wild-type mice. *L. major* infected MR ^−/−^ mice developed progressive footpad inflammation as early as 21 days post-infection that continued through 42 days post-infection (grey circles and black squares). MR^−/−^ mice infected with *L. major* and bioerodible microparticles **2** (black squares) had lesions of a similar size to MR^−/−^ mice given *L. major* alone. These mice do not have the carbohydrate specific receptor to recognize the trimannose and alter the inflammatory response, so no difference in footpad lesion from *L. major* alone was expected to occur in these mice as seen in previous studies [42].

WT mice receiving *L. major* and bioerodible microparticles **2** developed lesions, but these lesions were smaller, although not significantly different, than the sizes of lesions found in infected MR^−/−^ mice at day 42 post-infection. These results are consistent with findings using trimannose-coated latex beads **1** [42]. Infected, trimannose treated, WT mice developed lesions, but the lesion size was significantly decreased at peak infection, and these mice had smaller, but not significantly decreased lesions 42 days post-infection. Due to limited availability of MR^−/−^ mice, both WT and MR^−/−^ mice were older than in prior studies, which may have resulted in the non-significant lesion size difference. The similar decreased response in infected trimannose treated wild-type mice in both the latex bead **1** and bioerodible microparticle **2** studies suggests that the new trimannose-linked bioerodible microparticles **2** are a safe and viable tool for presenting glycodendrimers to mammalian immune systems.

The new construct of the bioerodible microparticle allows for investigation into the mechanisms of pathogen-recognition via MR and TLR2/4 and antigen presentation by APCs. Furthermore, it is anticipated that the glycodendrimer microparticles could provide spared dose vaccination or adjuvant therapy against *L. major* leishmaniasis, which will be evaluated in future work. Preliminary biological studies reported here show that indeed bioerodible microparticles **2** have an effect similar to our previously designed latex beads **1** [42] and should be safe for more extensive animal studies. Future studies using the bioerodible microparticles **2** will include optimization of the relative dosing of bioerodible microparticles **2** as compared to the trimannose-coated latex beads **1**. Previously trimannose-coated latex beads **1** were inoculated into mouse footpads at days 0, 7, 14 and 21 for a total of 2.0 × 10^8^ trimannose-coated beads **1** [42]. We designed the bioerodible microparticles **2** to allow a single delivery of trimannose from a total of 5 × 10^7^ particles, a four-fold lower level than what was used for the latex bead study [42], as studies by Huntimer and co-workers showed in 2013 that the CPH:SA polymer provided inherent adjuvant properties and dose sparing effects [57]. In light of these in vivo feasibility results, the first optimization studies should evaluate dosing for bioerodible microparticles **2** with single inoculations of 2×, 3×, and 4× higher amounts than the 5 × 10^7^ microparticle dose used here to see if a more significant change can be observed. Additional studies evaluating altered particle degradation rate(s) and encapsulation of trimannose within the particle could also be performed to provide sustained release of the trimannose.

## Conclusion

Herein we described our newly designed pathogen-associated bioerodible microparticle **2** for investigation into the mechanisms of *L. major* immune suppression and evasion. The fluorescent-tagged pathogen-associated oligosaccharide probe was synthesized through orthogonal conjugation for ease of attachment to bioerodible microparticles. The PAMP carbohydrate **16** was further synthesized through iterative glycosylation and FSPE purification to rapidly afford appreciable and pure quantities in fewer steps than prior work enabled by the Cbz F-tag that allows for solution-phase automated synthesis in the future.

In conclusion, these newly synthesized α-1,2-trimannose-linked bioerodible microparticles **2** are a feasible construct for delivering trimannose to *L. major* infected mice and will be a more translatable therapeutic target moving forward. We anticipate that further studies will allow for elucidation of the effect of these pathogen-associated molecular patterns of LPG to investigate the intracellular environment and mechanisms for immune suppression and evasion; the new construct of the bioerodible microparticle allows for investigation into the mechanisms of pathogen-recognition via MR and TLR2/4 and antigen presentation by APCs. Furthermore, it is anticipated that the glycodendrimer microparticles could provide spared dose vaccination or adjuvant therapy against *L. major* leishmaniasis, which will be evaluated in future work.

## Supporting Information

File 1 Experimental, characterization data and copies of spectra.
