# Supplementary material for: Design and synthesis of multivalent α-1,2-trimannose-linked bioerodible microparticles for applications in immune response studies of Leishmania major infection
Source: Beilstein J Org Chem. 2019 Mar 11;15:623–32. doi: 10.3762/bjoc.15.58 (PMC6423605; doi:10.3762/bjoc.15.58)

BEILSTEIN JOURNAL OF ORGANIC CHEMISTRY

Supporting Information

for

Design and synthesis of multivalent α-1,2-trimannose-linked bioerodible microparticles for applications in immune response studies of *Leishmania major* infection

Chelsea L. Rintelmann, Tara Grinnage-Pulley, Kathleen Ross, Daniel E. K. Kabotso, Angela Toepp, Anne Cowell, Christine Petersen, Balaji Narasimhan and Nicola Pohl

Beilstein J. Org. Chem. **2019,** *15,* 623–632. doi:10.3762/bjoc.15.58

Experimental, characterization data and copies of spectra

License and Terms: This is a supporting information file under the terms of the Creative Commons Attribution License (http://creativecommons.org/licenses/by/4.0). Please note that the reuse, redistribution and reproduction in particular requires that the authors and source are credited.
The license is subject to the *Beilstein Journal of Organic Chemistry* terms and conditions: (https://www.beilstein-journals.org/bjoc)

General experimental……………………………………………..……………………….**S2**

General experimental FSPE………………………………………………………………….**S3**

Synthetic procedures…………………………………………………………………S4–S28

Trimannose-linked bioerodible microparticles (2)…………………………………….S28

Figure S1: SEM images of microparticles……………………………………………..S29

Trimannose-coated latex beads (1)…………………………………………………….S29

In vivo experiments…………………………………………………………………….…S31

Experimental references……………………………………………………………S32–S33

^{1}H and ^{13}C NMR spectra………………………………………………………………..S34–S52

General experimental

The following procedures are reported on the largest scale reactions, regardless of yields. All non-aqueous reactions were conducted under argon atmosphere using standard Schlenk techniques for the exclusion of moisture and air. For all room temperature reactions, the ambient temperature was between 20–23 °C. All solvents were dried using 4 Å or 3 Å molecular sieves, or by a solvent drying system with basic alumina columns unless otherwise noted. Analytical thin layer chromatography was performed on Sorbtech silica gel 250 µm, using UV light as the visualizing agent and a solution of bromocresol green, cerium ammonium molybdate (CAM), $KMnO_4$, ninhydrin, *p*-anisaldehyde, or vanillin, as developing agents and then heat. Column chromatography was performed using Sorbtech 60 Å (230 × 400 mesh) or by Teledyne RediSep ISCO columns.

Compounds characterized by ^{1}H and ^{13}C spectra were recorded on 400 MHz, 500 MHz or 600 MHz Varian spectrometers. All chemical shifts are referenced to TMS or residual non-deuterated solvent ($CDCl_3$: ^{1}H NMR = 7.26 ppm, ^{13}C NMR = 77.16 ppm; $(CD_3)_2CO$: ^{1}H NMR = 2.05 ppm, ^{13}C NMR = 29.84, 206.26 ppm; CD_3CN: ^{1}H NMR = 1.94 ppm, ^{13}C NMR = 1.32, 118.26 ppm; D_2O: ^{1}H NMR = 4.79 ppm). Data for proton spectra are reported as follows: chemical shift (multiplicity [singlet (s), doublet (d), triplet (t), quartet (q) and multiplet (m)], coupling constants [Hz], integration, assignment). Carbon spectra were recorded with proton decoupling and the chemical shifts are reported in ppm (C) relative to TMS. Perfluorinated carbon signals on all Cbz-F containing compounds were not reported due to available NMR methods and low signal due to C–F splitting in ^{13}C NMR. All melting points are uncorrected.

High-resolution mass spectra (HRMS) were recorded on an Agilent 1200 HPLC-6130 MSD or Bruker Autoflex III MALDI-TOF mass spectrometers.

Analytical high performance liquid chromatography (HPLC) was performed on an Agilent 1100 series instrument with Photodiode Array (PDA) and Evaporative Light Scattering Detector (ELSD) detection. Analysis was carried out using a Phenomenex Luna® C18(2) reversed-phase column (5 µm particle size, 100 Å pore size, 250 mm length × 4.6 mm diameter), with mobile phases consisting of acetonitrile and water with 0.1% TFA at 1 mL/min flow rate. Preparative HPLC purifications were performed with an Agilent 1100 HPLC purification system using a Phenomenex Luna C18(2) reverse-phase column (5 µm particle size, 100 Å pore size, 250 mm length × 50 mm diameter).

FluoSpheres carboxylated-modified microspheres were purchased from ThermoFisher Scientific (Product #F8821, 1µm, red (580/605), 2% solids). 5(6)-TAMRA was purchased from AnaSpec as an isomeric mixture (Product# AS-81120-1).

General procedure for fluorous-solid phase extraction (F-SPE) [1]

F-SPE cartridges were manually packed with perfluorooctylethylsilyl ($Si(CH_2)_2C_8F_{17}$) (loading capacity 5–10% by weight). New F-SPE cartridges were prewashed with DMF (1 mL, first use only). To precondition the cartridge, it was washed with 80% aqueous MeOH (1 column volume) under vacuum without drying the column. Crude sample dissolved in 90% aqueous DMF (0.5 mL) was loaded onto the cartridge and left for 5 min to adsorb to the solid phase. The cartridge was then rinsed with a fluorophobic wash of 80% aqueous MeOH under vacuum until all non-fluorous compounds eluted. Fluorous compounds were then eluted with 100% MeOH or acetone under vacuum (note: fluorous fractions usually milky white and detergent-like in appearance). To

regenerate the column and elute remaining compounds, the cartridge was rinsed with acetone.

Synthetic procedures (in order of appearance)

4-(3,3,4,4,5,5,6,6,7,7,8,8,8-Tridecafluorooctyl)benzyl benzyl(5-hydroxypentyl) carbamate (7, CbzF) [2]

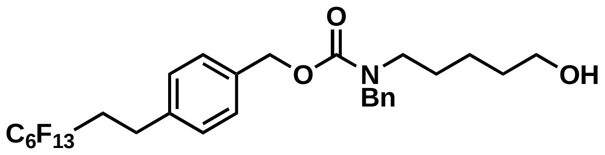

To a heterogeneous mixture *of N*-[4-(1*H*,1*H*,2*H*,2*H*-perfluorooctyl) benzyloxycarbonyloxy] succinimide **5** (1.9 g, 3.5 mmol) and $NaHCO_3$ (0.89 g, 10.6 mmol) in THF/H_2O 4:1 (50 mL) was added 5-Benzylamino-1-pentanol **6** (0.85 mL, 4.2 mmol). The reaction was left to stir for 2 h then concentrated with toluene azeotrope under reduced pressure. The carbamate **7** was purified by FSPE[6] to remove non-fluorous material followed by column chromatography [hexanes/EtOAc 2:1] to afford a white spherical solid (1.8 g, 2.7 mmol, 77%).

7: R_f = 0.35 [hexanes/EtOAc (2:1) (CAM)]; *N*-rotamers: ^{1}H NMR (500 MHz, $CDCl_3$) δ 7.39 – 7.06 (m, 9H), 5.15 (d, *J* = 15.9 Hz, 2H), 4.49 (d, *J* = 9.4 Hz, 2H), 3.57 (d, *J* = 25.8 Hz, 2H), 3.25 (d, *J* = 38.6 Hz, 2H), 2.91 (d, *J* = 8.9 Hz, 2H), 2.36 (dd, *J* = 18.0, 8.9 Hz, 2H), 1.53 (d, *J* = 20.8 Hz, 5H), 1.30 (d, *J* = 33.6 Hz, 2H); *N*-rotomers ^{13}C NMR (126 MHz, $CDCl_3$) δ 156.8, 156.4, 139.0, 138.0, 135.5, 135.4, 128.6, 128.5, 127.9, 127.4, 127.3, 67.0, 62.7, 50.6, 50.3, 47.1, 46.3, 33.2, 33.0 (t, $J_{C\text{-}F}$ = 22.2 Hz, benzylic), 32.8, 32.4, 28.0, 27.5, 26.3, 26.24 (t, $J_{C\text{-}F}$ = 4.3 Hz, homobenzylic), 26.2, 23.0; HRMS (TOF ESI) Calcd for $C_{28}H_{29}F_{13}NO_3$ $[M+H]^+$ 674.1940, found 674.1929.

1,2,3,4,6-Penta-*O*-acetyl-α/β-D-mannopyranose (i) [3]

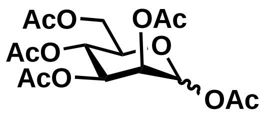

To a solution of D-mannose (25.0 g, 0.139 mol) in Ac_2O (125 mL, 1.318 mol) at 0 °C was added I_2 (1.4 g, 5.5 mmol). The reaction

mixture was left to stir for 4 h at ambient temperature, then quenched with saturated Na_2SO_3 (0.7 g, 5.5 mmol) and $NaHCO_3$, until no more CO_2 evolved and the solution became colorless. The organic product was extracted with CH_2Cl_2 (3 × 100 mL), and the collected organic layers were washed with $NaHCO_3$ (100 mL), dried over Na_2SO_4 and concentrated under reduced pressure with toluene azeotrope to afford a clear viscous oil (54.0 g, 0.14 mol, 98%, 2:1 mixture α:β). The product was used without further purification.

i: R_f = 0.4 [hexanes:EtOAc (1:1) (*p*-anisaldehyde)]; α anomer: ^{1}H NMR (500 MHz, $CDCl_3$) δ 6.08 (d, J = 1.9 Hz, 1H, H-1), 5.34 (d, J = 6.1 Hz, 1H, H-2), 5.27 – 5.25 (m, 1H, H-3), 4.28 (dd, J = 12.3, 4.8 Hz, 1H, H-6a), 4.12 – 4.05 (m, 2H, H-6b, H5), 2.18 (s, 3H), 2.17 (s, 3H), 2.09 (s, 3H), 2.06 (s, 3H), 2.01 (s, 3H); ^{13}C NMR (126 MHz, $CDCl_3$) δ 170.4, 169.9, 169.7, 169.5, 168.0, 90.6, 70.6, 68.9, 68.4, 65.7, 62.5, 62.1, 20.8, 20.7, 20.7, 20.6, 20.5.

3,4,6-Tri-*O*-acetyl-1,2-*O*-[1-(*exo*- and *endo*-methoxy)ethylidene]-β-D-manno pyranose (ii) [4–6]

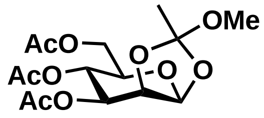

To a solution of *O*-peracylated mannose **i** (54.0 g, 140 mmol) in CH_2Cl_2 (100 mL) at 0 °C was added dropwise 33% HBr in AcOH (105 mL, 600 mmol). The reaction was allowed to stir for 6 h at 0 °C, then diluted with H_2O (100 mL). The organic product was extracted with CH_2Cl_2 (3 × 200 mL) and the collected organic layers were stirred in saturated $NaHCO_3$ (100 mL) until neutral pH was attained. The layers were separated, and the organic layer was dried over Na_2SO_4, filtered, and then concentrated under reduced pressure to afford a yellow oil, which was used without further purification.

To the solution of bromide (~57.7 g, 140 mmol) in CH_2Cl_2 (100 mL) was added tetrabutylammonium bromide (9.04 g, 30 mmol), triethylamine (39 mL, 280 mmol) and MeOH (23 mL, 560 mmol). The reaction mixture was then heated to 50 °C and left to stir for 17.5 h, then cooled to rt. The reaction mixture was concentrated to remove CH_2Cl_2 and MeOH. The crude residue was then extracted with EtOAc (3 × 200 mL) and H_2O (200 mL). The collected organic layers were washed with 5% aqueous LiCl (200 mL) and brine (200 mL). This was further dried over Na_2SO_4, filtered and concentrated under reduced pressure. The orthoester **ii** was purified by column chromatography [hexanes/EtOAc 2:1, 1% Et_3N] to afford a crystalline white solid (21.5 g, 60.0 mmol, 42% over two steps).

ii: R_f = 0.6 [hexanes:EtOAc (1:1) (CAM)]; mp 100-102 °C (EtOAc) (lit[7] 105-109 °C (EtOH); ^{1}H NMR (600 MHz, $CDCl_3$) δ 5.51 (d, J = 2.6 Hz, 1H, H-1), 5.29 (t, J = 9.7 Hz, 1H, H-4), 5.16 (dd, J = 9.9, 4.0 Hz, 1H, H-3), 4.62 (dd, J = 4.0, 2.6 Hz, 1H, H-2), 4.24 (dd, J = 12.2, 4.9 Hz, 1H, H-6a), 4.14 (dd, J = 12.2, 2.7 Hz, 1H, H-6b), 3.70 (ddd, J = 9.6, 4.9, 2.7 Hz, 1H, H-5), 3.28 (s, 3H, OMe), 2.12 (s, 3H, COMe), 2.07 (s, 3H, COMe), 2.06 (s, 3H, COMe), 1.74 (s, 3H, Me); ^{13}C NMR (126 MHz, $CDCl_3$) δ 170.6, 170.3, 169.4, 124.5, 97.4, 76.7, 71.3, 70.6, 65.5, 62.3, 49.8, 24.4, 20.7, 20.69, 20.65, 20.56.

3,4,6-Tri-*O*-benzyl-1,2-*O*-[1-(*exo*- and *endo*-methoxy)ethylidene]-β-D-mannopyranose (iii) [3]

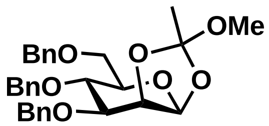

To a solution of 3,4,6-triacylated orthoester **ii** (21.45 g, 60.0 mmol) in methanol (100 mL) was added a catalytic amount of Na° metal (approximately 2 pea-sized chunks). The reaction mixture was left to stir for 3 h, and then concentrated under reduced pressure with toluene azeotrope to afford the triol.

To a solution of the triol (~15.6 g, 60 mmol) and BnBr (35 mL, 300 mmol), in MeCN (100 mL) at 0 °C was added NaH (8.5 g, 200 mmol) in one portion. The reaction was allowed to warm to room temperature and left to stir for 23 h. The reaction was then quenched with MeOH (8.6 mL), and concentrated under reduced pressure. The reaction mixture was extracted with EtOAc (3 × 200 mL) and H_2O (200 mL). The collected organic layers were washed with brine (300 mL) dried with Na_2SO_4, filtered and concentrated under reduced pressure. The orthoester **iii** was purified by column chromatography [hexanes/EtOAc 4:1, 1% Et_3N] to afford a spherical crystalline white solid (17.3 g, 34.0 mmol, 57% over two steps).

iii: R_f= 0.5 [hexanes:EtOAc (2:1) (*p*-anisaldehyde)]; mp 66-70 °C ($CHCl_3$) (lit[8] 75-77 °C (Et_2O); ^{1}H NMR (500 MHz, $CDCl_3$) δ 7.43 – 7.18 (m, 15H, Ph), 5.32 (d, *J* = 2.5 Hz, 1H, H-1), 4.89 (d, *J* = 10.8 Hz 1H, CH*H*Ph), 4.77 (ABq, *J* = 12.0 Hz, 2H, CH_2Ph), 4.64 – 4.48 (m, 3H, CH_2Ph; CH*H*Ph), 4.38 (dd, *J* = 4.0, 2.4 Hz, 1H, H-2), 3.92 (t, *J* = 9.4 Hz, 1H, H-4), 3.77 – 3.66 (m, 3H, H-3, H-6a,b), 3.40 (ddd, *J* = 9.3, 4.5, 2.2 Hz, 1H, H-5), 3.27 (s, 3H, OMe), 1.74 (s, 3H, Me); ^{13}C NMR (126 MHz, $CDCl_3$) δ 138.22, 138.20, 137.9, 137.8, 128.5, 128.4, 128.3, 128.0, 127.9, 127.7, 127.5, 124.0, 97.5, 79.0, 75.2, 74.2, 74.1, 73.3, 72.3, 69.0, 49.7, 24.4.

2-*O*-Acetyl-3,4,6-tri-*O*-benzyl-α/β-D-mannopyranose (iv) [9,10]

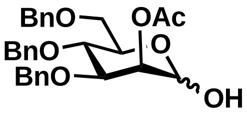

Perbenzylated orthoester **iii** (17.3 g, 34.0 mmol) was dissolved in 80% aqueous AcOH (100 mL) and stirred at room temperature for 15 h. The reaction mixture was concentrated under reduced pressure and residual AcOH and H_2O were removed by toluene azeotrope. The hemiacetal **iv** was purified by

column chromatography [gradient hexanes:EtOAc (1:0 to 1:1) with 1% Et_3N] to afford a clear colorless oil (14.9 g, 34.0 mmol, 98% yield).

iv: R_f = 0.3 [hexanes:EtOAc (3:1) (*p*-anisaldehyde)]; α,β anomers: ^{1}H NMR (400 MHz, $CDCl_3$) *δ* 7.38 – 7.10 (m, 15 H, Ph), 5.37 (dd, *J* = 3.3, 1.9 Hz, 1H, H-2), 5.21 (d, *J* = 1.9 Hz, 1H, H-1), 4.85 (d, *J* = 10.9 Hz, 1H, CH*H*Ph), 4.70 (d, *J* = 11.3 Hz, 1H, CH*H*Ph), 4.61 (d, *J* = 12.2 Hz, 1H, CH*H*Ph), 4.53 (d, *J* = 11.2 Hz, 1H, CH*H*Ph), 4.50 (d, *J* = 12.1 Hz, 1H, CH*H*Ph), 4.46 (d, *J* = 10.8 Hz, 1H, CH*H*Ph), 4.05 (m, 2H, H-3; H-5), 3.75 (t, *J* = 9.7 Hz, 1H, H-4), 3.69 (d, *J* = 3.9 Hz, 2H, H-6a,b), 2.14 (s, 3H, COMe); α,β anomers: ^{13}C NMR (126 MHz, $CDCl_3$) *δ* 170.9, 170.5, 138.3, 138.1, 137.9, 137.8, 137.7, 137.5, 128.4, 128.36, 128.34, 128.3, 128.1, 128.0, 127.9, 127.7, 127.6, 93.1, 92.4, 80.3, 77.6, 75.1, 75.0, 74.6, 73.9, 73.5, 73.4, 71.8, 71.7, 71.0, 69.3, 69.2, 69.1, 69.0, 21.1.

2-*O*-Acetyl-3,4,6-tri-*O*-benzyl-α-D-mannopyranosyl trichloroacetimide (3) [10,11]

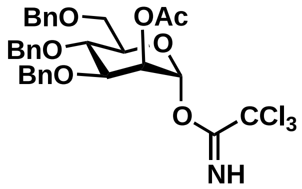

To a solution of hemiacetal **iv** (14.9 g, 34.0 mmol) in CH_2Cl_2 (70 mL) at 0 °C was added trichloroacetonitrile (20.5 mL, 200 mmol) followed by 1,8-diazabicyclo[5.4.0]undec-7-ene (DBU) (2.0 mL, 13 mmol). The reaction mixture was allowed to warm to room temperature and stirred for 21 h. The reaction mixture was concentrated under reduced pressure and purified by column chromatography [hexanes/EtOAc 3:1, 0.5% Et_3N] to afford a yellow viscous oil (18.03 g, 28.0 mmol, 82%).

3: R_f = 0.6 [hexanes:EtOAc (2:1) (*p*-anisaldehyde)]; ^{1}H NMR (600 MHz, $CDCl_3$) *δ* 8.68 (s, 1H, NH), 7.39 – 7.13 (m, 15H, Ph), 6.32 (d, *J* = 2.0 Hz, 1H, H-1), 5.51 (d, *J* = 2.4 Hz, 1H, H-2), 4.87 (d, *J* = 10.5 Hz, 1H, CH*H*Ph), 4.73 (d, *J* = 11.2 Hz, 1H, CH*H*Ph), 4.66 (d, *J* = 12.0 Hz, 1H, CH*H*Ph), 4.57 (d, *J* = 11.4 Hz, 1H, CH*H*Ph), 4.53 (d, *J* = 10.5 Hz, 1H,

CH*H*Ph), 4.49 (d, *J* = 11.9 Hz, 1H, CH*H*Ph), 4.08 – 3.96 (m, 3H, H-3; H-4; H-5), 3.83 (dd, *J* = 11.2, 3.9 Hz, 1H, H-6a), 3.70 (d, *J* = 11.5 Hz, 1H, H-6b), 2.16 (s, 3H, OAc); ^{13}C NMR (126 MHz, $CDCl_3$) *δ* 170.0, 159.9, 138.2, 138.1, 137.5, 128.4, 128.35, 128.3, 128.27, 128.0, 127.9, 127.8, 127.6, 127.57, 95.4, 90.8, 75.4, 74.38, 73.7, 73.4, 72.1, 68.4, 67.31, 53.4, 21.0.

2,3,4,6-Tetra-*O*-acetyl-α-D-mannopyranosyl trichloroacetimidate (4) [3]

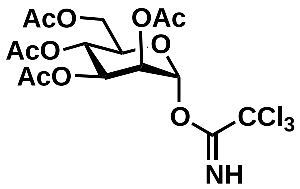

To a solution of peracylated mannose **i** (18.3 g, 46.9 mmol) in DMF (50 mL) was added hydrazine acetate (4.9 g, 52 mmol). The reaction was left to stir for 1 h, then suspended in CH_2Cl_2 (300 mL). The organic layer was washed with cold saturated $NaHCO_3$ (500 mL), dried over $MgSO_4$, and concentrated under reduced pressure.

The hemiacetal was resuspended in CH_2Cl_2 (100 mL) and cooled to 0 °C. To this stirred mixture was added trichloroacetonitrile (47 mL, 470 mmol) followed by 1,8-diazabicyclo[5.4.0]undec-7-ene (DBU, 1.75 mL, 11.7 mmol). The reaction was allowed to warm to room temperature then left to stir for 24 h. The reaction was concentrated under reduced pressure, and purified by column chromatography [hexanes/EtOAc 1:1, 0.5% Et_3N) to afford a yellow viscous oil (20.0 g, 40.6 mmol, 87% over 2 steps).

4: R_f = 0.6 [hexanes:EtOAc (1:1) (*p*-anisaldehyde)]; ^{1}H NMR (500 MHz, $CDCl_3$) *δ* 8.83 (s, 1H, NH), 6.28 (d, *J* = 1.9 Hz, 1H, H-1), 5.50 – 5.45 (m, 1H, H-2), 5.43 – 5.36 (m, 2H, H-3, H-4), 4.28 (dd, *J* = 12.1, 4.8 Hz, 1H, H-6a), 4.23 – 4.13 (m, 2H, H-6b, H-5), 2.20 (s, 3H, COC*H₃*), 2.08 (s, 3H, COC*H₃*), 2.07 (s, 3H, COC*H₃*), 2.01 (s, 3H, COC*H₃*); ^{13}C NMR (126 MHz, $CDCl_3$) *δ* 170.5, 169.8, 169.7, 169.6, 163.6, 159.7, 137.8, 129.0, 128.2,

125.3, 94.5, 92.0, 90.5, 71.2, 68.8, 67.8, 65.4, 62.0, 20.74, 20.7, 20.6; HRMS (TOF ESI) Calcd for $C_{16}H_{20}Cl_3NO_{10}Na$ $[M+Na]^+$ 514.0050, found 514.0043.

_N_-(Benzyl)-_N_-(4-(3,3,4,4,5,5,6,6,7,7,8,8,8-tridecafluorooctyl)benzyloxycarbonyl)-5-aminopent-1-yl 2-_O_-acetyl-3,4,6-tri-_O_-benzyl-α-D-mannopyranoside (8)

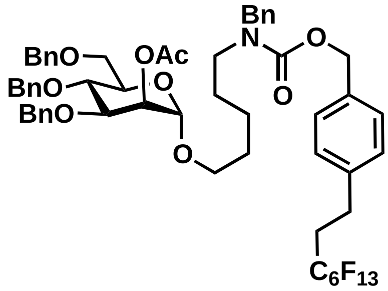

To alcohol **7** (1.82 g, 2.71 mmol) in CH_2Cl_2 (3 mL) was added mannosyl TCA donor **3** (3.5 g, 5.4 mmol) in CH_2Cl_2 (7.4 mL). The reaction mixture was cooled to 0 °C prior to adding TMSOTf (98 µL, 0.54 mmol). After 30 min the reaction was allowed to warm to room temperature and was aged for an additional 1 h. The reaction was quenched with Et_3N (0.5 mL) then concentrated with toluene azeotrope under reduced pressure. The mannosyl glycoside **8** was purified by FSPE then column chromatography [hexanes/EtOAc 4:1, 1% Et_3N] to afford a clear oil (2.8 g, 2.4 mmol, 90%).

8: R_f = 0.35 [hexanes:EtOAc (3:1) (CAM)]; *N*-rotomers: ^1H NMR (500 MHz, $CDCl_3$) δ 7.34 – 7.07 (m, 26H), 5.30 (s, 1H), 5.10 (d, *J* = 18.2 Hz, 2H), 4.81 (d, *J* = 10.7 Hz, 1H), 4.76 (s, 1H), 4.64 (t, *J* = 12.8, 11.9 Hz, 2H), 4.49 (d, *J* = 9.6 Hz, 2H), 4.47 – 4.40 (m, 3H), 3.92 (dd, *J* = 9.2, 3.2 Hz, 1H), 3.83 (t, *J* = 9.4 Hz, 1H), 3.78 – 3.68 (m, 2H), 3.65 (d, *J* = 8.9 Hz, 1H), 3.58 (bs, 1H), 3.31 (bs, 1H), 3.18 (d, *J* = 34.2 Hz, 2H), 2.85 (t, *J* = 8.3 Hz, 2H), 2.30 (dd, *J* = 18.5, 9.6 Hz, 2H), 2.10 (s, 3H), 1.48 (d, *J* = 23.1 Hz, 4H), 1.22 (s, 2H); *N*-rotomers ^{13}C NMR (126 MHz, $CDCl_3$) δ 170.7, 156.8, 156.3, 139.0, 138.5, 138.4, 138.1, 135.6, 135.5, 128.7, 128.5, 128.49, 128.45, 128.42, 128.2, 128.1, 127.9, 127.8, 127.77, 127.7, 127.4, 127.3, 97.8, 78.4, 75.4, 74.5, 73.6, 71.9, 71.5, 69.0, 68.97, 67.8, 66.9, 50.7, 50.4, 47.3, 46.3, 33.02 (t, $J_{C\text{-}F}$ = 22.02 Hz), 29.2, 28.1, 27.6, 26.28 (t $J_{C\text{-}F}$ = 4.31 Hz), 23.5, 21.25; HRMS (TOF ESI) Calcd for $C_{57}H_{58}F_{13}NO_9Na$ $[M+Na]^+$ 1170.3802,

found 1170.3789.

N-(Benzyl)-N-(4-(3,3,4,4,5,5,6,6,7,7,8,8,8-tridecafluorooctyl)benzyloxycarbonyl)-5-aminopent-1-yl 3,4,6-tri-O-benzyl-α-D-mannopyranoside (9)

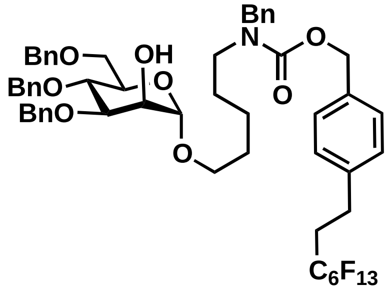

A solution of mannosyl glycoside **8** (2.8 g, 2.4 mmol) in 1 M methanolic NaOH (8 mL) was stirred for 1 h then quenched with Dowex resin (50WX8 H^+) until neutral pH was attained. The reaction mixture was filtered and the filtrate was concentrated under reduced pressure and purified by FSPE, then column chromatography [hexanes/EtOAc 2:1, 1% Et_3N] to afford a clear oil (1.64 g, 1.48 mmol, 55%).

9: R_f = 0.48 [hexanes:EtOAc (2:1) (CAM)]; *N*-rotomers: ^{1}H NMR (500 MHz, $CDCl_3$) δ 7.59 – 6.72 (m, 24H), 5.15 (d, J = 15.8 Hz, 2H), 4.86 (s, 1H, H-1), 4.81 (d, J = 10.9 Hz, 1H), 4.73 – 4.64 (m, 2H), 4.64 (d, J = 11.8 Hz, 1H), 4.56 – 4.49 (m, 2H), 4.51 – 4.45 (m, 2H), 4.01 (s, 1H), 3.84 (d, J = 11.9 Hz, 2H), 3.78 – 3.65 (m, 2H), 3.64 (bs, 1H), 3.36 (bs, 1H), 3.22 (d, J = 34.3 Hz, 2H), 2.97 – 2.82 (m, 2H), 2.45 (s, 1H, OH), 2.42 – 2.30 (m, 2H), 1.53 (s, 4H), 1.26 (s, 2H); *N*-rotomers ^{13}C NMR (126 MHz, $CDCl_3$) δ 156.8, 156.3, 139.0, 138.4, 138.1, 135.6, 135.5, 128.7, 128.6, 128.6, 128.5, 128.4, 128.1, 128.0, 127.95, 127.9, 127.8, 127.7, 127.4, 127.3, 99.3, 80.5, 75.3, 74.5, 73.6, 72.1, 71.2, 69.1, 68.6, 67.6, 67.0, 50.4, 47.3, 46.3, 33.0 (t, J_{C-F} = 22.0 Hz), 29.8, 29.2, 28.1, 27.6, 26.3 (t, J_{C-F} = 4.3 Hz), 23.6; HRMS (TOF ESI) Calcd for $C_{55}H_{56}F_{13}NO_8Na$ $[M+Na]^+$ 1128.3696, found 1128.3680.

N-(Benzyl)-N-(4-(3,3,4,4,5,5,6,6,7,7,8,8,8-tridecafluorooctyl)benzyloxycarbonyl)-5-aminopent-1-yl (2-O-acyl-3,4,6-tri-O-benzyl-α-D-mannopyranosyl)-(1→2)-O-3,4,6-tri-O-benzyl-α-D-mannopyranoside (10)

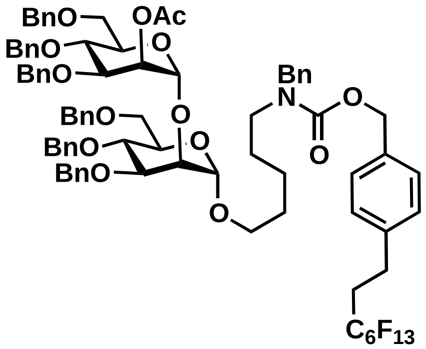

To neat alcohol **9** (1.64 g, 1.48 mmol) was added mannosyl TCA donor **3** (1.89 g, 2.97 mmol) in CH_2Cl_2 (7.4 mL). The reaction mixture was cooled to 0 °C prior to adding TMSOTf (55 µL, 0.3 mmol). The reaction mixture was allowed to warm to room temperature after 10 min and was aged for 2 h. The reaction was then quenched with Et_3N (0.5 mL) and concentrated under reduced pressure with toluene azeotrope. The disaccharide was purified by FSPE then column chromatography [gradient hexanes/EtOAc 4:1 to 2:1], 1% Et_3N] to afford a clear oil (2.2 g, 1.4 mmol, 94%).

10: R_f = 0.5 [hexanes:EtOAc (3:1) (CAM)]; N-rotomers ^{1}H NMR (500 MHz, $CDCl_3$) δ 7.53 – 6.86 (m, 42H), 5.53 (dd, J = 3.3, 1.9 Hz, 1H), 5.14 (d, J = 17.0 Hz, 2H), 5.07 (d, J = 1.8 Hz, 1H), 4.85 – 4.82 (m, 3H), 4.69 – 4.60 (m, 5H), 4.53 (t, J = 11.9 Hz, 2H), 4.46 (dd, J = 11.4, 7.5 Hz, 4H), 4.39 (d, J = 11.0 Hz, 1H), 4.01 – 3.92 (m, 3H), 3.87 (s, 1H), 3.82 (t, J = 9.3 Hz, 2H), 3.78 – 3.65 (m, 5H), 3.54 (bs, 1H), 3.18 (d, J = 31.0 Hz, 3H), 2.93 – 2.84 (m, 2H), 2.40 – 2.28 (m, 2H), 2.11 (s, 3H), 1.47 (s, 4H), 1.21 (s, 2H); N-rotomers ^{13}C NMR (126 MHz, $CDCl_3$) δ 170.2, 139.0, 138.7, 138.5, 138.4, 138.1, 128.7, 128.65, 128.5, 128.5, 128.46, 128.4, 128.4, 128.38, 128.3, 128.2, 128.16, 128.0, 127.9, 127.88, 127.8, 127.7, 127.6, 127.61, 127.58, 127.5, 127.3, 99.7, 98.8, 79.9, 78.3, 75.2, 75.15, 74.8, 74.5, 73.5, 73.4, 72.2, 72.0, 72.0, 71.9, 69.5, 69.3, 68.9, 67.6, 66.9, 50.6, 50.4, 47.3, 46.3, 33.04 (t, $J_{C\text{-}F}$ = 21.9 Hz), 29.4, 27.7, 26.3, 23.6, 21.3; HRMS (TOF ESI) Calcd for $C_{84}H_{86}F_{13}NO_{14}Na$ $[M+Na]^+$ 1602.5738, found 1602.5730.

N-(Benzyl)-N-(4-(3,3,4,4,5,5,6,6,7,7,8,8,8-tridecafluorooctyl)benzyloxycarbonyl)-5-aminopent-1-yl (3,4,6-tri-O-benzyl-α-D-mannopyranosyl)-(1→2)-O-3,4,6-tri-O-benzyl-α-D-mannopyranoside (11)

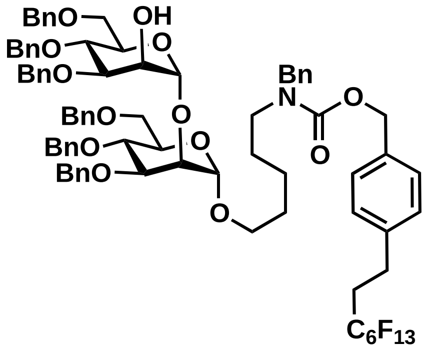

A solution of disaccharide **10** (2.2 g, 1.4 mmol) in 1 M methanolic NaOH (10 mL) was stirred for 1 h then quenched with Dowex resin (50WX8 H^+) until neutral pH was attained. The reaction mixture was filtered and the filtrate was concentrated under reduced pressure and purified by FSPE, then column chromatography [gradient hexanes/EtOAc 3:1 to 1:1, 1% Et_3N] to afford a clear oil (1.91 g, 1.24 mmol, 84%).

11: R_f = 0.46 [hexanes:EtOAc (2:1) (CAM)]; *N*-rotomers ^{1}H NMR (500 MHz, $CDCl_3$) δ 7.44 – 6.91 (m, 41H), 5.18 – 5.10 (m, 3H), 4.87 (d, *J* = 1.8 Hz, 1H, H-1), 4.82 (t, *J* = 9.7 Hz, 2H), 4.71 – 4.61 (m, 3H), 4.62 – 4.42 (m, 8H), 4.12 (s, 1H), 3.99 (s, 1H), 3.95 (d, *J* = 9.9 Hz, 1H), 3.92 – 3.75 (m, 5H), 3.73 – 3.65 (m, 4H), 3.52 (bs, 1H), 3.20 (bs, 3H), 2.94 – 2.84 (m, 2H), 2.42 – 2.24 (m, 3H), 1.46 (bs, 4H), 1.21 (bs, 3H); *N*-rotomers ^{13}C NMR (126 MHz, $CDCl_3$) δ 138.8, 138.3, 137.9, 128.5, 128.47, 128.4, 128.35, 128.3, 128.27, 128.0, 127.9, 127.8, 127.79, 127.7, 127.6, 127.5, 127.4, 127.36, 101.1, 98.8, 80.0, 79.8, 75.1, 75.0, 74.8, 74.4, 73.4, 73.3, 72.3, 72.1, 72.0, 71.5, 69.4, 68.5, 67.5, 66.8, 29.2, 26.2, 23.4; HRMS (TOF ESI) Calcd for $C_{82}H_{84}F_{13}NO_{13}Na$ $[M+Na]^+$ 1560.5633, found 1560.5616.

N-(Benzyl)-N-(4-(3,3,4,4,5,5,6,6,7,7,8,8,8-tridecafluorooctyl)benzyloxycarbonyl)-5-aminopent-1-yl [(2,3,4,6-tetra-O-acyl-α-D-mannopyranosyl)-(1→2)-O-(3,4,6-tri-O-benzyl-α-D-mannopyranosyl)]-(1→2)-O-3,4,6-tri-O-benzyl-α-D-mannopyranoside (12)

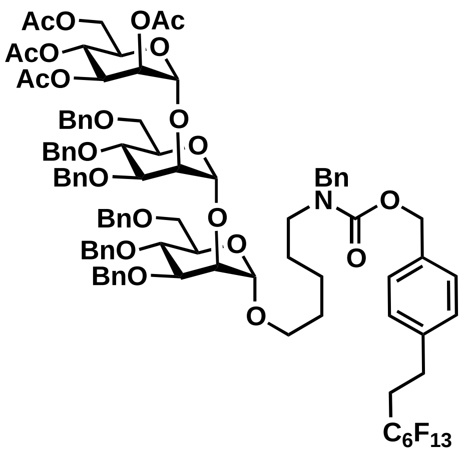

To dimannoside **11** (1.91 g, 1.24 mmol) in CH_2Cl_2 (8 mL) was added TCA donor **4** (1.84 g, 3.73 mmol) in CH_2Cl_2 (1.84 mL). The reaction mixture was cooled to 0 °C prior to adding TMSOTf (45 µL, 0.25 mmol). The reaction mixture was allowed to warm to room temperature after 10 min and was aged for 2 h. The reaction was then quenched with Et_3N (0.7 mL) and concentrated under reduced pressure with toluene azeotrope. The disaccharide was purified by FSPE then column chromatography [gradient hexanes/EtOAc 3:1 to 2:1, 1% Et_3N] to afford a clear oil (2.0 g, 1.1 mmol, 86%).

12: R_f = 0.5 [hexanes:EtOAc (2:1) (CAM)]; ^{1}H NMR (500 MHz, $CDCl_3$) δ 7.39 – 7.10 (m, 39H), 5.43 (dd, *J* = 3.5, 1.8 Hz, 1H), 5.40 (dd, *J* = 9.8, 3.4 Hz, 1H), 5.26 (t, *J* = 9.9 Hz, 1H), 5.17 (d, *J* = 2.0 Hz, 1H), 5.13 (d, *J* = 17.4 Hz, 2H), 4.89 (s, 1H), 4.88 (d, *J* = 1.8 Hz, 1H), 4.82 (dd, *J* = 10.9, 7.5 Hz, 2H), 4.71 – 4.42 (m, 14H), 4.16 – 4.07 (m, 2H), 4.03 – 3.64 (m, 16H), 3.50 (bs, 1H), 3.18 (d, *J* = 29.7 Hz, 3H), 2.93 – 2.84 (m, 2H), 2.34 (tt, *J* = 17.9, 8.4 Hz, 2H), 2.12 (s, 3H), 2.00 (s, 3H), 2.00 (s, 3H), 1.97 (s, 3H), 1.54 – 1.37 (m, 5H), 1.24 – 1.09 (m, 3H); ^{13}C NMR (126 MHz, $CDCl_3$) δ 170.5, 169.7, 169.7, 169.6, 138.8, 138.6, 138.4, 138.3, 137.9, 128.5, 128.4, 128.37, 128.33, 128.29, 128.27, 128.0, 127.9, 127.7, 127.6, 127.6, 127.5, 127.49, 127.46, 127.42, 127.1, 100.6, 99.2, 98.7, 79.7, 79.3, 75.7, 75.2, 75.0, 74.9, 73.4, 73.1, 72.5, 72.3, 72.2, 71.9, 69.6, 69.5, 69.3, 69.2, 68.9, 67.5, 66.8, 66.1, 62.2, 50.5, 50.2, 47.2, 46.1, 32.9, 32.7, 29.7, 29.2, 28.0,

27.5, 26.2, 23.4, 20.9, 20.8, 20.7, 20.6; HRMS (TOF ESI) Calcd for $C_{96}H_{102}NO_{22}F_{13}Na$ $[M+Na]^+$ 1890.6584, found 1890.6597.

_N_-(Benzyl)-_N_-(4-(3,3,4,4,5,5,6,6,7,7,8,8,8-tridecafluorooctyl)benzyloxycarbonyl)-5-aminopent-1-yl [(2-_O_-acyl-3,4,6-tri-_O_-benzyl-α-D-mannopyranosyl)-(1→2)-_O_-(3,4,6-tri-_O_-benzyl-α-D-mannopyranosyl)]-(1→2)-_O_-3,4,6-tri-_O_-benzyl-α-D-mannopyranoside (13)

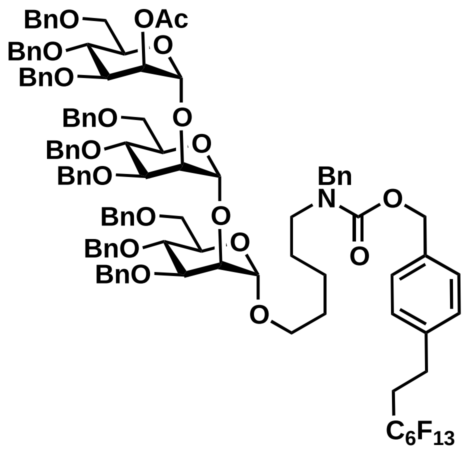

To a stirring solution of alcohol **11** (0.155 g, 0.100 mmol) in CH_2Cl_2 (5 mL) was added mannosyl TCA donor **3** (0.194 g, 0.304 mmol) in CH_2Cl_2 (1.7 mL). The reaction mixture was cooled to 0 °C and left to stir for 10 min prior to adding TMSOTf (5 µL, 0.03 mmol). After 30 min the reaction mixture was allowed to warm to room temperature, then aged for 2 h. The reaction was quenched with Et_3N (0.5 mL) and concentrated with toluene azeotrope under reduced pressure. The trisaccharide **13** was purified by FSPE, then column chromatography [hexanes/EtOAc 4:1, 1% Et_3N] to afford a clear oil (0.113 g, 0.056 mmol, 58%).

13: R_f = 0.48 [hexanes:EtOAc (4:1) (CAM)]; *N*-rotomers ^{1}H NMR (500 MHz, $CDCl_3$) *δ* 7.38 – 7.09 (m, 55H), 5.53 (dd, *J* = 3.3, 1.9 Hz, 1H), 5.18 (d, *J* = 2.0 Hz, 1H), 5.13 (d, *J* = 15.6 Hz, 2H), 5.04 (d, *J* = 1.9 Hz, 1H), 4.88 (d, *J* = 1.9 Hz, 1H), 4.85 – 4.78 (m, 3H), 4.70 – 4.37 (m, 18H), 4.30 (d, *J* = 12.1 Hz, 1H), 4.08 (t, *J* = 2.4 Hz, 1H), 3.98 (m, 1H) 3.96 – 3.87 (m, 5H), 3.82 (t, *J* = 9.2 Hz, 1H), 3.79 – 3.62 (m, 8H), 3.52 (d, *J* = 10.5 Hz, 2H), 3.16 (d, *J* = 30.0 Hz, 3H), 2.93 – 2.81 (m, 2H), 2.41 – 2.27 (m, 2H), 2.12 (s, 3H), 1.45 (s, 4H), 1.19 (s, 3H); *N*-rotomers ^{13}C NMR (126 MHz, $CDCl_3$) *δ* 170.1, 138.8, 138.6, 138.5, 138.4, 138.39, 138.2, 138.1, 128.5, 128.4, 128.3, 128.32, 128.29, 128.26,

128.25, 128.2, 128.16, 128.0, 127.97, 127.8, 127.7, 127.7, 127.6, 127.55, 127.50, 127.48, 127.4, 127.38, 127.37, 100.6, 99.4, 98.8, 79.6, 78.1, 77.2, 75.2, 75.1, 75.0, 74.9, 74.8, 74.3, 73.3, 73.3, 73.2, 72.2, 72.1, 71.9, 71.89, 71.87, 69.6, 69.4, 68.8, 68.7, 67.5, 66.8, 50.5, 50.2, 47.2, 46.2, 32.9 (t, $J_{C\text{-}F}$ = 22.07 Hz) 29.3, 28.0, 27.5, 26.2, 26.1, 23.4, 21.2; HRMS (TOF ESI) Calcd for $C_{111}H_{114}F_{13}NO_{19}Na$ $[M+Na]^+$ 2034.7675, found 2034.7705.

***N*-(Benzyl)-*N*-(4-(3,3,4,4,5,5,6,6,7,7,8,8,8-tridecafluorooctyl)benzyloxycarbonyl)-5-aminopent-1-yl [(3,4,6-tri-*O*-benzyl-α-D-mannopyranosyl)-(1→2)-*O*-(3,4,6-tri-*O*-benzyl-α-D-mannopyranosyl)]-(1→2)-*O*-3,4,6-tri-*O*-benzyl-α-D-mannopyranoside (15)**

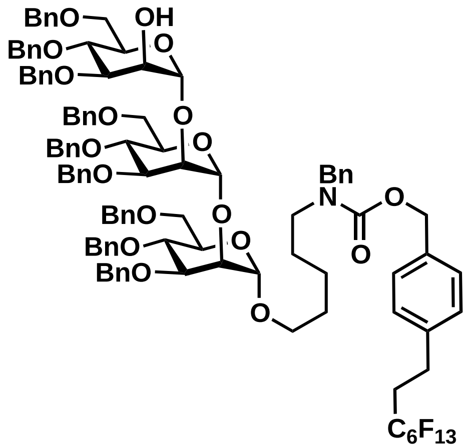

To a solution of trisaccharide **13** (0.097 g, 0.048 mmol) in MeOH (5 mL) was added a catalytic amount of Na^0 (peas-sized spatula tip). Upon reaction completion (2 h), the reaction mixture was quenched with Dowex resin (50WX8 H^+) until neutral pH was attained. The reaction mixture was filtered and the filtrate was concentrated under reduced pressure with toluene azeotrope and purified by FSPE then column chromatography [hexanes/EtOAc 3:1, 1% Et_3N] to afford a clear oil (0.089 g, 0.045 mmol, 93%).

15: R_f = 0.6 [hexanes:EtOAc (2:1) (CAM)]; *N*-rotomers ^{1}H NMR (500 MHz, $CDCl_3$) δ 7.42 – 7.06 (m, 55H), 5.20 (d, *J* = 2.0 Hz, 1H), 5.17 – 5.09 (m, 3H), 4.89 (d, *J* = 1.8 Hz, 1H), 4.81 (dd, *J* = 10.9, 8.0 Hz, 3H), 4.65 (d, *J* = 12.5 Hz, 2H), 4.62 – 4.41 (m, 15H), 4.32 (d, *J* = 12.2 Hz, 1H), 4.13 – 4.08 (m, 2H), 3.98 – 3.64 (m, 15H), 3.62 (dd, *J* = 10.6, 3.5 Hz, 1H), 3.56 – 3.49 (m, 2H), 3.16 (d, *J* = 31.4 Hz, 3H), 2.92 – 2.85 (m, 2H), 2.35 (q, *J* = 9.5 Hz, 3H), 1.45 (s, 4H), 1.19 (s, 2H); *N*-rotomers ^{13}C NMR (126 MHz, $CDCl_3$) δ

156.7, 156.1, 138.8, 138.6, 138.5, 138.4, 138.3, 138.2, 138.1, 137.9, 135.3, 128.9, 128.5, 128.46, 128.43, 128.4, 128.3, 128.3, 128.28, 128.26, 128.24, 128.0, 127.9, 127.82, 127.80, 127.76, 127.7, 127.60, 127.55, 127.48, 127.46, 127.44, 127.4, 127.1, 101.0, 100.8, 98.8, 80.0, 79.5, 79.4, 75.3, 75.2, 75.0, 74.98, 74.94, 74.9, 74.3, 73.3, 73.28, 73.24, 72.3, 72.2, 72.1, 71.8, 71.6, 69.6, 69.4, 68.9, 68.6, 67.5, 66.8, 50.5, 50.2, 47.2, 46.2, 33.1, 32.9, 32.7, 29.7, 29.3, 28.0, 27.6, 26.2, 23.4; HRMS (TOF ESI) Calcd for $C_{109}H_{112}F_{13}NO_{18}Na$ $[M+Na]^+$ 1992.7570, found 1992.7521.

5-Aminopentyl α-D-mannopyranosyl-(1→2)-α-D-mannopyranosyl-(1→2)-α-D-mannopyranoside (16)

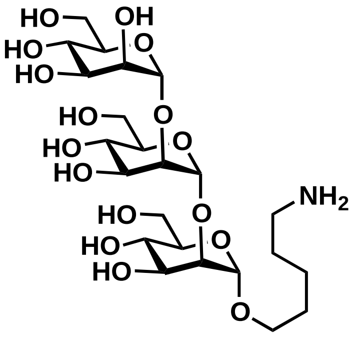

From 12: A solution of trimannose **12** (0.72 g, 0.39 mmol) in 1 M methanolic NaOH (5 mL) was stirred for 2 h, and then quenched with Dowex resin (50WX8 H^+) until neutral pH was attained. The reaction mixture was filtered and the filtrate was concentrated under reduced pressure with toluene azeotrope. The tetraol **14** was used without further purification.

From 14 or 15: A solution of protected trimannose **15** (0.85 g, 0.50 mmol) and 10% Pd/C (0.85 g) in [TFE/H_2O 9:1 1% HCO_2H] (25 mL) was stirred under H_2 atmosphere (1 atm) for 24 h. The crude reaction mixture was filtered through a plug of celite with [MeOH/H_2O 4:1] to remove Pd/C. The filtrate was concentrated under reduced pressure then freeze-dried to afford a yellow oil (0.28 g, 0.47 mmol, 94%).

16: ^1H NMR (500 MHz, D_2O) δ 8.41 (s, 2H), 5.31 (d, J = 1.9 Hz, 1H), 5.11 (d, J = 1.7 Hz, 1H), 5.06 (d, J = 1.8 Hz, 1H), 4.13 (dd, J = 3.3, 1.8 Hz, 1H), 4.08 (dd, J = 3.3, 1.8 Hz, 1H), 4.02 (q, J = 9.2 Hz, 1H), 3.99 – 3.88 (m, 7H), 3.86 (dd, J = 9.6, 3.3 Hz, 1H), 3.82 – 3.71 (m, 8H), 3.70 (d, J = 3.9 Hz, 1H), 3.68 (d, J = 4.0 Hz, 1H), 3.64 (m, 2H),

3.60 – 3.53 (m, 1H), 3.10 (q, J = 7.3 Hz, 1H), 3.04 (dt, J = 13.1, 7.7 Hz, 2H), 1.79 – 1.61 (m, 6H), 1.47 (p, J = 7.5 Hz, 3H), 1.29 (t, J = 7.3 Hz, 1H); ^{13}C NMR (126 MHz, D_2O) δ 169.1, 102.2, 100.7, 98.0, 78.9, 78.6, 73.3, 73.2, 72.8, 70.3, 70.2, 69.9, 67.5, 67.46, 67.1, 66.9, 66.8, 61.1, 61.1, 60.9, 59.7, 59.4, 46.9, 42.7, 39.3, 29.8, 27.9, 26.5, 25.3, 24.9, 22.5, 22.4, 10.5; HRMS (TOF ESI$^-$) Calcd for $C_{23}H_{44}NO_{16}$ [M+Na]$^+$ 612.2480, found 612.2487.

ELSD trace **16:** RT: 2.779 min, gradient 10 to 40% over 10 min ACN/H_2O 0.1% TFA

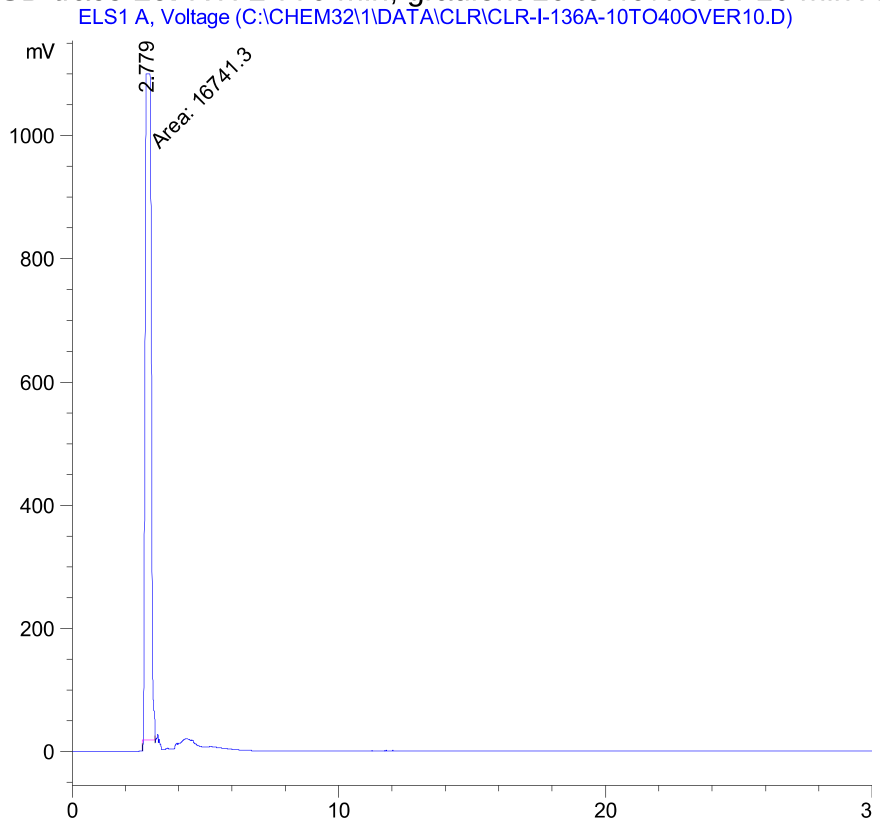

Di-*tert*-butyl (*S*)-4-(2-((((9*H*-fluoren-9-yl)methoxy)carbonyl)amino)-6-((diphenyl(*p*-tolyl)methyl)amino)hexanamido)-4-(3-(*tert*-butoxy)-3-oxopropyl)heptanedioate (19)

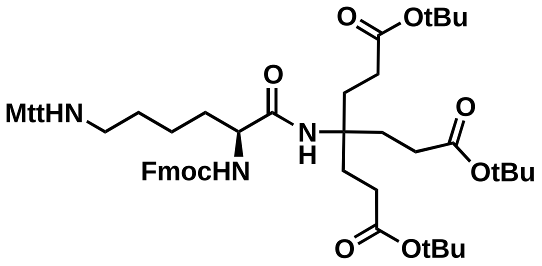

To a solution stirring solution of amino triester **18** (0.5 g, 1.2 mmol), N^{α}-Fmoc-N^{ε}-(4-methyltrityl)-L-lysine **17** (1.5 g, 2.4 mmol) and

HATU (1.4 g, 3.6 mmol) in DMF (10 mL) was added Hünig's base (0.9 mL, 5 mmol). The reaction was allowed to stir for 24 h, then concentrated under reduced pressure. The lysine-dendrimer conjugate was purified by column chromatography [hexanes/EtOAc 2:1] to afford an iridescent foamy white powder **19** (1.048 g, 1.03 mmol, 85%).

19: R_f = 0.63 [hexanes:EtOAc (2:1) (CAM & Ninhydrin)]; mp 62-65 °C ($CHCl_3$); ^{1}H NMR (500 MHz, $CDCl_3$) δ 7.75 – 7.66 (m, 2H), 7.57 (d, J = 7.5 Hz, 2H), 7.47 – 7.43 (m, 4H), 7.33 (d, J = 8.0 Hz, 4H), 7.30 – 7.20 (m, 6H), 7.18 – 7.10 (m, 2H), 7.05 (dd, J = 8.2, 4.1 Hz, 2H), 6.46 (s, 1H), 5.30 (t, J = 7.5 Hz, 1H), 4.38 (qd, J = 13.3, 12.0, 6.0 Hz, 3H), 4.20 (t, J = 7.0 Hz, 1H), 4.02 (bs, 1H), 3.71 (s, 1H), 2.28 (d, J = 4.0 Hz, 4H), 2.25 – 2.17 (m, 4H), 2.11 (d, J = 7.7 Hz, 2H), 1.96 (t, J = 7.7 Hz, 4H), 1.81 – 1.74 (m, 1H), 1.60 (bs, 1H), 1.53 – 1.48 (m, 3H), 1.40 (s, 27H), 1.34 (d, J = 6.6 Hz, 2H); ^{13}C NMR (126 MHz, $CDCl_3$) δ 173.0, 172.8, 170.9, 156.1, 155.8, 146.4, 146.3, 143.9, 143.7, 143.3, 143.2, 141.3, 141.2, 135.6, 135.57, 128.6, 128.5, 128.49, 128.4, 128.0, 127.7, 127.0, 127.02, 126.1, 126.07, 125.1, 125.0, 119.9, 119.9, 119.89, 80.7, 77.3, 70.6, 70.57, 66.94, 66.90, 57.6, 55.3, 53.8, 53.4, 52.3, 47.2, 43.4, 43.2, 33.0, 32.6, 30.7, 30.4, 29.9, 29.7, 28.2, 28.0, 27.9, 23.3, 23.0, 20.9; HRMS (TOF ESI) Calcd for $C_{63}H_{80}N_3O_9$ $[M+H]^+$ 1022.5895, found 1022.5897.

ELSD trace **19:** RT: 13.41 min, gradient 50 to 90% over 10 min ACN/H_2O 0.1% TFA

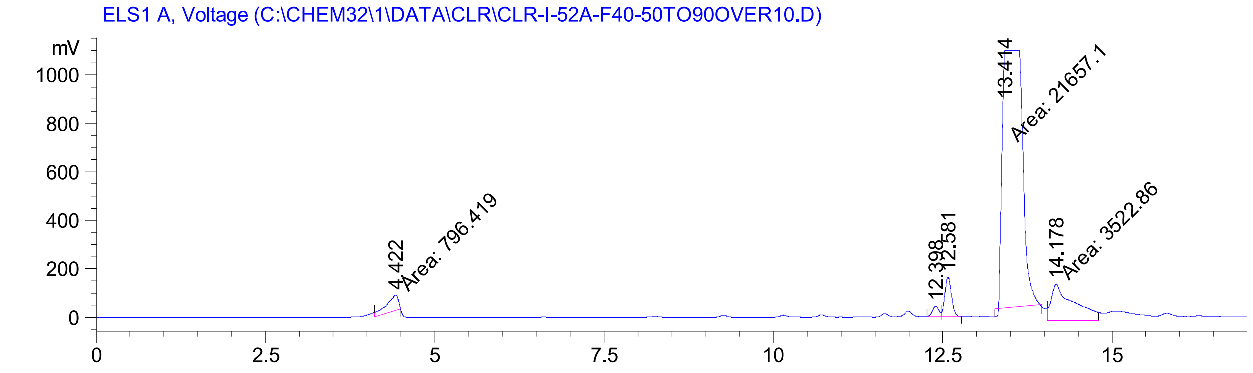

280 nm trace **19**

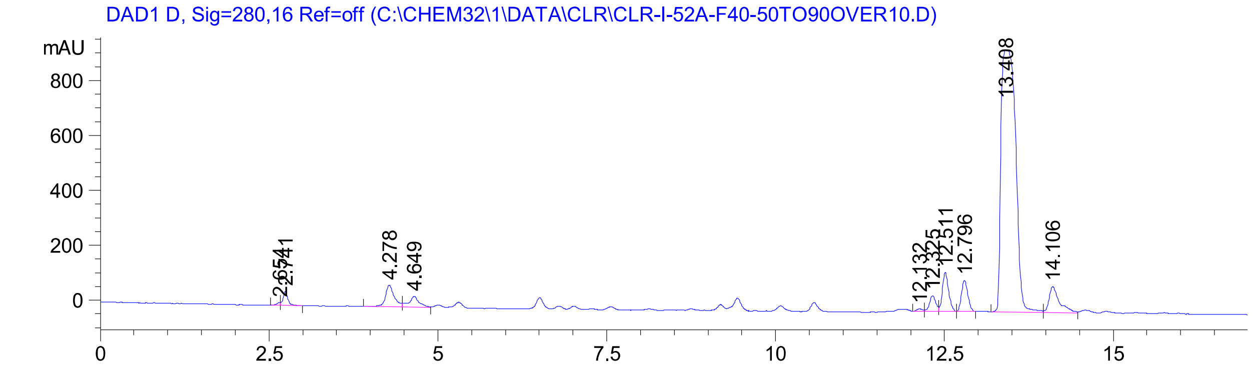

Di-*tert*-butyl (*S*)-4-(2-(6-((((9*H*-fluoren-9-yl)methoxy)carbonyl)amino)hexanamido)-6-((diphenyl(*p*-tolyl)methyl)amino)hexanamido)-4-(3-(*tert*-butoxy)-3-oxopropyl)heptanedioate (22)

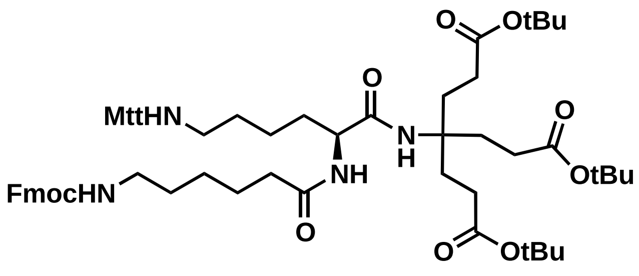

To a stirring solution of N^{α}-Fmoc-protected lys-dendrimer **19** (1.7 g, 1.7 mmol) in CH_3CN (25 mL) was added Et_2NH (1.72 mL, 16.6 mmol). The reaction was left to stir for 2 h, then concentrated under reduced pressure with toluene azeotrope to afford **20** as a white solid. The free amine was used without further purification.

20: R_f = 0.5 [hexanes:EtOAc (1:1)) (CAM & Ninhydrin)]; HRMS (TOF ESI) Calcd for

$C_{48}H_{69}N_3O_7$ $[M+Na]^+$ 822.5033, found 822.4993.

ELSD trace **20:** RT: 8.26 min**,** gradient 50 to 90% over 10 min ACN/H_2O 0.1% TFA

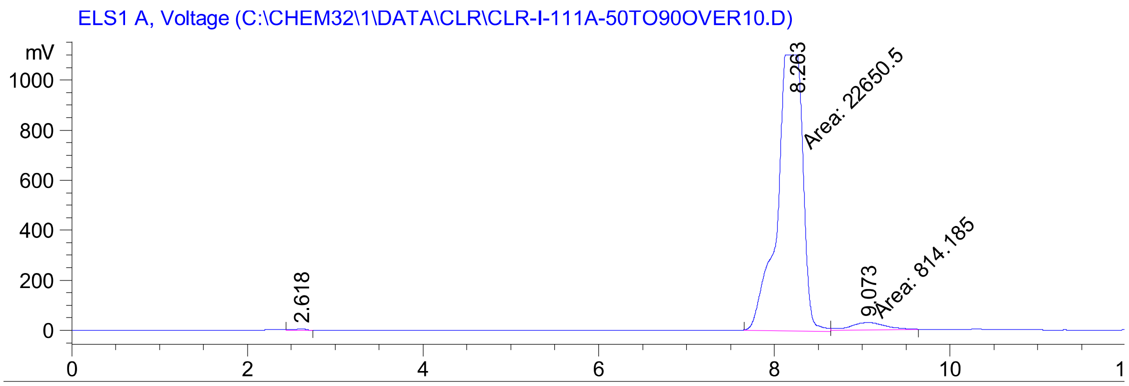

254 nm trace **20**

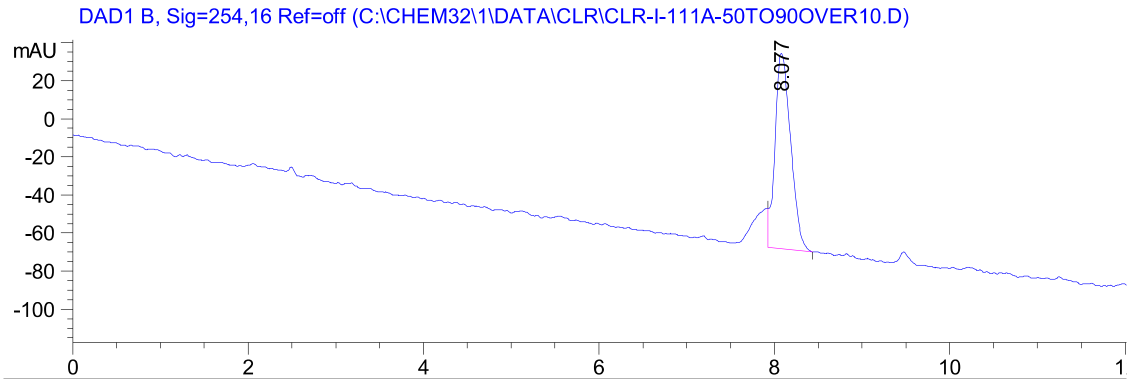

To a solution of free amine **20** (1.33 g, 1.66 mmol), N^{ε}-Fmoc-aminohexanoic acid **21** (1.20 g, 3.39 mmol) and HATU (1.90 g, 4.99 mmol) in DMF (15 mL) was added Hünig's base (1.2 mL, 6.7 mmol). The reaction was left to stir for 24 h, then concentrated under reduced pressure to remove DMF. The crude mixture was then partitioned between EtOAc (100 mL) and H_2O (100 mL), separated, then extracted with more EtOAc (2 × 100 mL). The collected organic layers were washed with 10% aqueous $CuSO_4$ (100 mL) and brine (100 mL), dried over Na_2SO_4, then concentrated under reduced pressure. The conjugated product was purified by column chromatography [gradient hexanes/EtOAc 1:0 to 1:1] to afford a white solid (1.8 g, 1.6 mmol, 95% over two steps).

22: R_f = 0.68 [hexanes:EtOAc (1:1) (CAM & Ninhydrin)]; ^{1}H NMR (500 MHz, CD_3CN) δ 7.79 (d, *J* = 7.6 Hz, 2H), 7.61 (d, *J* = 7.5 Hz, 2H), 7.55 (s, 1H), 7.43 – 7.34 (m, 5H), 7.33 – 7.25 (m, 4H), 7.22 (t, *J* = 7.7 Hz, 4H), 7.13 (t, *J* = 7.3 Hz, 2H), 7.05 (d, *J* = 9.1 Hz, 2H), 6.51 (d, *J* = 7.4 Hz, 1H), 6.16 (s, 1H), 5.68 (s, 1H), 4.26 (d, *J* = 7.0 Hz, 2H), 4.17 (t, *J* = 7.1 Hz, 1H), 4.03 (q, *J* = 7.2 Hz, 4H), 3.01 (q, *J* = 6.7 Hz, 2H), 2.23 (s, 3H), 2.19 (s, 7H), 2.11 (t, *J* = 7.4 Hz, 2H), 2.10 – 2.03 (m, 6H), 1.99 (t, *J* = 6.9 Hz, 2H), 1.91 (p, *J* = 2.5 Hz, 2H), 1.85 – 1.77 (m, 5H), 1.66 – 1.58 (m, 1H), 1.52 (q, *J* = 7.5 Hz, 2H), 1.48 – 1.37 (m, 4H), 1.35 (s, 27H), 1.32 – 1.21 (m, 4H), 1.17 (t, *J* = 7.1 Hz, 4H); ^{13}C NMR (126 MHz, CD_3CN) δ 174.2, 173.6, 172.5, 171.7, 157.4, 147.7, 145.3, 144.5, 142.2, 136.8, 129.5, 129.50, 129.3, 128.7, 128.1, 127.1, 126.17, 121.0, 80.9, 79.2, 71.5, 66.9, 61.0, 58.3, 55.0, 48.2, 44.3, 41.3, 36.5, 32.6, 31.0, 30.3, 30.3, 30.2, 28.3, 26.9, 26.0, 24.4, 21.2, 21.0, 14.5; HRMS (TOF ESI) Calcd for $C_{69}H_{90}N_4O_{10}$ $[M+Na]^+$ 1157.6555, found 1157.6550.

ELSD trace **22:** RT: 13.77 min, gradient 50 to 90% over 10 min ACN/H_2O 0.1% TFA

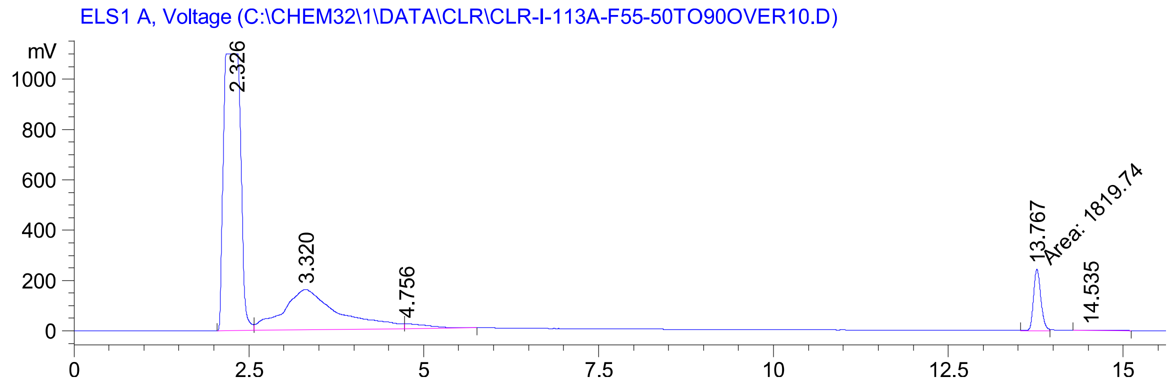

254 nm trace **22**

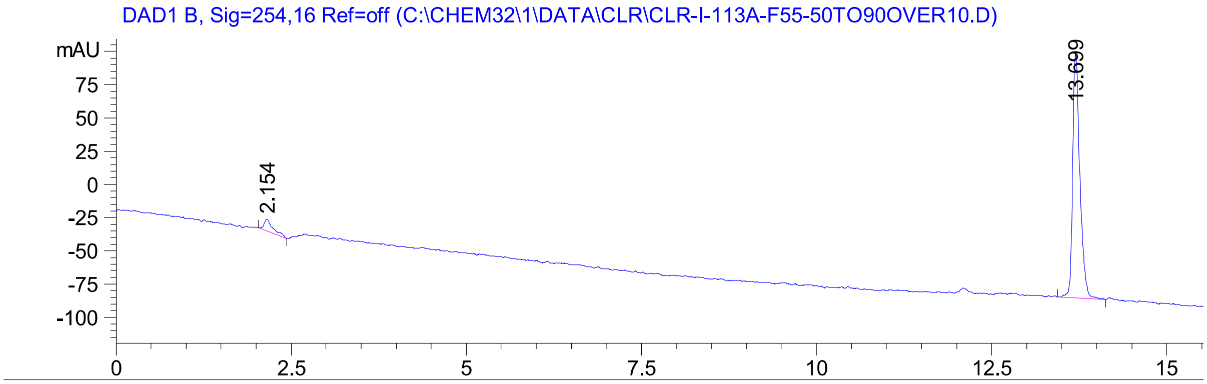

(*S*)-5-((5-(6-((((9*H*-Fluoren-9-yl)methoxy)carbonyl)amino)hexanamido)-6-((1,7-di-*tert*-butoxy-4-(3-(*tert*-butoxy)-3-oxopropyl)-1,7-dioxoheptan-4-yl)amino)-6-oxohexyl)carbamoyl)-2-(6-(dimethylamino)-3-(dimethyliminio)-3*H*-xanthen-9-yl)benzoate (24)

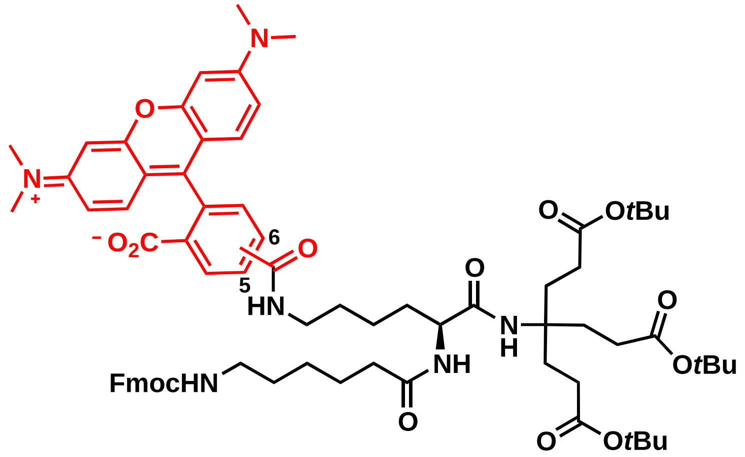

6-(Mtt) Lys-Hex(6-Fmoc)-Dendrimer **22** (1.8 g, 1.6 mmol) was dissolved in a solution of [CH_2Cl_2/TFE/AcOH 3:1:1] (25 mL). The reaction was allowed to stir for 2 h, then concentrated under reduced pressure with toluene azeotrope. The crude oil was partitioned between hexanes (100 mL) and CH_3CN (100 mL), and separated to remove trityl byproducts. The acetonitrile layer was concentrated under reduced pressure to afford an amber-colored oil, which was used without further purification (1.40 g, 1.59 mmol, 99% crude).

23: HRMS (TOF ESI) Calcd for $C_{49}H_{74}N_4O_{10}$ $[M+Na]^+$ 901.5303, found 901.5297.

ELSD trace **23:** RT=10.43 min, gradient 50 to 90% over 10 min ACN/H_2O 0.1% TFA

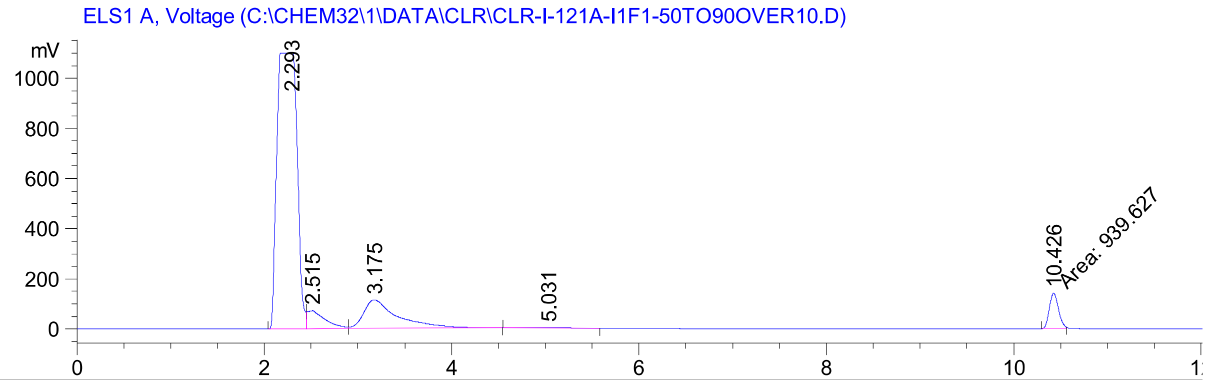

254 nm trace **23**

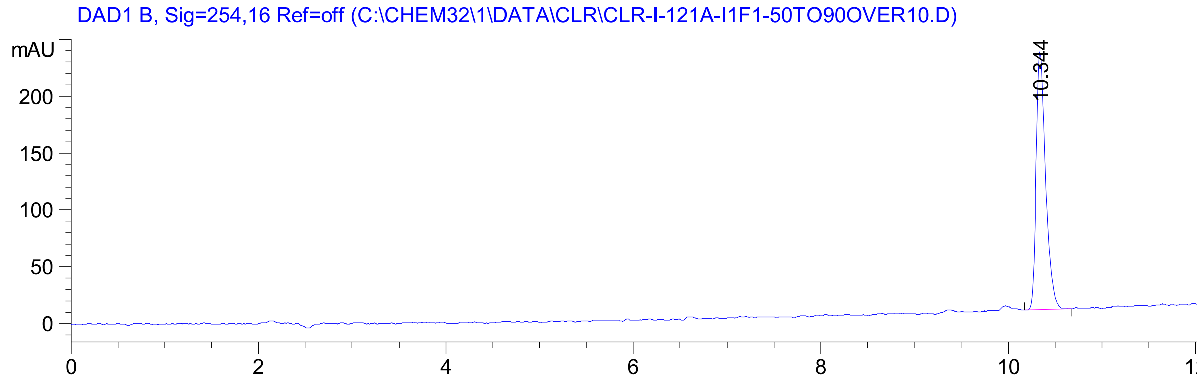

To a solution of free amine **23** (0.097 g, 0.110 mmol) in DMF (2.5 mL) was added 5(6)-TAMRA-NHS (0.064 g, 0.121 mmol) then Hünig's base (80.0 μL, 0.448 mmol). The reaction mixture was allowed to stir for 18 h until no free amine was observed by TLC (Ninhydrin). The reaction mixture was concentrated under reduced pressure then purified by preparative reverse-phase HPLC (10–90% MeCN/H_2O over 10 minutes, RT= 16.4 min) to afford a deep magenta powder as a regioisomeric mixture of 5(6)-TAMRA (0.128 g, 0.099 mmol, 90%).

24: R_f = 0.75 [CH_2Cl_2:MeOH (4:1) (Ninhydrin)]; *N*-rotomers, major TAMRA isomer: ^1H NMR (500 MHz, CD_3CN) δ 9.27 (s, 1H), 8.79 (dd, *J* = 7.9, 1.8 Hz, 1H), 8.34 (d, *J* = 7.2 Hz, 4H), 8.12 (d, *J* = 7.4 Hz, 3H), 7.93 (t, *J* = 7.8 Hz, 4H), 7.82 (td, *J* = 7.4, 1.1 Hz, 3H), 7.61 – 7.54 (m, 3H), 7.53 (d, *J* = 7.3 Hz, 1H), 7.41 (t, *J* = 2.3 Hz, 1H), 7.39 (t, *J* = 2.3 Hz, 1H), 7.34 – 7.26 (m, 4H), 7.22 (bs, 4H), 6.98 (s, 1H), 6.44 (m, 1H), 4.77 (m, 3H), 4.65 (t, *J* = 7.1 Hz, 1H), 3.99 (qq, *J* = 12.8, 6.4 Hz, 2H), 3.90 (q, *J* = 6.2 Hz, 1H), 3.75 (m, 12H), 3.58 (m, 2H), 2.74 (d, *J* = 7.6 Hz, 2H), 2.72 – 2.62 (m, 8H), 2.50 (p, *J* = 2.5 Hz, 2H), 2.47 (s, 2H), 2.46 – 2.41 (m, 6H), 2.40 – 2.35 (m, 3H), 2.27 – 2.17 (m, 4H), 2.16 – 2.03 (m, 4H), 1.95 (s, 27H), 1.88 – 1.78 (m, 4H); ^{13}C NMR (126 MHz, CD_3CN) δ 174.8, 173.6, 173.5, 172.7, 172.6, 166.7, 166.6, 166.5, 160.7, 160.4, 160.0, 158.3, 158.3, 157.5, 145.2, 142.1, 139.4, 137.5, 137.4, 135.0, 132.6, 132.3, 132.0, 131.8, 131.7, 131.5, 131.0, 130.0, 129.6, 128.7, 128.1, 126.1, 121.0, 115.3, 114.3, 97.2, 80.9, 80.9, 67.0, 66.9, 58.5, 58.4, 54.9, 54.8, 48.1, 41.4, 41.3, 40.4, 40.2, 38.5, 36.5, 36.4, 32.1, 30.3, 30.26, 30.2, 29.6, 29.5, 28.3, 27.0, 26.9, 26.2, 23.7, 22.3, 17.7; HRMS (TOF ESI^+) Calcd for $C_{74}H_{95}N_6O_{14}$ $[M+H]^+$ 1291.6906, found 1291.6906.

ELSD trace **24**: RT=16.35 min, gradient 10 to 90% over 15 min ACN/H_2O with 0.1% TFA

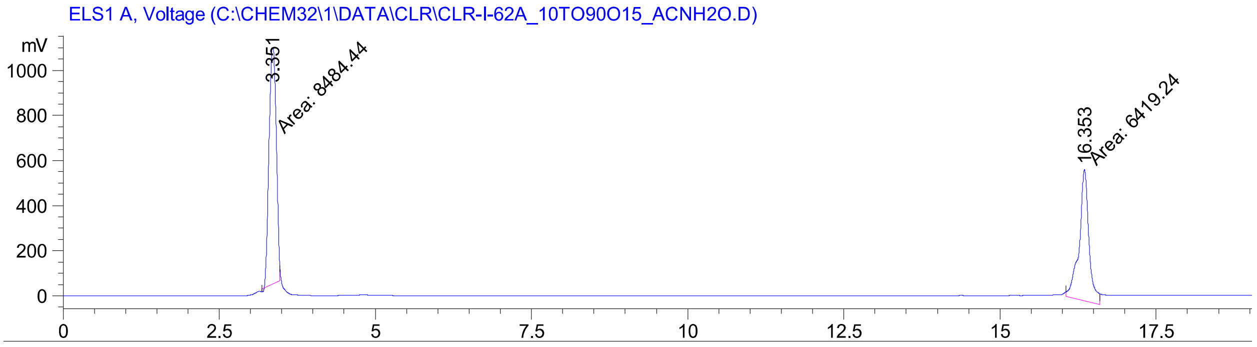

555 nm trace **24**

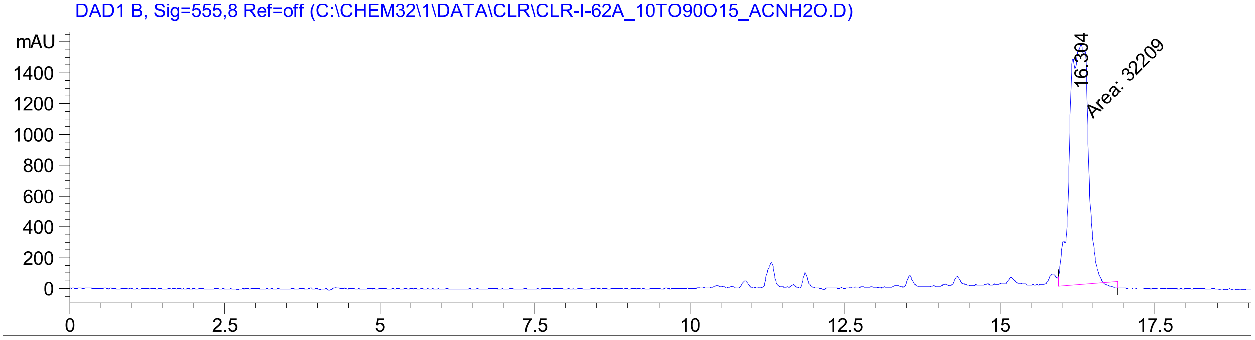

(*S*)-5-((5-(6-((((9*H*-Fluoren-9-yl)methoxy)carbonyl)amino)hexanamido)-6-((1,7-bis ((5-(α-D-mannopyranosyl-(1→2)-α-D-mannopyranosyl-(1→2)-α-D-mannopyranoside))hydroxypentyl)amino)-4-(3-((5-(α-D-mannopyranosyl-(1→2)-α-D-mannopyranosyl-(1→2)-α-D-mannopyranoside)hydroxypentyl)amino)-3-oxopropyl)-1,7-dioxoheptan-4-yl)amino)-6-oxohexyl)carbamoyl)-2-(6-(dimethylamino)-3-(dimethyliminio)-3*H*-xanthen-9-yl)benzoate (27)

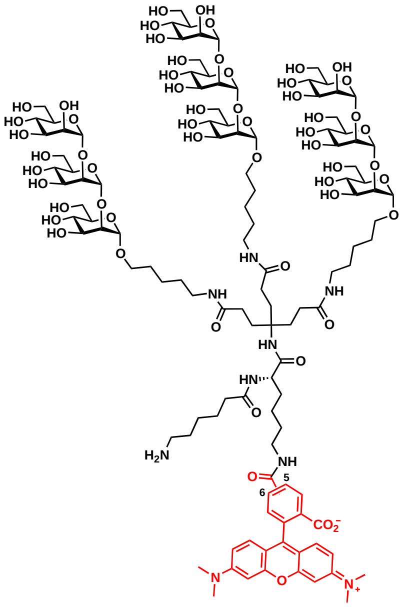

Triester **24** (0.112 g, 0.864 mmol) was suspended in a solution of [TFA/THF/H_2O 90:8:2] (5 mL) and left to stir for 2 h. The reaction mixture was concentrated under reduced pressure with toluene azeotrope. The free acid **25** was purified by preparative reverse-phase HPLC (10–90% MeCN/H_2O over 15 min, RT=11.9 min) to afford a deep magenta powder as a regioisomeric mixture (0.08 g, mmol, 83%).

25: R_f=0.45 [CH_2Cl_2:MeOH (4:1) (Bromocresol green)]; HRMS (ESI$^+$ LCMS) Calcd for $C_{62}H_{71}N_6O_{14}$ $[M+H]^+$ 1123.5028, found 1123.5038.

ELSD trace **25**: RT=11.87 min, gradient 10 to 90% over 15 min ACN/H_2O with 0.1% TFA

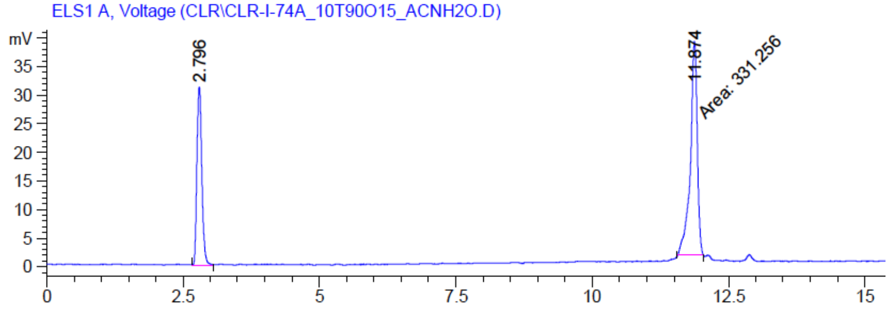

555 nm trace **25**

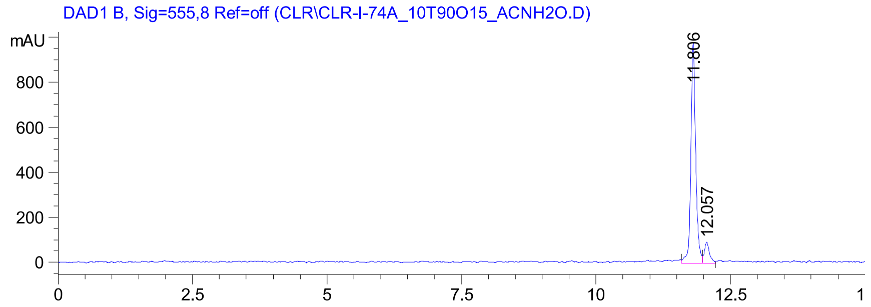

To a stirring solution of free acid **25** (15 mg, 0.01 mmol) in DMF (5 mL) was added a solution of CDI (5 mg, 0.04 mmol) in DMF (0.6 mL). The acyl imidazole activation was monitored by LCMS until full activation was complete after 5 h. To the activated acyl imidazole was added trimannose **16** (40 mg, 0.07 mmol) in DMF (1 mL). The reaction mixture was then allowed to stir for 12 h, then concentrated under reduced pressure.

To protected glycodendrimer **26** (30 mg, 0.011 mmol) in DMF (0.5 mL) was added Et_2NH (1.0 mL, 9.67 mmol) and left to stir for 15 min (note: the addition of Et_2NH will turn off the fluorophore, which is observed by the solution fading from deep magenta to pale pink). The reaction mixture was concentrated to remove residual Et_2NH. The solution returned to a deep magenta purple upon removal. Residual dibenzylfulvene by-products were removed by the addition of pentanes (1 mL) to the solution of DMF. The pentanes were removed by pipet, then the product solution was freeze dried. The deprotected glycodendrimer was then purified by preparative reverse-phase HPLC (10-90% MeCN:H_2O over 10 min, RT=9.45 min) to afford a deep magenta oil as a regioisomeric mixture of **27** (27 mg, 0.01 mmol, 90%).

27: ^{1}H NMR (600 MHz, D_2O) δ 8.46 (s, 1H), 8.16 – 7.96 (m, 5H), 7.93 (s, 4H), 7.50 – 7.08 (m, 12H), 5.30 (s, 3H), 5.10 (d, *J* = 6.7 Hz, 5H), 5.05 (s, 3H), 5.03 (s, 1H), 4.11 (s, 3H), 4.09 – 4.06 (m, 5H), 3.98 – 3.82 (m, 32H), 3.82 – 3.59 (m, 40H), 3.55 (d, *J* = 7.3 Hz, 7H), 3.28 – 3.13 (m, 5H), 3.11 (d, *J* = 3.0 Hz, 8H), 3.08 (qd, *J* = 7.3, 2.3 Hz, 60H), 3.03 – 2.99 (m, 21H), 2.88 (d, *J* = 2.2 Hz, 4H), 2.86 (d, *J* = 2.6 Hz, 11H), 1.74 – 1.61 (m, 24H), 1.51 – 1.41 (m, 13H), 1.28 (td, *J* = 7.3, 2.0 Hz, 85H); HRMS (TOF ESI) Calcd for $C_{116}H_{183}N_9O_{57}$ $[M+H]^+$ 2615.1776, found 2615.2188.

Trimannose-linked bioerodible microparticles (2)

A polyanhydride copolymer composed of 20:80 CPH/SA was synthesized via melt polycondensation as previously described.[12] The resulting polymer was dissolved at 10 mg/mL in methylene chloride. The polymer solution was spray dried using a mini spray dryer (B-290, Büchi, Switzerland). The resulting particle morphology

and size were characterized with scanning electron microscopy (FEI Quanta 250, FEI, Hillsboro, OR). ImageJ (NIH) analysis determined particle size was ~1 µm.

The particles were surface functionalized with trimannose glycodendrimer **27** by adding 10 equivalents (equiv) EDC, 12 eq. NHS, and 2 equiv of trimannose to 30 mg of microparticles suspended 5 mg/mL in nanopure water. The mixture was incubated while rotating at 4 °C for 1 h before centrifuging (10,000 rpm, 10 min). The supernatant was removed and replaced with fresh water before centrifuging, repeating the wash step three times. The final microparticles were dried for 1 h under vacuum.

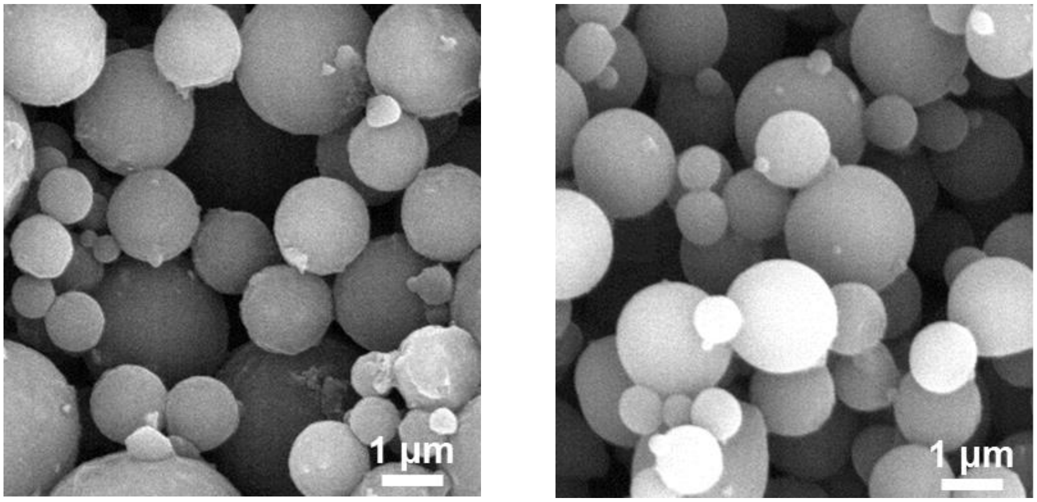

Figure S1: Scanning electron micrographs of glycodendrimer-linked particles (left) and blank/non-linked particles (right)

Trimannose-coated latex beads (1)

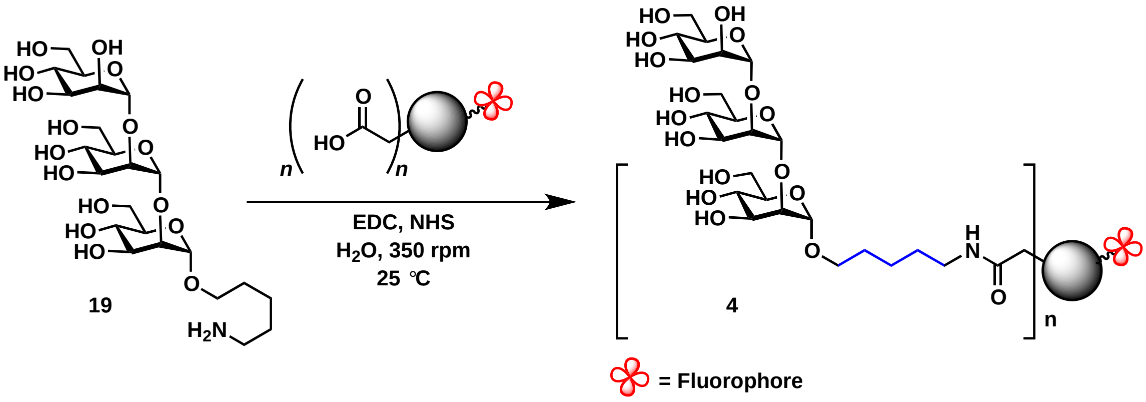

To a solution of FluoSpheres® (1 mL, $3.64*10^{10}$ beads, $3.5*10^{-4}$ mmol) in MES buffer (200 μL) was added a solution of EDCI (8 mg , 0.04 mmol) in ultra pure H_2O (0.8 mL) and NHS (5 mg, 0.04 mmol) in ultra pure H_2O (0.5 mL) and trimannose **16** (50 mg, 0.08 mmol) in ultra pure H_2O (0.5 mL). The reaction mixture was left to stir for 24 h at 25 °C, then centrifuged (10,000 rpm) for 30 s. The supernatant was decanted then the beads were re-suspended in ultrapure H_2O (5 mL). This was again centrifuged (10,000 rpm) for 30 s, and repeated twice more to remove EDCI, NHS and remaining trimannose. The beads were then characterized by the phenol sulfuric acid assay to measure trimannose surface density (35 mg trimannose/g bead).

Phenol-Sulfuric assay of trimannose coated-FluoSpheres® [13]

To a solution of unknown concentration of trimannose-coated FluoSpheres® in H_2O (500 μL) in a centrifuge tube (1.5 mL total volume) was added 80% phenol in H_2O (100 μL) then concentrated H_2SO_4 (400 μL) paying special attention to add the H_2SO_4 to the solution surface to ensure optimal mixing. The solution was then vortexed for 10 s, then incubated at 90 °C for 5 min and 25 °C for 5 min. The solution was transferred to a 1 mL cuvette then absorbance was measured at 487 nm (ultra pure H_2O used as blank). The procedure was replicated and absorbance measured at 487 nm in triplicate. This procedure was replicated in triplicate with the uncoated FluoSpheres and unconjugated Trimannose **19**. The number of beads and amount of trimannose on the trimannose-coated FluoSpheres® were extrapolated from D-mannose and fluorosphere standard curves and the following FluoSpheres formula (Equation S1):

$$\#\ \text{of Microspheres per mL} = \frac{6C\ x\ 10^{12}}{\rho\ x\ \pi\ x\ \phi^3} \qquad \text{(S1)}$$

Here: C = concentration of suspended beads in g/mL (0.02 g/mL for a 2% suspension)

ϕ = diameter of microspheres in μm (1 μm for product # F8821)

ρ = density of polymer in g/mL (1.05 for polystyrene)

Charge density of microspheres: 0.0175 meq/g

In vivo experiments

Animals

47 week male and female C57BL/6 wild type mice (WT) were purchased from Jackson Laboratories (Bar Harbor, ME) and maintained at the University of Iowa Animal Care under specific pathogen-free standard housing. C57BL/6 mannose receptor knockout ($MR^{-/-}$) mice[14] were obtained from the University of Notre Dame as a gracious gift from Jeffery Schorey, bred in-house under standard housing. 34 - 47 week old male and female $MR^{-/-}$ mice used in the experiments were maintained under standard housing. The University of Iowa Institutional Animal Care and Use Committee approved all animal procedures.

Parasite culture and *in vivo* infection

Leishmania major (MHOM/IL/80/Friedlin) cultures were maintained in Complete Grace's Insect medium (Sigma) with 10% heat inactivated-fetal bovine serum, 2 mM glutamine, 100 U/mL penicillin and 100 µL/mL of streptomycin at 28 °C and harvested for parasite freeze thaw antigen or live parasites for inoculation as described previously.[15,16] The

left hind footpad was injected as follows based on studies in Grinnage-Pulley et al. [17]: 5×10^6 *L. major* promastigotes in 50 µL of sterile PBS; 5×10^7 trimannose microparticles in 50 µL of PBS; or a single injection of 5×10^6 *L. major* promastigotes and 5×10^7 trimannose microparticles in 50 µL of PBS. n = 6–10 mice per group. Footpad thickness was measured bilaterally once a week with a dial micrometer. Footpad lesion size was calculated as the difference between the thickness of the left and right footpad. After footpad measurement, mice were humanely euthanized on day 42.

Experimental References

1. Zhang, W.; Curran, D.P. *Tetrahedron* **2006**, *62*, 11837–11865.
2. Zong, C.; Venot, A.; Dhamale, O.; Boons, G.-J. *Org. Lett.* **2013**, *15*, 342–345.
3. Patel, M.K.; Vijayakrishnan, B.; Koeppe, J.R.; Chalker, J.M.; Doores, K.J.; Davis, B.G. *Chem. Commun. (Camb).* **2010**, *46*, 9119–9121.
4. Chao, C.-S.; Lin, C.-Y.; Mulani, S.; Hung, W.-C.; Mong, K.T. *Chem. - A Eur. J.* **2011**, *17*, 12193–12202.
5. Mazurek, M.; Perlin, A.S. *Can. J. Chem.* **1965**, *43*, 1918–1923.
6. Bensoussan, C.; Rival, N.; Hanquet, G.; Colobert, F.; Reymond, S.; Cossy, J. *Tetrahedron* **2013**, *69*, 7759–7770.
7. Backinowsky, L. V; Byramova, N.E.; Tsvetkov, Y.E.; Betaneli, V.I. *Carbohydr. Res.* **1981**, *98*, 181–193.
8. Boren, H.B.; Ekborg, G.; Eklind, K.; Garegg, P.J.; Pilotti, A.; Swahn, C.-G. *Acta Chem. Scand.* **1973**, *27*, 2639–2644.

9. Yamazaki, F.; Sato, S.; Nukada, T.; Ito, Y.; Ogawa, T. *Carbohydr. Res.* **1990**, *201*, 31–50.

10. Gannedi, V.; Ali, A.; Singh, P.P.; Vishwakarma, R.A. *Tetrahedron Lett.* **2014**, *55*, 2945–2947.

11. Jaipuri, F.A.; Pohl, N.L. *Org. Biomol. Chem.* **2008**, *6*, 2686–2691.

12. Kipper, M.J.; Wilson, J.H.; Wannemuehler, M.J.; Narasimhan, B. *J. Biomed. Mater. Res. - Part A* **2006**, *76*, 798–810.

13. Dubois, M.; Gilles, K.A.; Hamilton, J.K.; Rebers, P.A.; Smith, F. *Anal. Chem.* **1956**, *28*, 350–356.

14. Akilov, O.E.; Kasuboski, R.E.; Carter, C.R.; McDowell, M.A. *J. Leukoc. Biol.* **2007**, *81*, 1188–1196.

15. Esch, K.J.; Juelsgaard, R.; Martinez, P.A.; Jones, D.E.; Christine, A. *J Immunol.* **2013**, *191*, 5542–5550.

16. Boggiatto, P.M.; Ramer-Tait, A.E.; Metz, K.; Kramer, E.E.; Gibson-Corley, K.; Mullin, K.; Hostetter, J.M.; Gallup, J.M.; Jones, D.E.; Petersen, C.A. *Clin. Vaccine Immunol.* **2010**, *17*, 267–273.

17. Grinnage-Pulley, T.L.; Kabotso, D.E.K.; Rintelmann, C.L.; Roychoudhury, R.; Schaut, R.G.; Toepp, A.J.; Gibson-Corley, K.N.; Parrish, M.; Pohl, N.L.B.; Petersen, C.A. *Infect. Immun.* **2018**, *86*, 1–13.

4-(3,3,4,4,5,5,6,6,7,7,8,8,8-Tridecafluorooctyl)benzyl benzyl(5-hydroxy pentyl) carbamate (7)

^{1}H-NMR (500 MHz, $CDCl_3$)

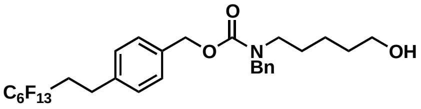

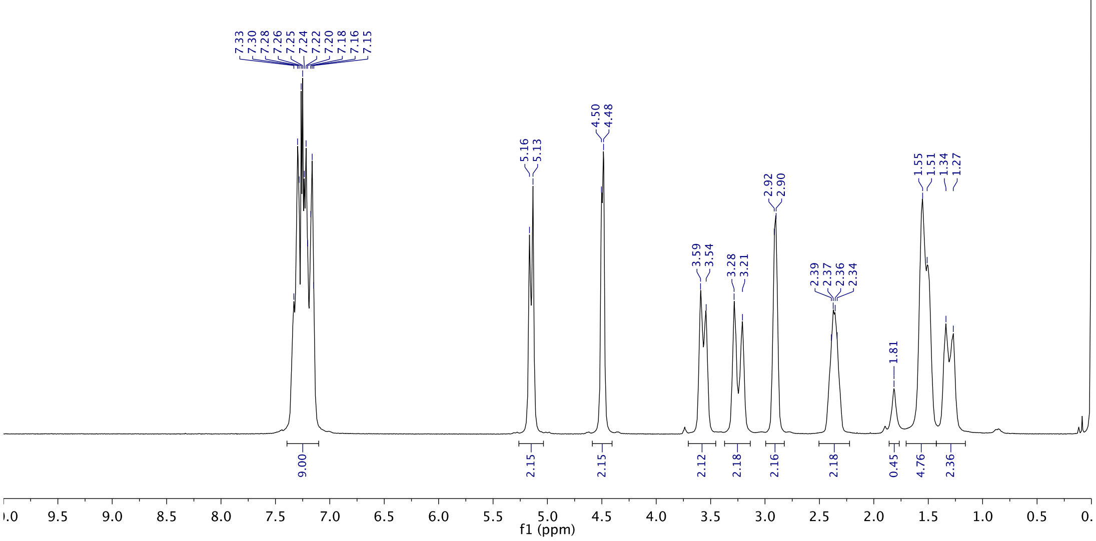

^{13}C-NMR (126 MHz, $CDCl_3$)

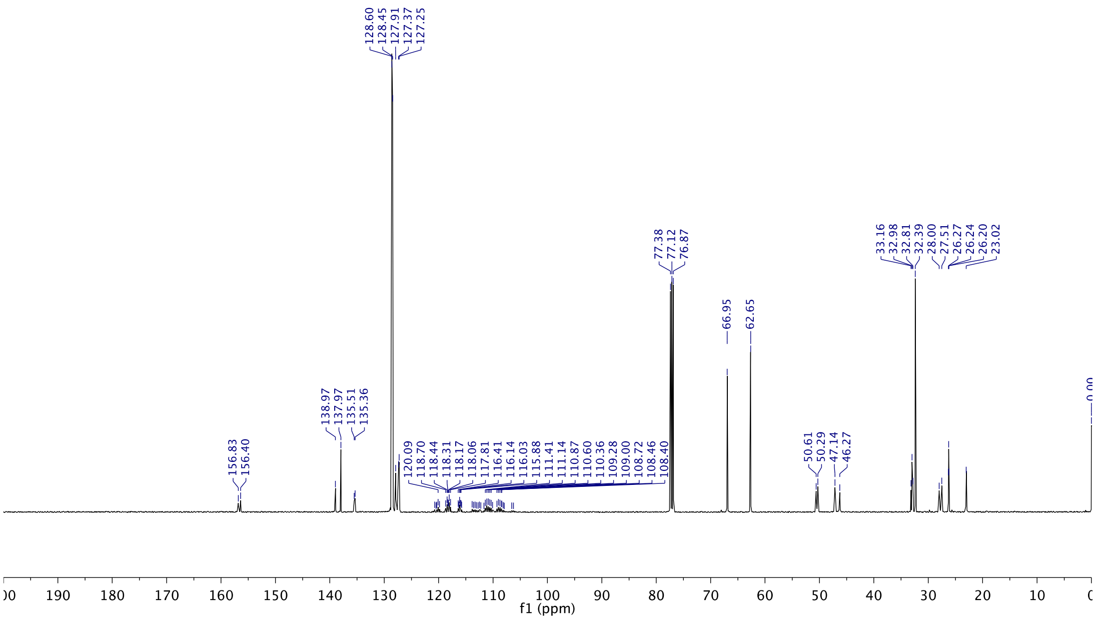

1,2,3,4,6-Penta-*O*-acetyl-α/β-D-mannopyranose (i)

^{1}H-NMR (500 MHz, $CDCl_3$)

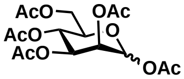

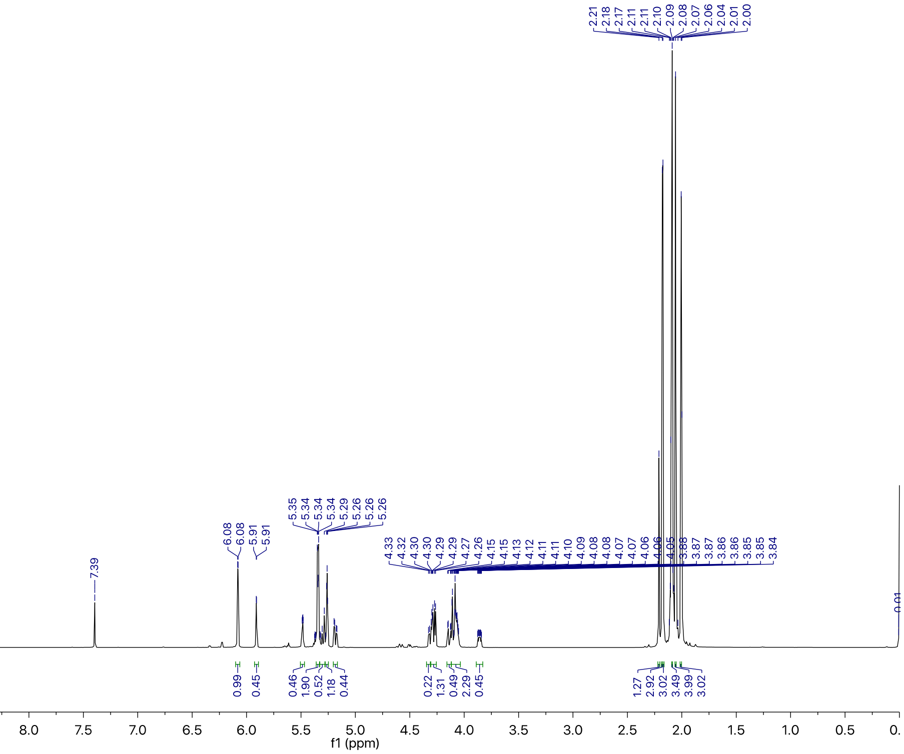

^{13}C-NMR (126 MHz, $CDCl_3$)

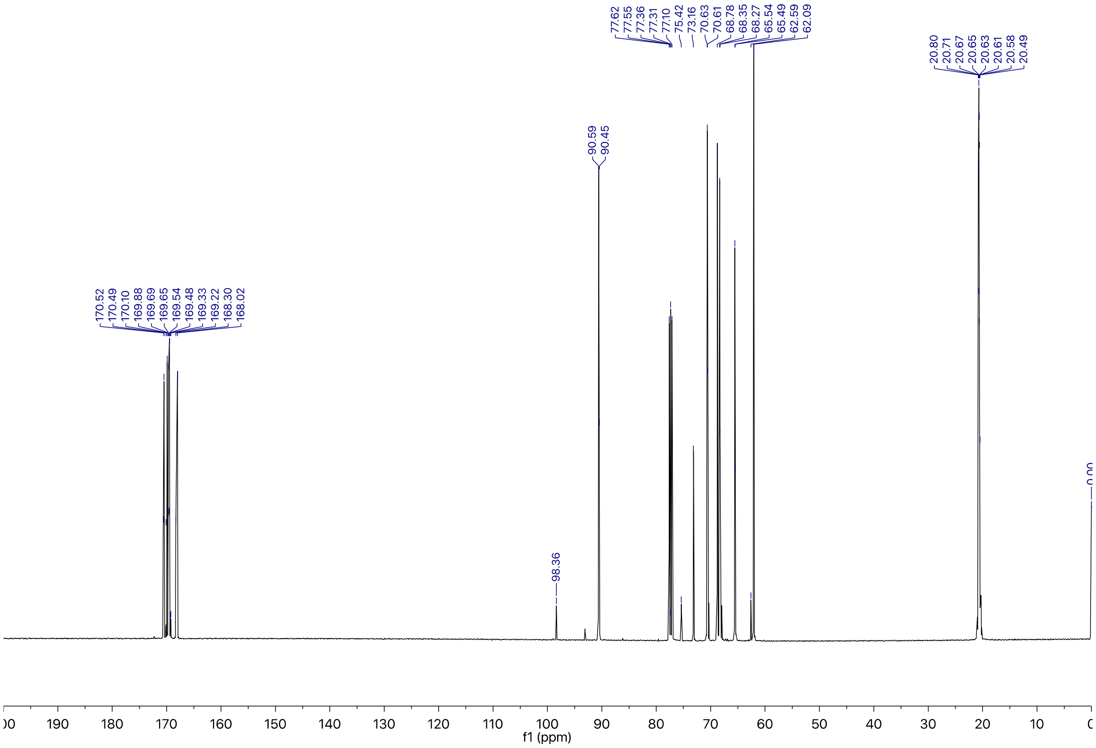

3,4,6-Tri-*O*-acetyl-1,2-*O*-[1-(*exo*- and *endo*-methoxy)ethylidene]-β-D-manno pyranose (ii)

^{1}H-NMR (600 MHz, $CDCl_3$)

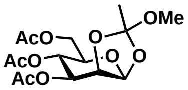

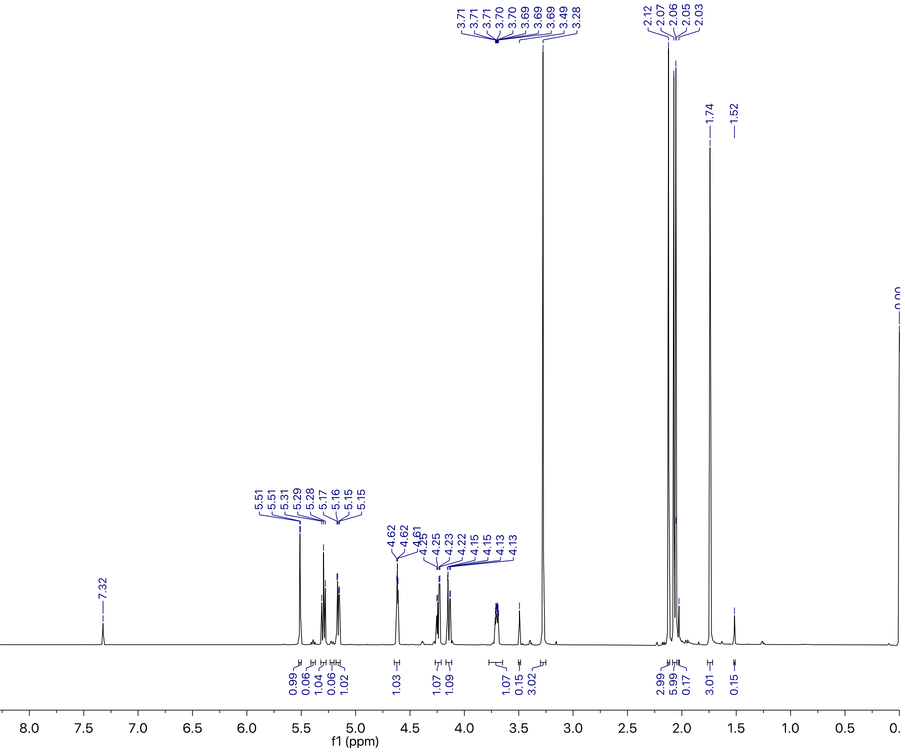

^{13}C-NMR (126 MHz, $CDCl_3$)

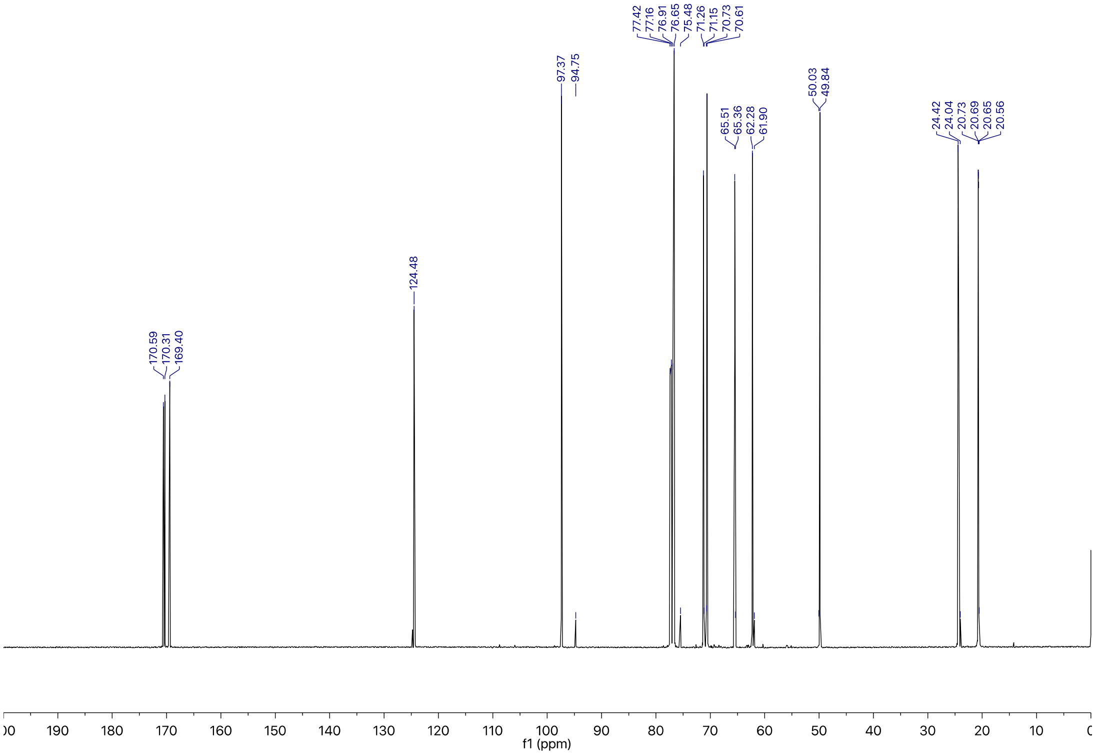

3,4,6-Tri-*O*-benzyl-1,2-*O*-[1-(*exo*- and *endo*-methoxy)ethylidene]-β-D-manno pyranose (iii)

^{1}H-NMR (500 MHz, $CDCl_3$)

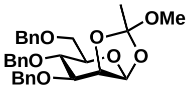

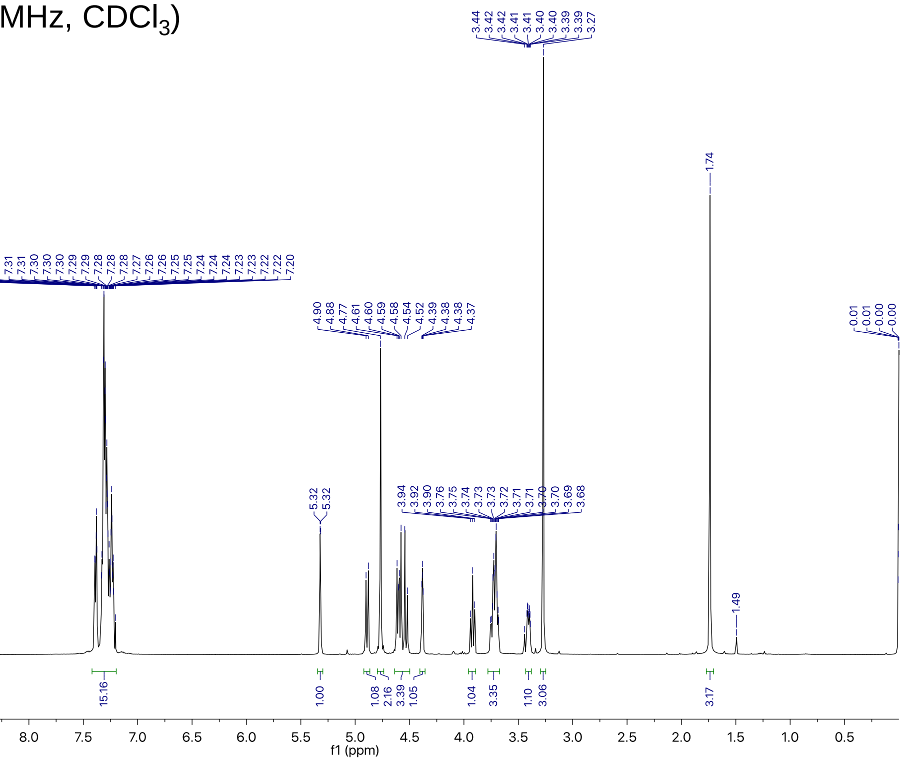

^{13}C-NMR (126 MHz, $CDCl_3$)

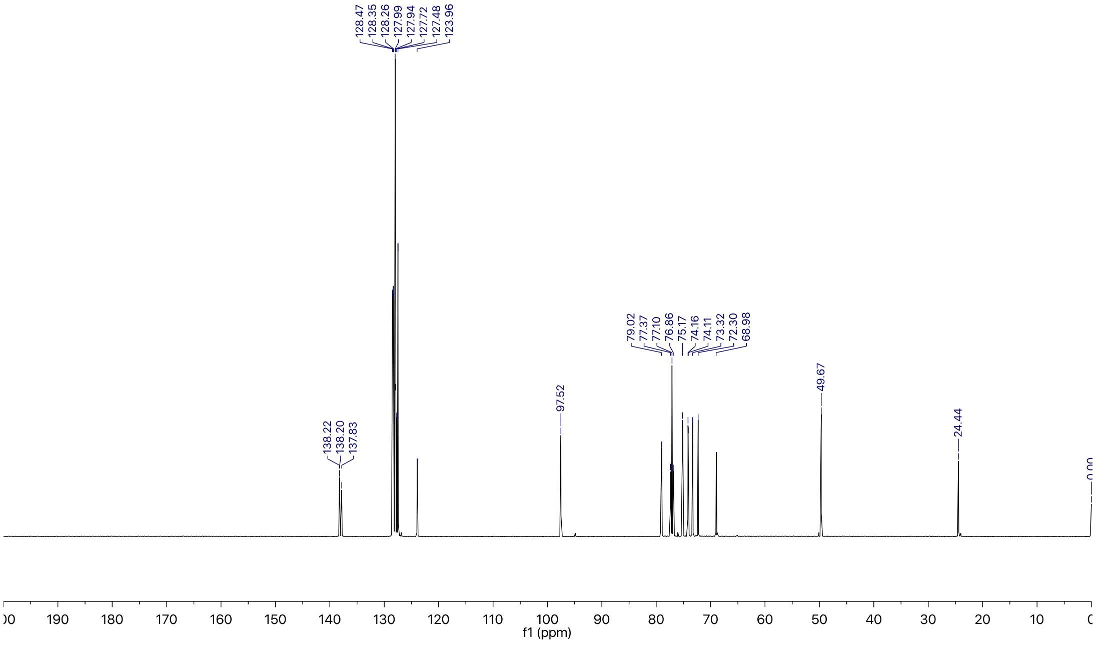

2-*O*-Acetyl-3,4,6-tri-*O*-benzyl-α/β-D-mannopyranose (iv)

^{1}H-NMR (400 MHz, $CDCl_3$)

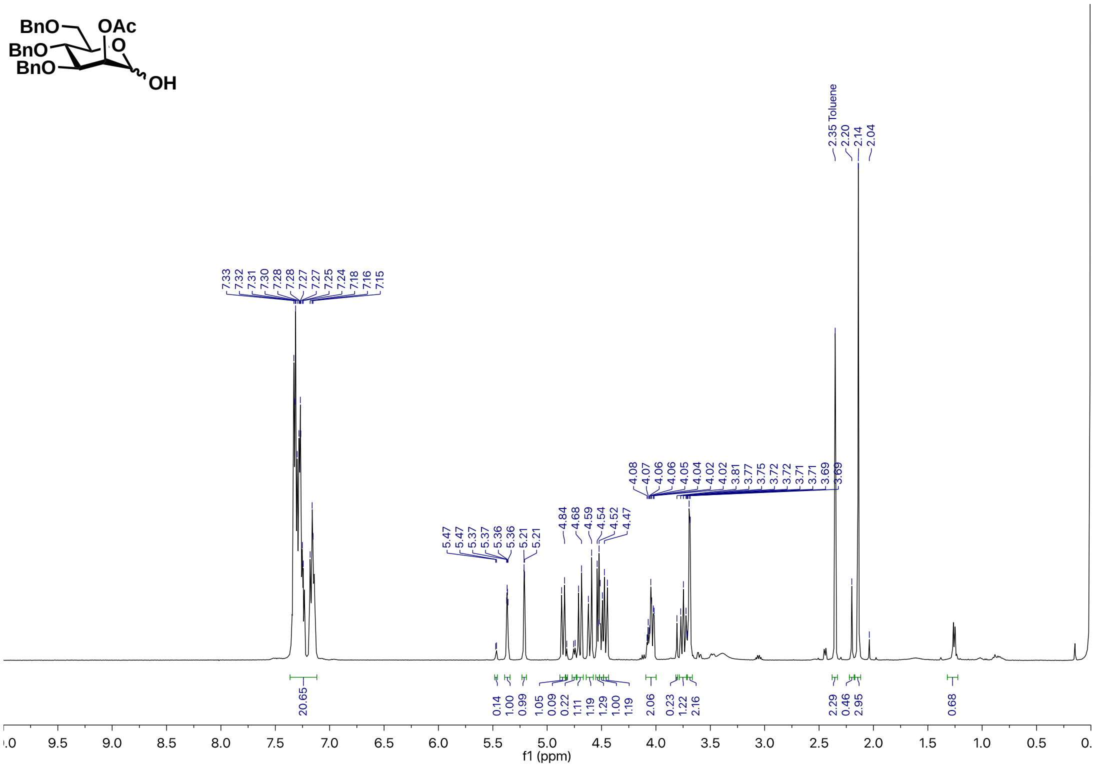

^{13}C-NMR (126 MHz, $CDCl_3$)

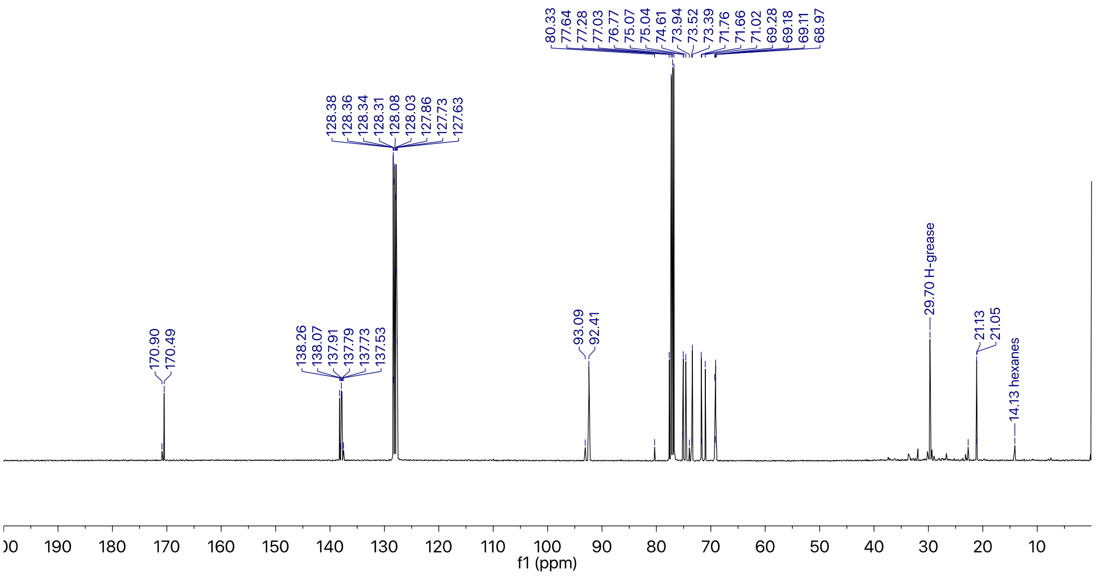

2-*O*-Acetyl-3,4,6-tri-*O*-benzyl-α-D-mannopyranosyl trichloroacetimide (3)

^{1}H-NMR (600 MHz, $CDCl_3$)

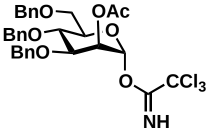

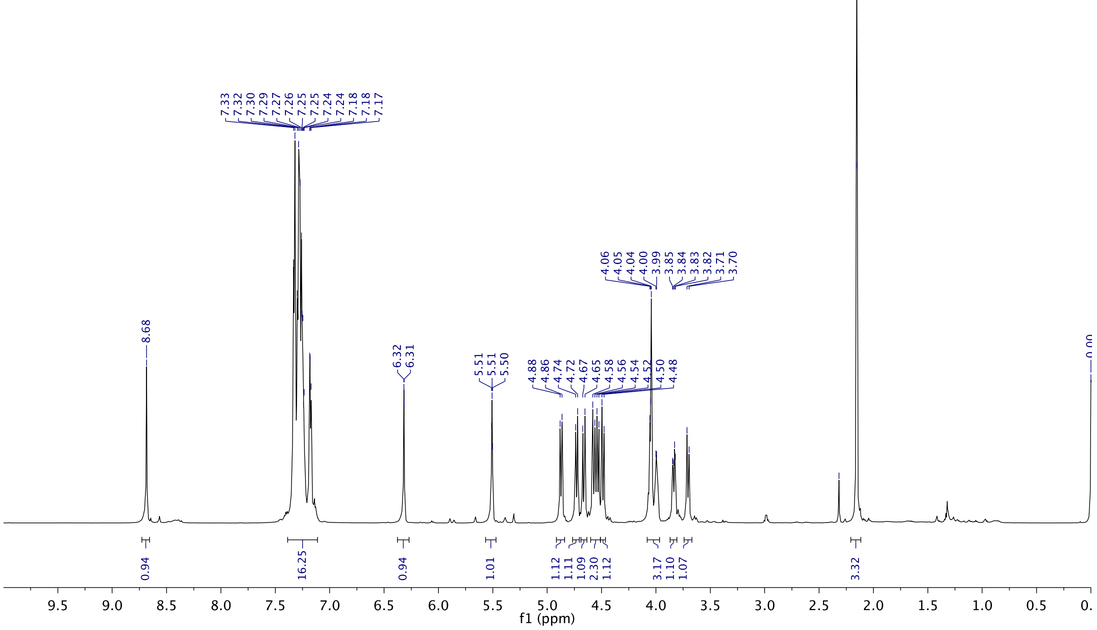

^{13}C-NMR (500 MHz, $CDCl_3$)

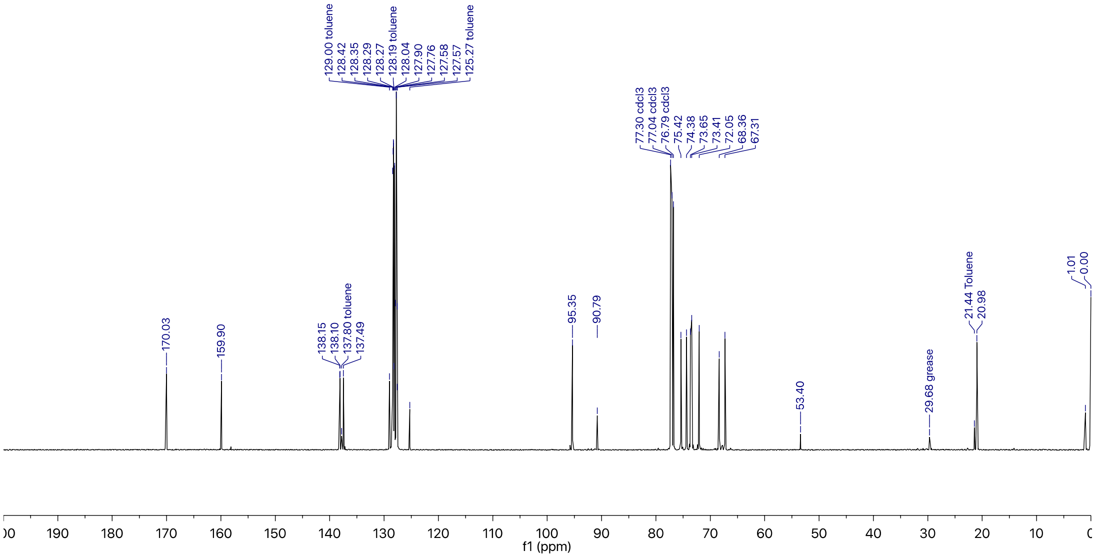

2,3,4,6-Tetra-*O*-acetyl-α-D-mannopyranosyl trichloroacetimidate (4)

^{1}H-NMR (500 MHz, $CDCl_3$)

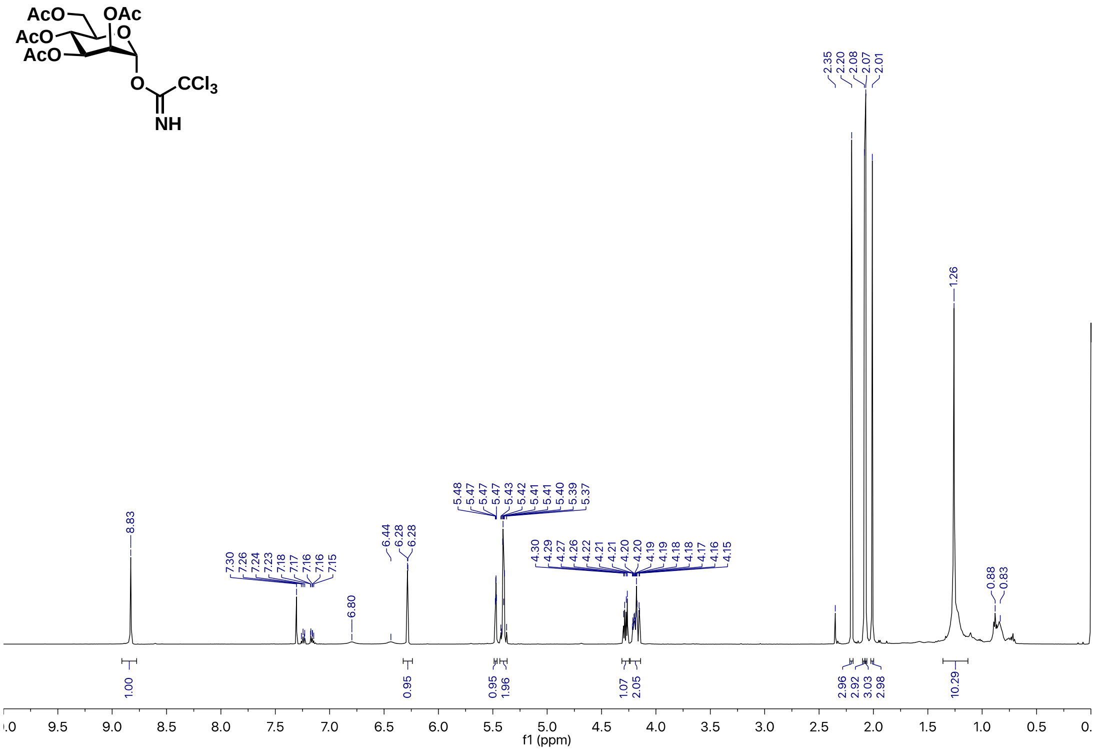

^{13}C-NMR (126 MHz, $CDCl_3$)

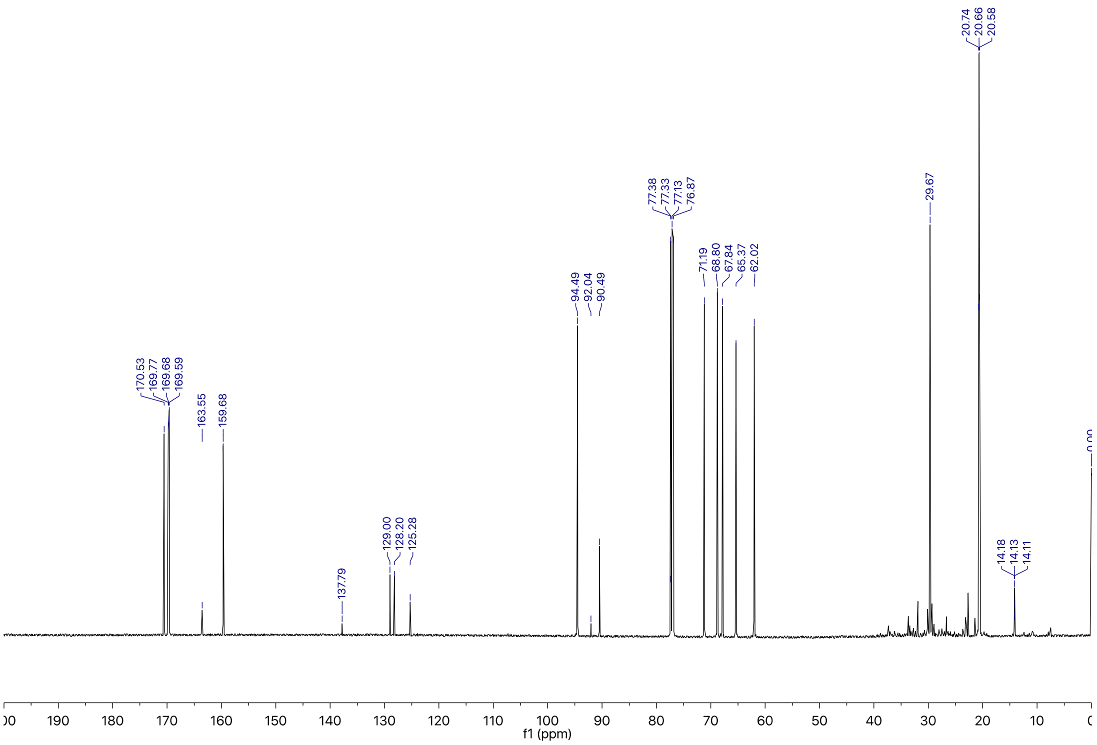

***N*-(Benzyl)-*N*-(4-(3,3,4,4,5,5,6,6,7,7,8,8,8-tridecafluorooctyl)benzyloxycarbonyl)-5-aminopent-1-yl 2-*O*-acetyl-3,4,6-tri-*O*-benzyl-α-D-mannopyranoside* (8)**

^{1}H-NMR (500 MHz, $CDCl_3$)

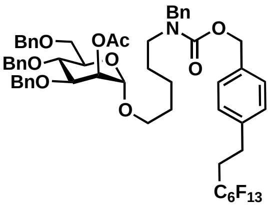

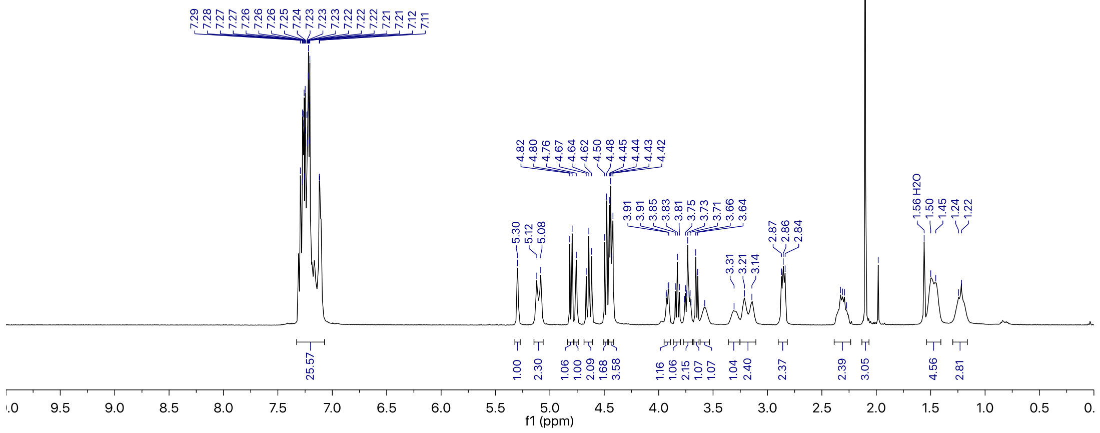

^{13}C-NMR (126 MHz, $CDCl_3$)

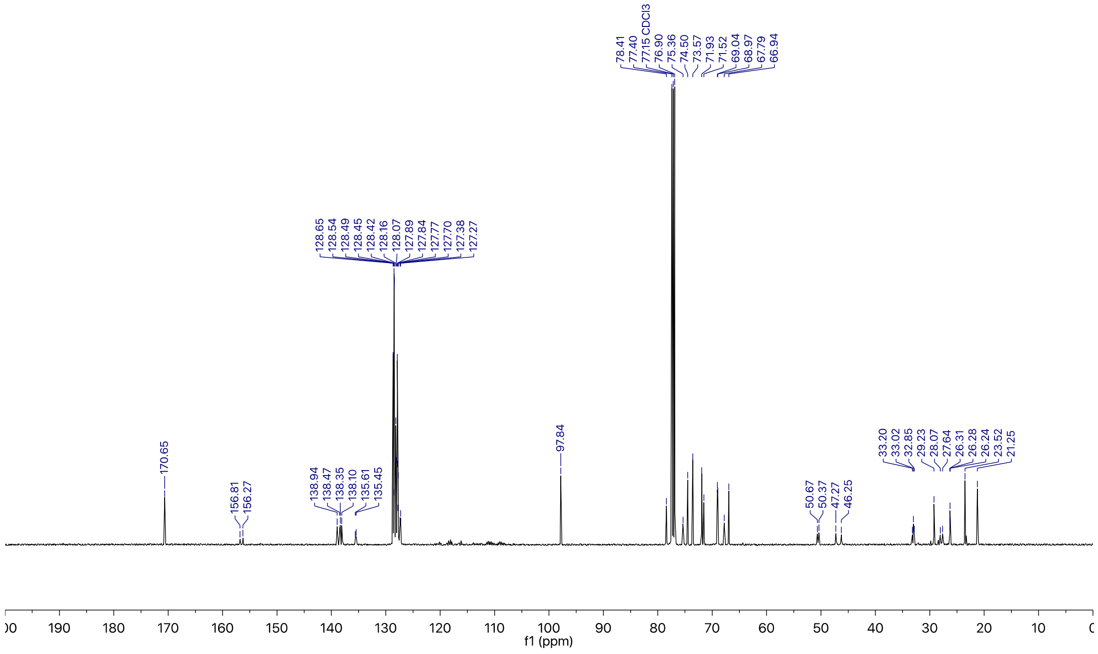

***N*-(Benzyl)-*N*-(4-(3,3,4,4,5,5,6,6,7,7,8,8,8-tridecafluorooctyl)benzyloxycarbonyl)-5-aminopent-1-yl 3,4,6-tri-*O*-benzyl-α-D-mannopyranoside* (9)**

^{1}H-NMR (500 MHz, $CDCl_3$)

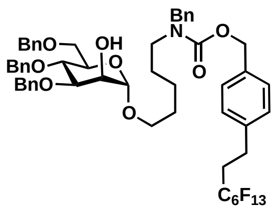

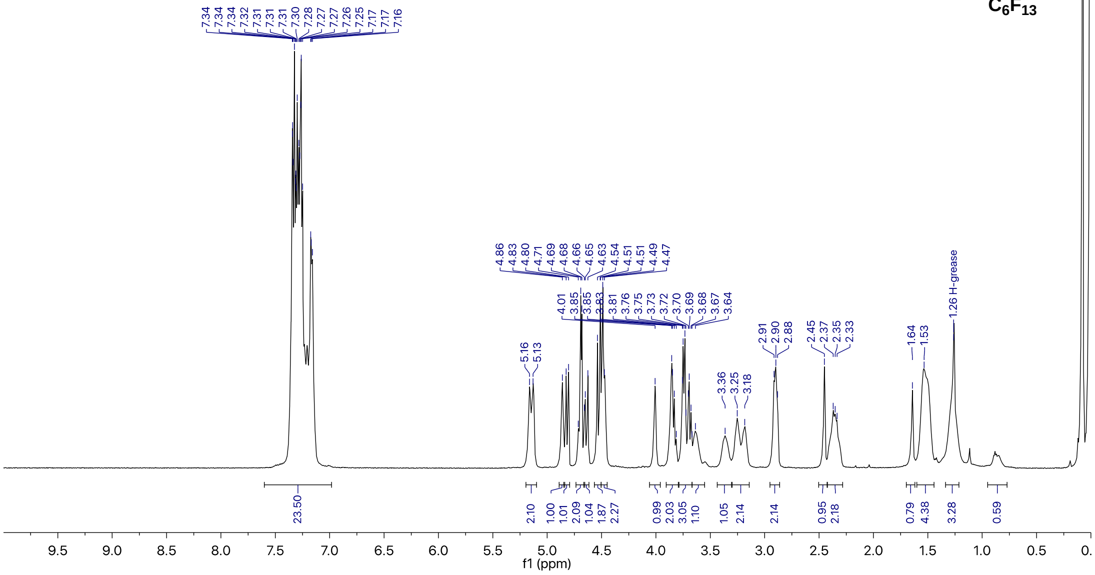

^{13}C-NMR (126 MHz, $CDCl_3$)

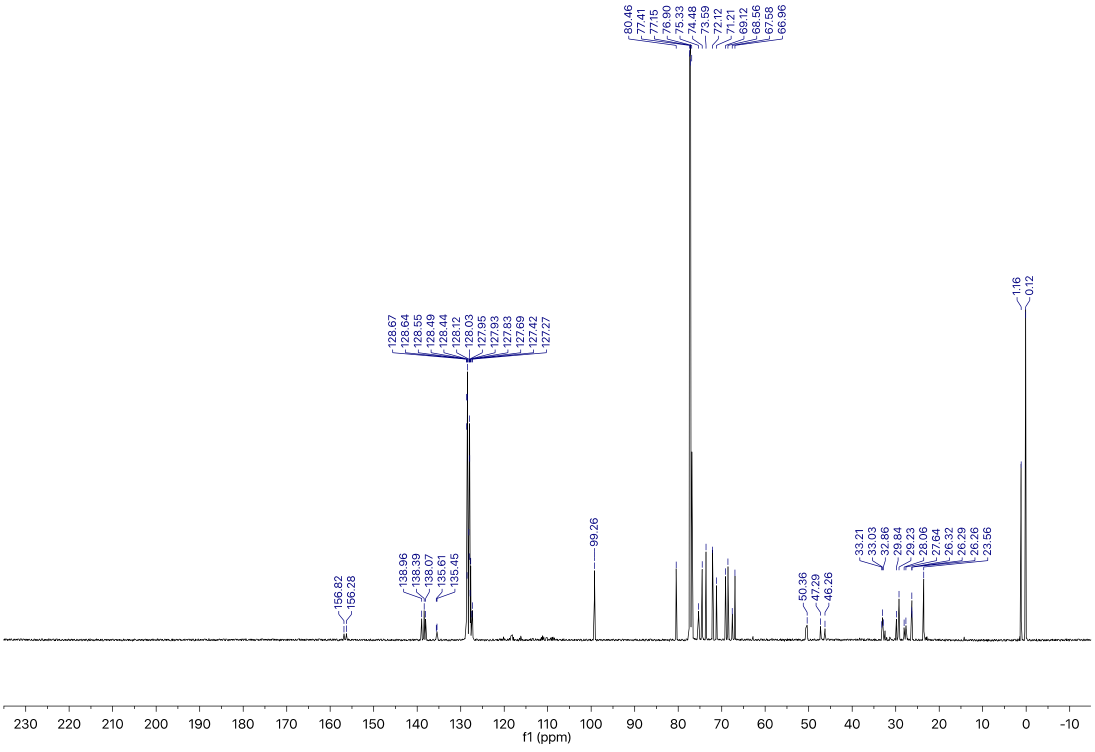

***N*-(Benzyl)-*N*-(4-(3,3,4,4,5,5,6,6,7,7,8,8,8-tridecafluorooctyl)benzyloxycarbonyl)-5-aminopent-1-yl (2-*O*-acyl-3,4,6-tri-*O*-benzyl-α-D-mannopyranosyl)-(1→2)-*O*-3,4,6-tri-*O*-benzyl-α-D-mannopyranoside* (10)**

^{1}H-NMR (500 MHz, $CDCl_3$)

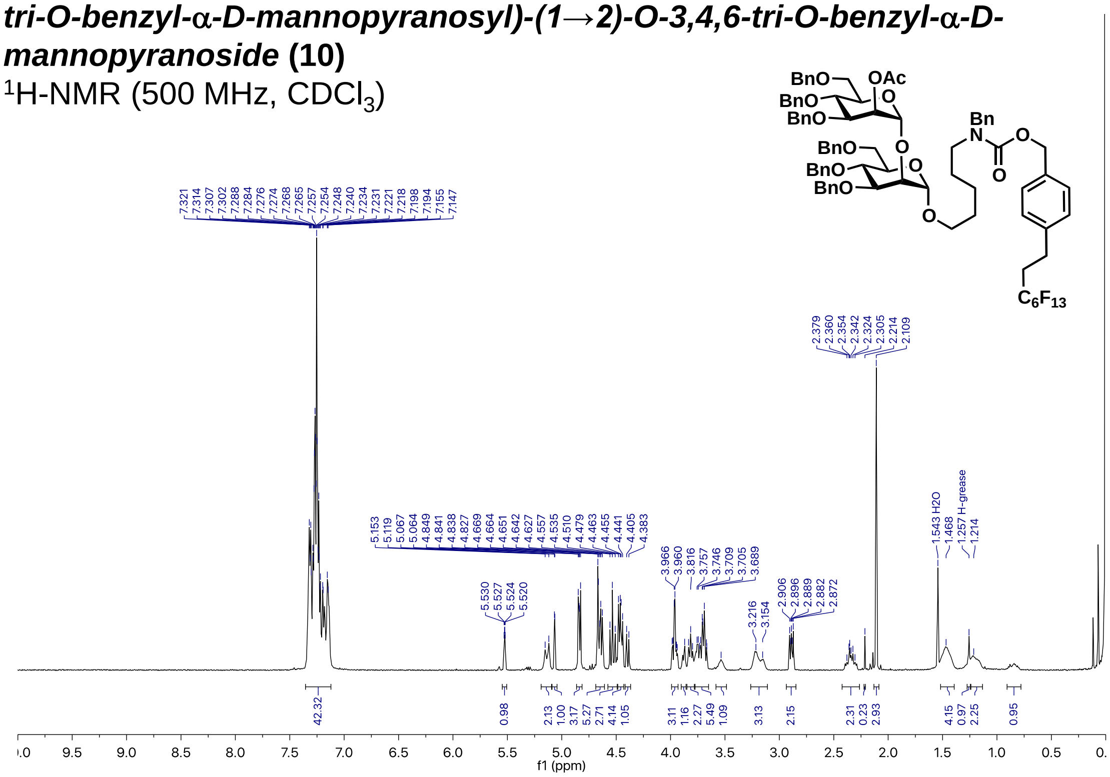

^{13}C-NMR (126 MHz, $CDCl_3$)

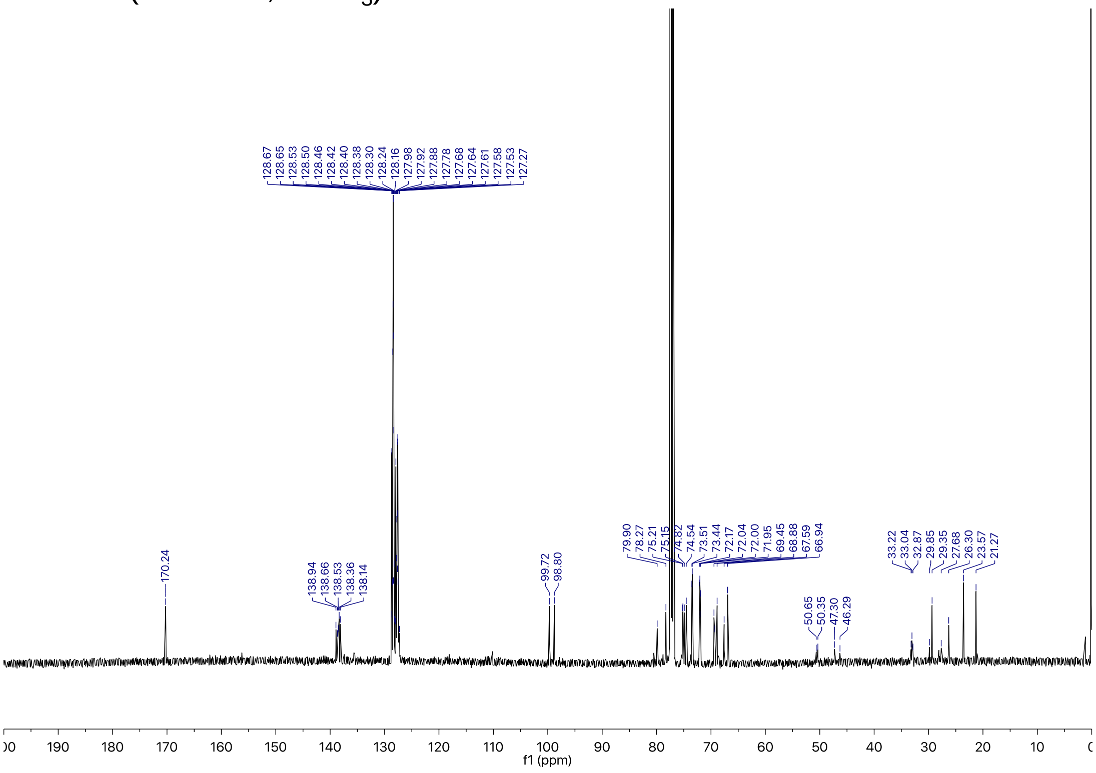

***N*-(Benzyl)-*N*-(4-(3,3,4,4,5,5,6,6,7,7,8,8,8-tridecafluorooctyl)benzyloxycarbonyl)-5-aminopent-1-yl (3,4,6-tri-*O*-benzyl-α-D-mannopyranosyl)-(1→2)-*O*-3,4,6-tri-*O*-benzyl-α-D-mannopyranoside* (11)**

^{1}H-NMR (500 MHz, $CDCl_3$)

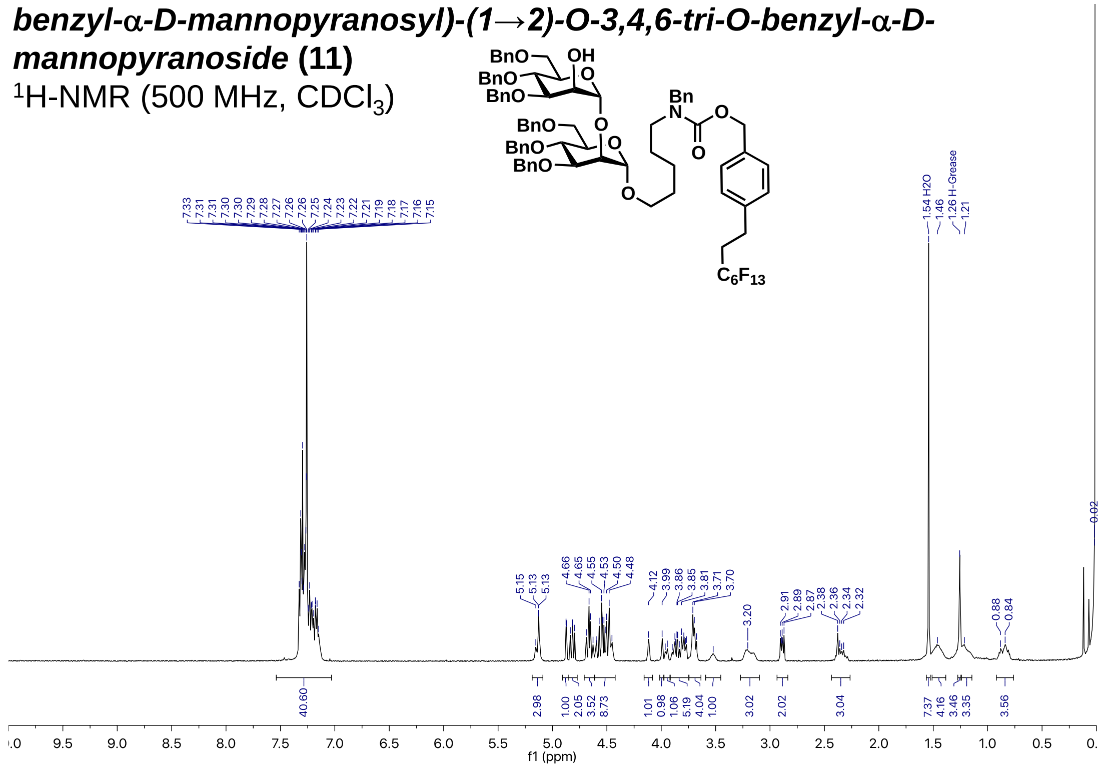

^{13}C-NMR (126 MHz, $CDCl_3$)

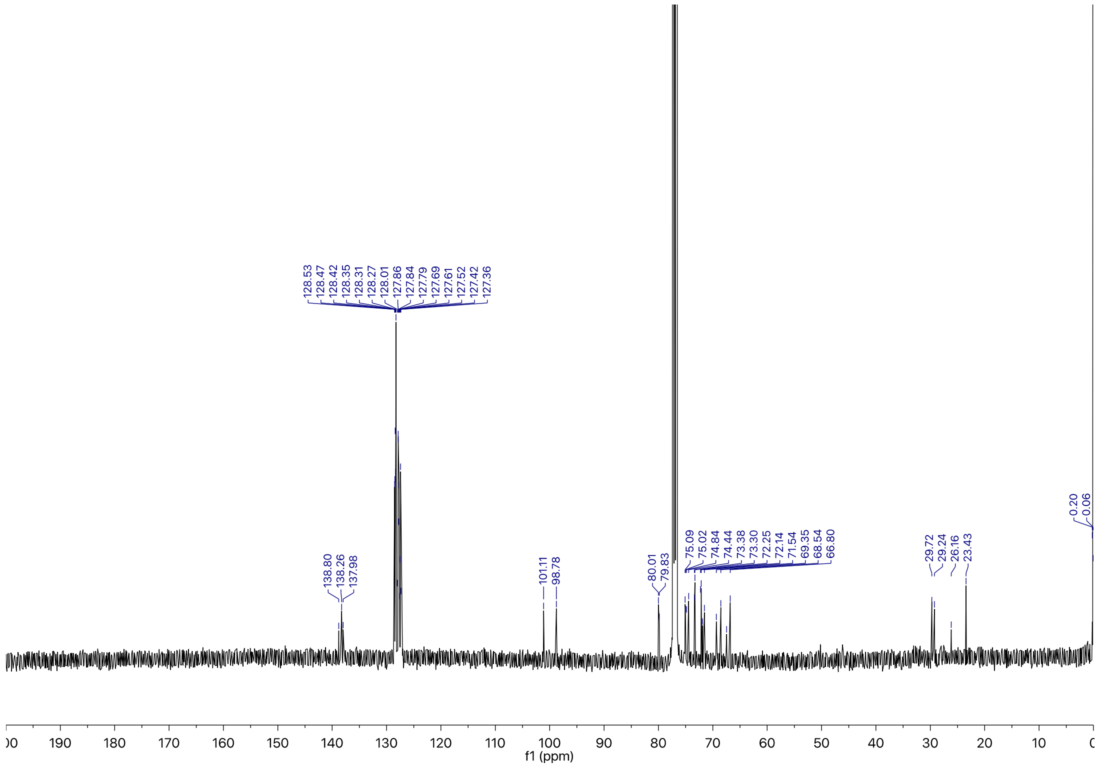

***N*-(Benzyl)-*N*-(4-(3,3,4,4,5,5,6,6,7,7,8,8,8-tridecafluorooctyl)benzyloxycarbonyl)-5-aminopent-1-yl* **[(2,3,4,6-tetra-*O*-acyl-α-D-mannopyranosyl)-(1→2)-*O*-(3,4,6-tri-*O*-benzyl-α-D-mannopyranosyl)]-(1→2)-*O*-3,4,6-tri-*O*-benzyl-α-D-mannopyranoside (12)**

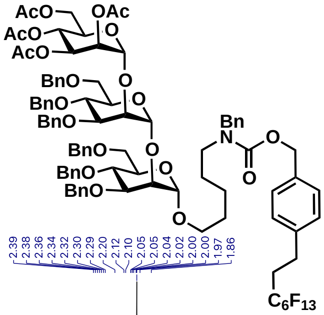

^{1}H-NMR (500 MHz, $CDCl_3$)

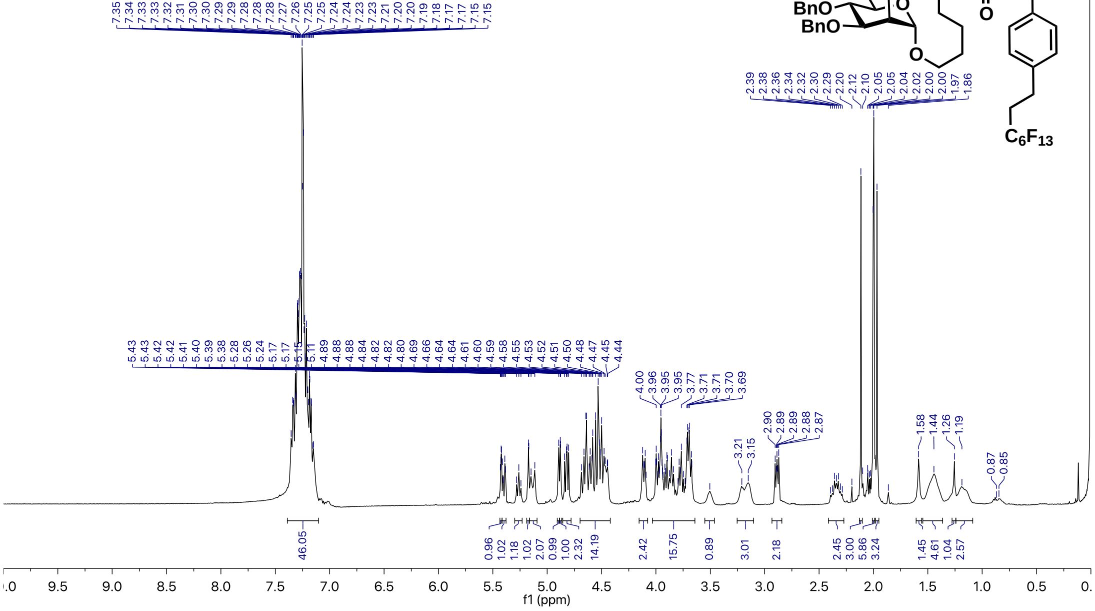

^{13}C-NMR (126 MHz, $CDCl_3$)

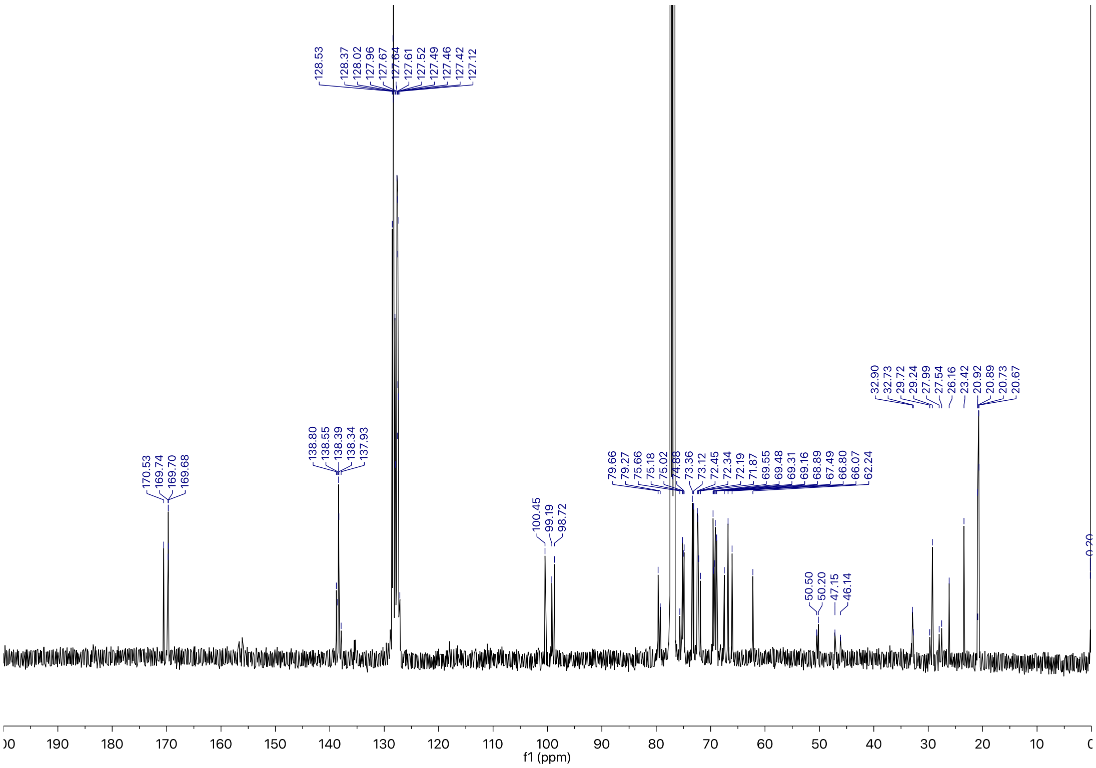

***N*-(Benzyl)-*N*-(4-(3,3,4,4,5,5,6,6,7,7,8,8,8-tridecafluorooctyl)benzyloxycarbonyl)-5-aminopent-1-yl* **[(2-*O*-acyl-3,4,6-tri-*O*-benzyl-α-D-mannopyranosyl)-(1→2)-*O*-(3,4,6-tri-*O*-benzyl-α-D-manno pyranosyl)]-(1→2)-*O*-3,4,6-tri-*O*-benzyl-α-D-mannopyranoside (13)**

^{1}H-NMR (500 MHz, $CDCl_3$)

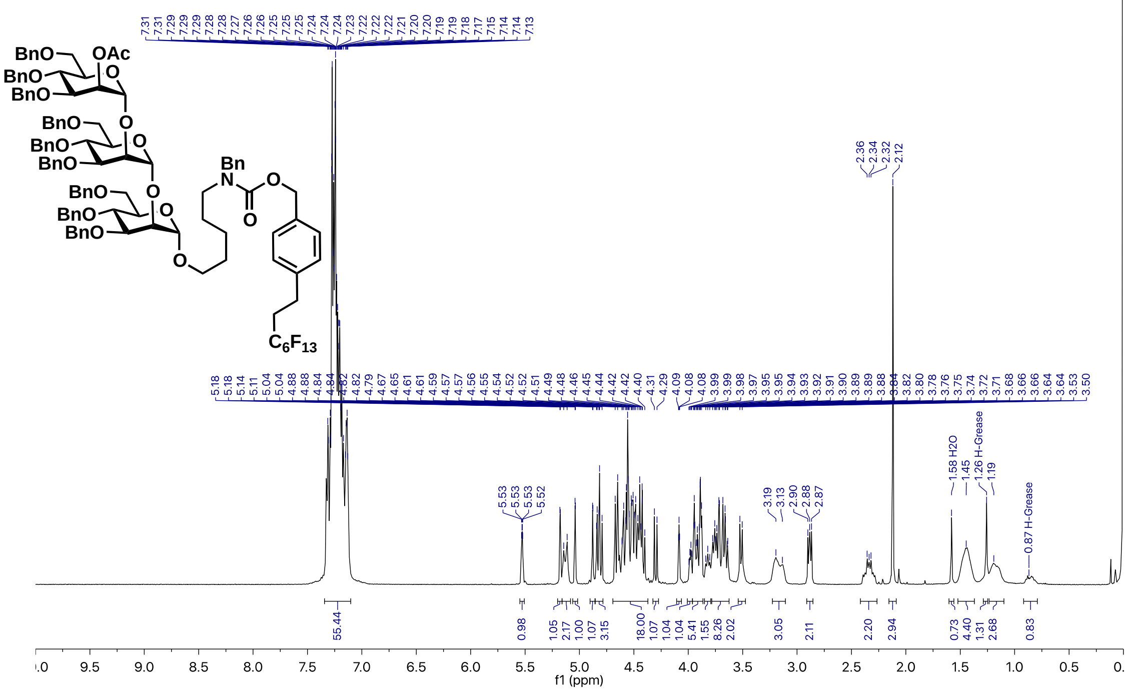

^{13}C-NMR (126 MHz, $CDCl_3$)

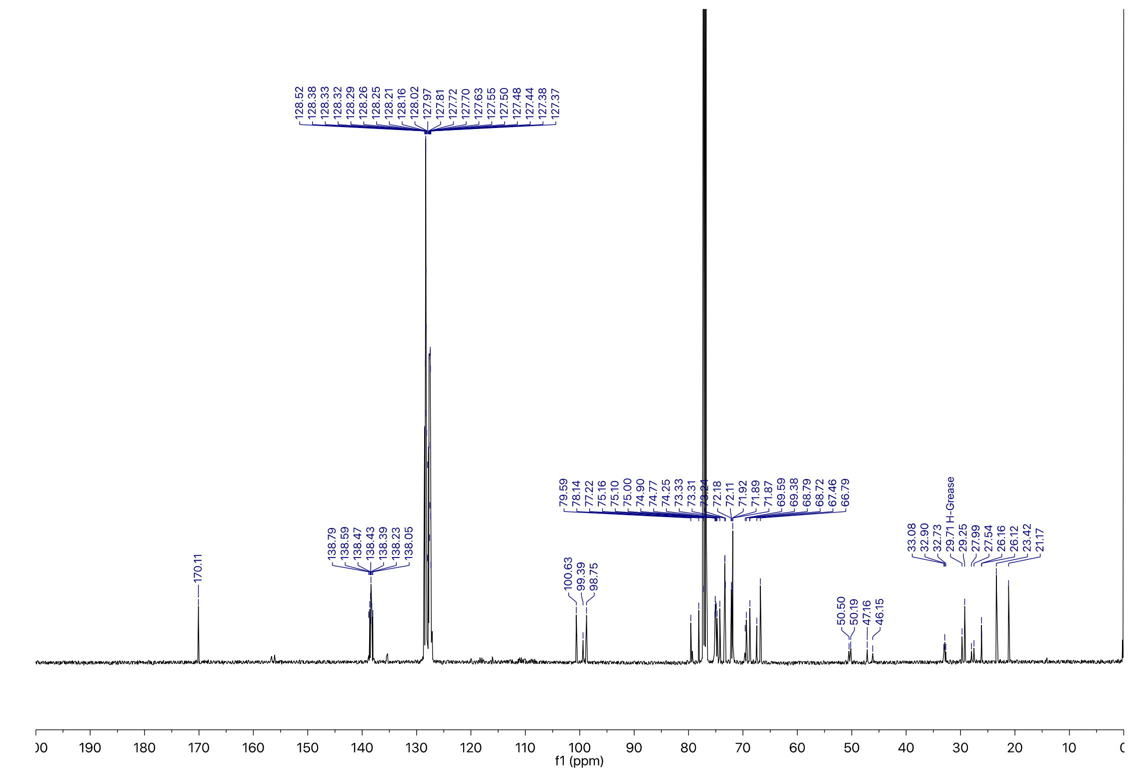

***N*-(Benzyl)-*N*-(4-(3,3,4,4,5,5,6,6,7,7,8,8,8-tridecafluorooctyl)benzyloxycarbonyl)-5-aminopent-1-yl* **[(3,4,6-tri-*O*-benzyl-α-D-mannopyranosyl)-(1→2)-*O*-(3,4,6-tri-*O*-benzyl-α-D-mannopyranosyl)]-(1→2)-*O*-3,4,6-tri-*O*-benzyl-α-D-mannopyranoside (15)**

^{1}H-NMR (500 MHz, $CDCl_3$)

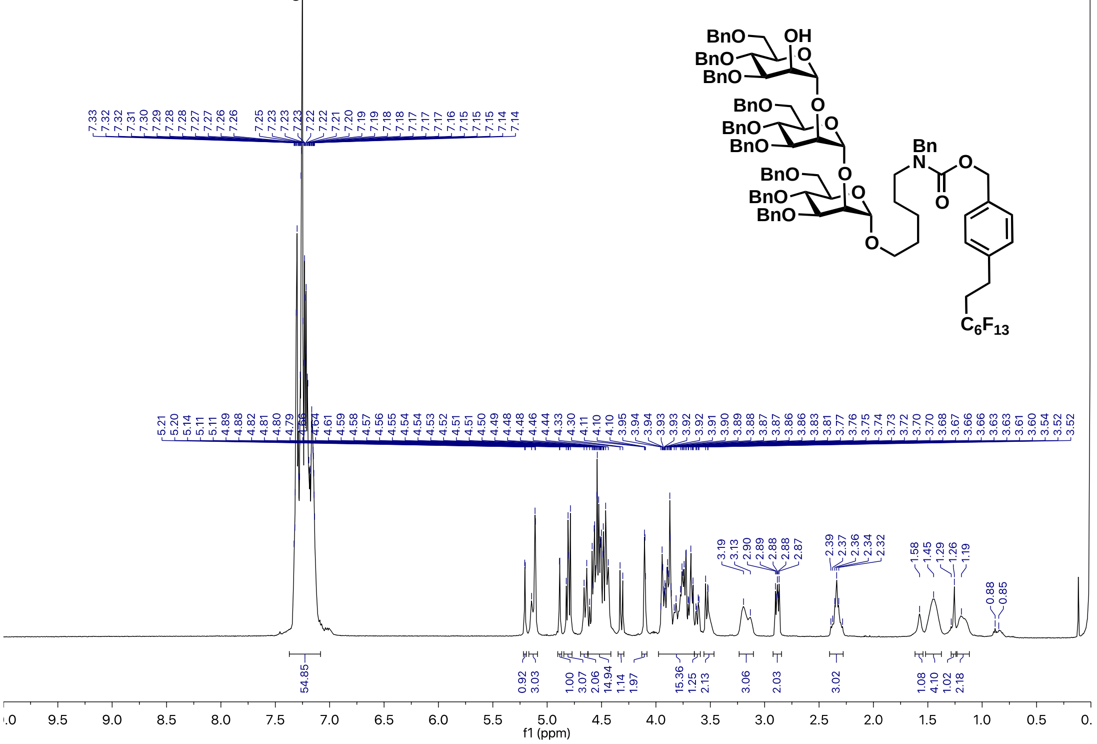

^{13}C-NMR (126 MHz, $CDCl_3$)

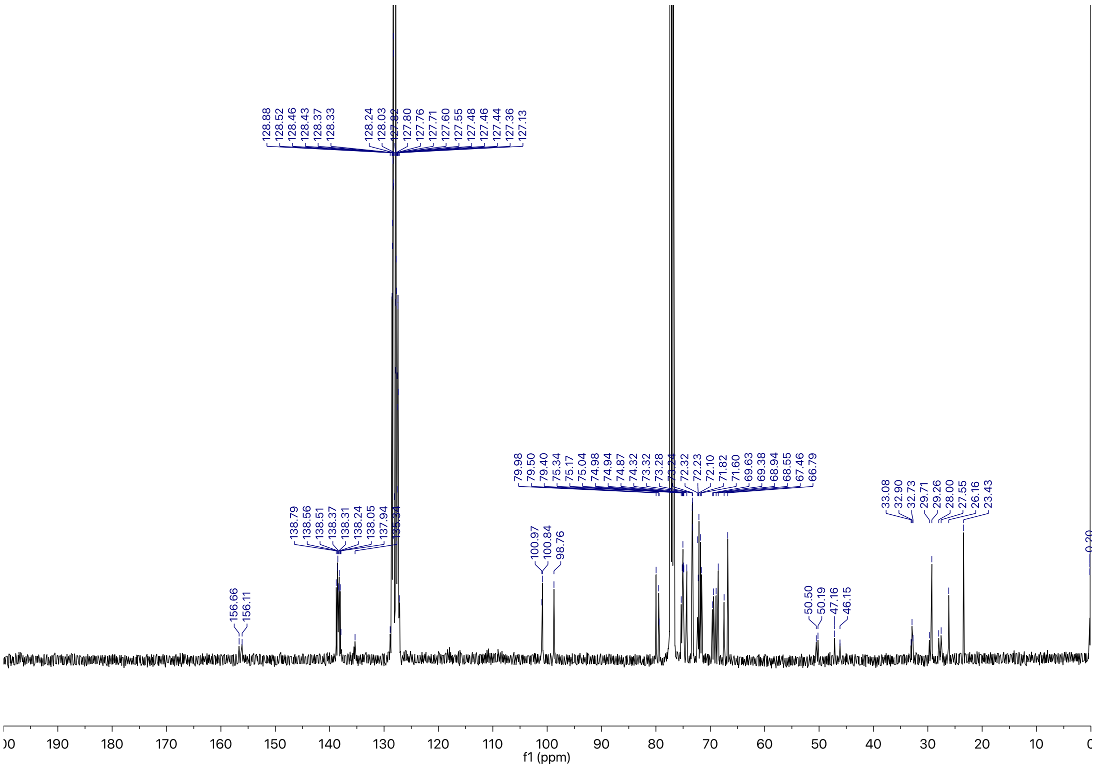

5-Aminopentyl α-D-mannopyranosyl-(1→2)-α-D-mannopyranosyl-(1→2)-α-D-mannopyranoside (16)

^{1}H-NMR (500 MHz, D_2O)

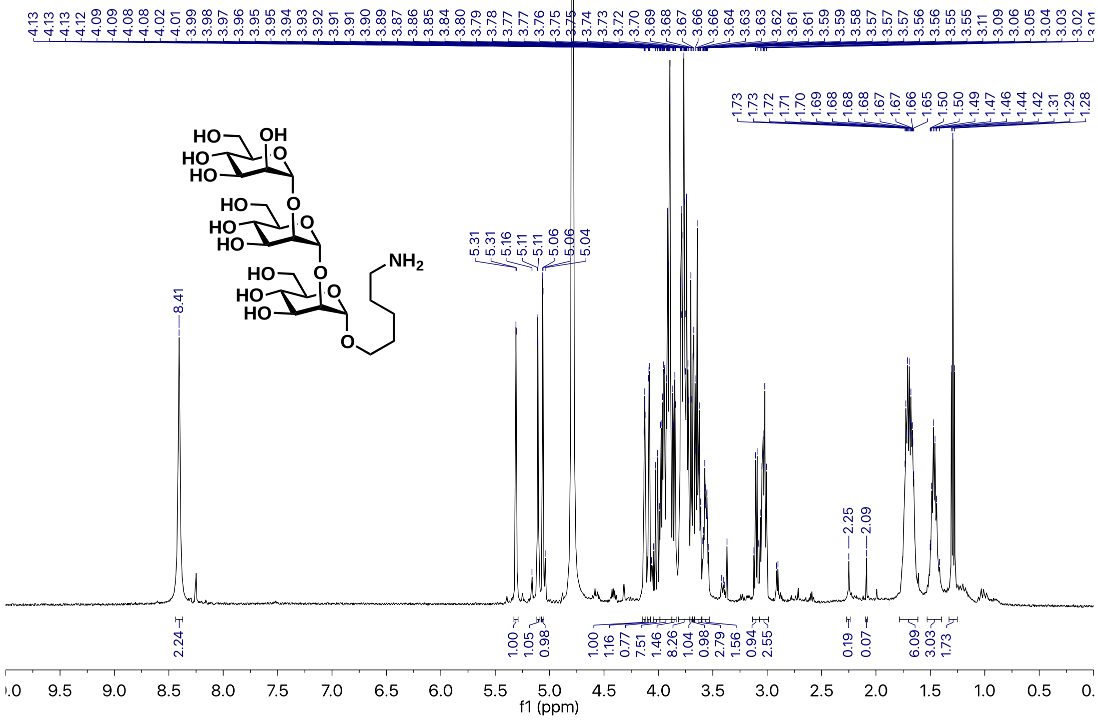

^{13}C-NMR (126 MHz, D_2O)

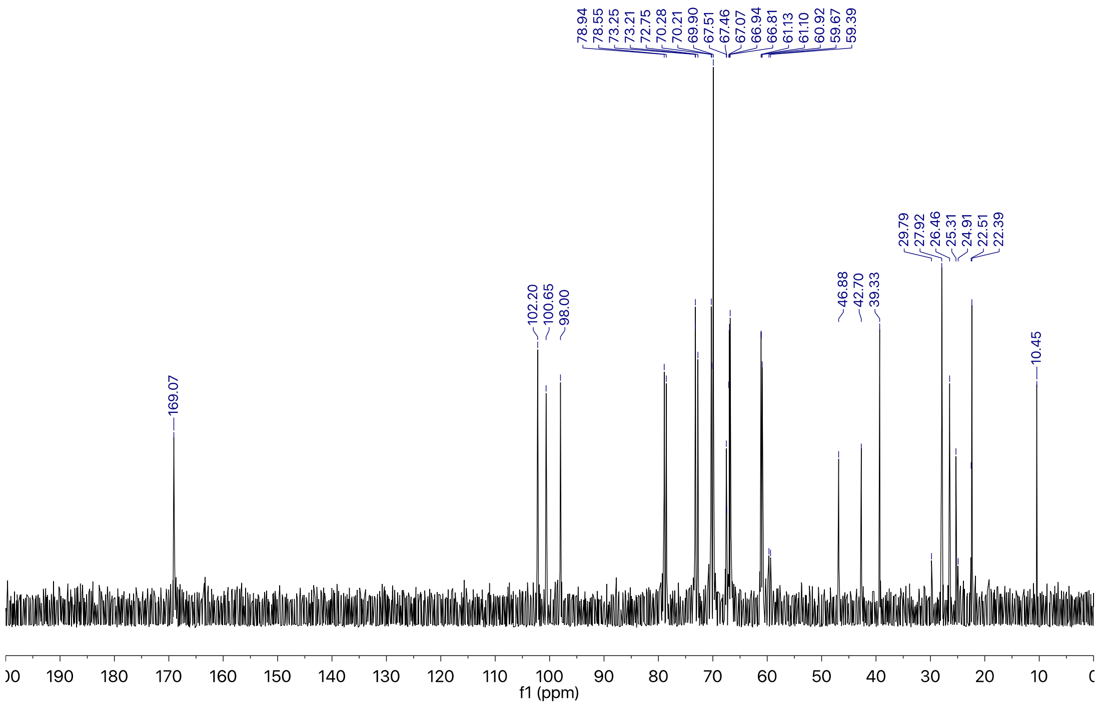

Di-*tert*-butyl (*S*)-4-(2-((((9*H*-fluoren-9-yl)methoxy)carbonyl)amino)-6-((diphenyl (*p*-tolyl)methyl) amino)hexanamido)-4-(3-(*tert*-butoxy)-3-oxopropyl)heptanedioate (19)

^{1}H-NMR (500 MHz, $CDCl_3$)

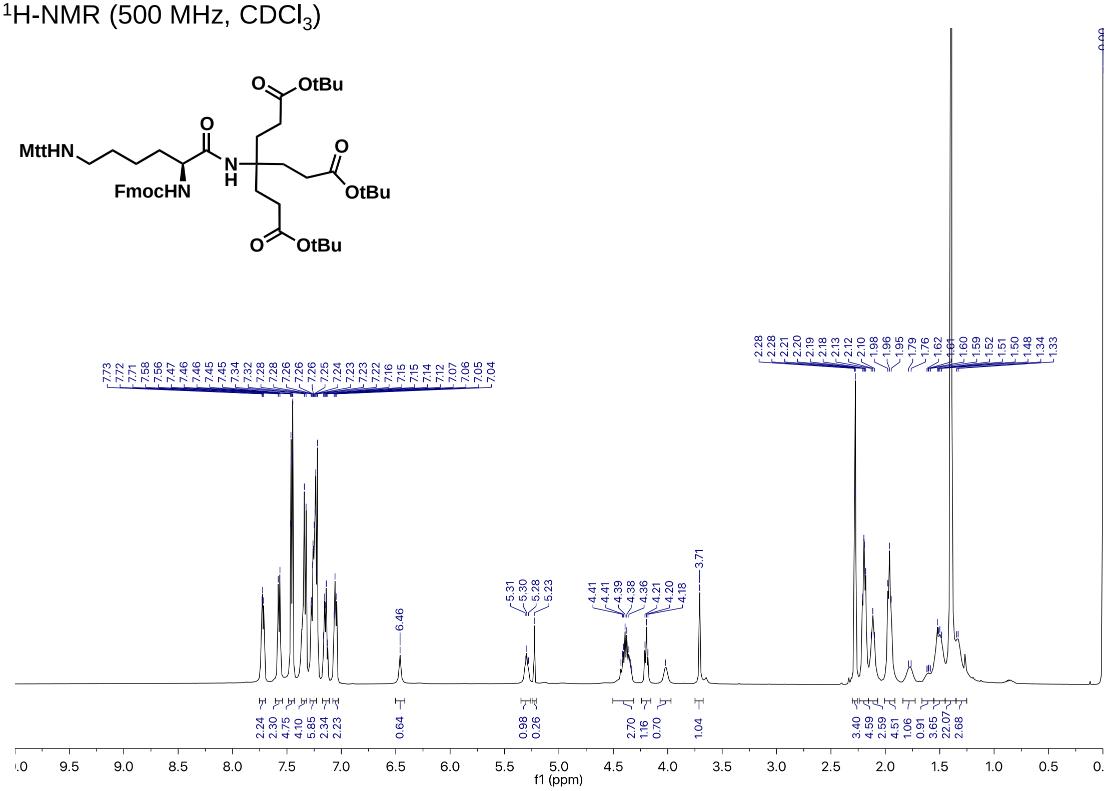

^{13}C-NMR (126 MHz, $CDCl_3$)

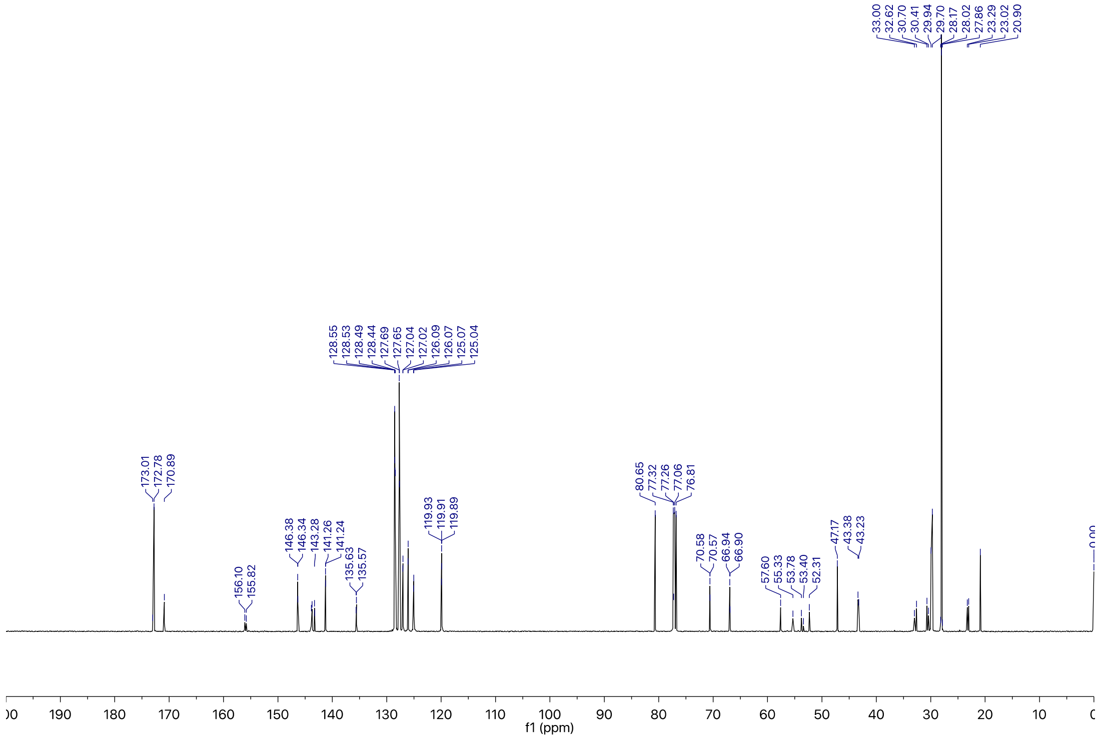

Di-*tert*-butyl (*S*)-4-(2-(6-((((9*H*-fluoren-9-yl)methoxy)carbonyl)amino) hexanamido)-6-((diphenyl (*p*-tolyl)methyl)amino)hexanamido)-4-(3-(*tert*-butoxy)-3-oxopropyl)heptanedioate (22)

^{1}H-NMR (500 MHz, CD_3CN)

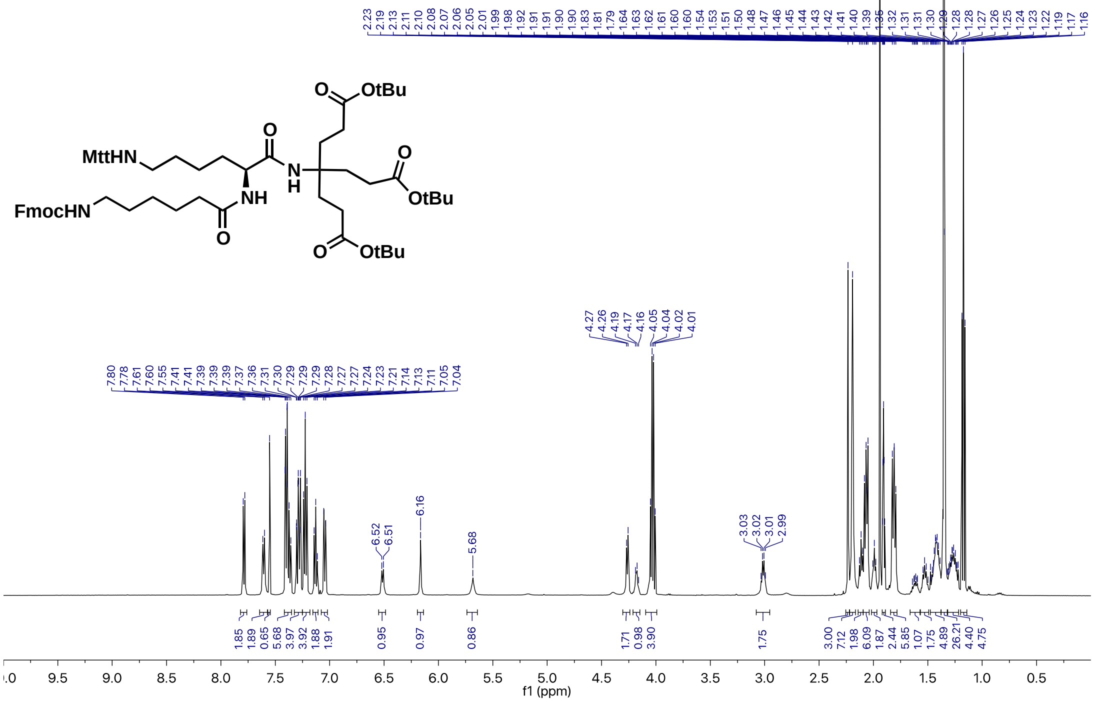

^{13}C-NMR (126 MHz, CD_3CN)

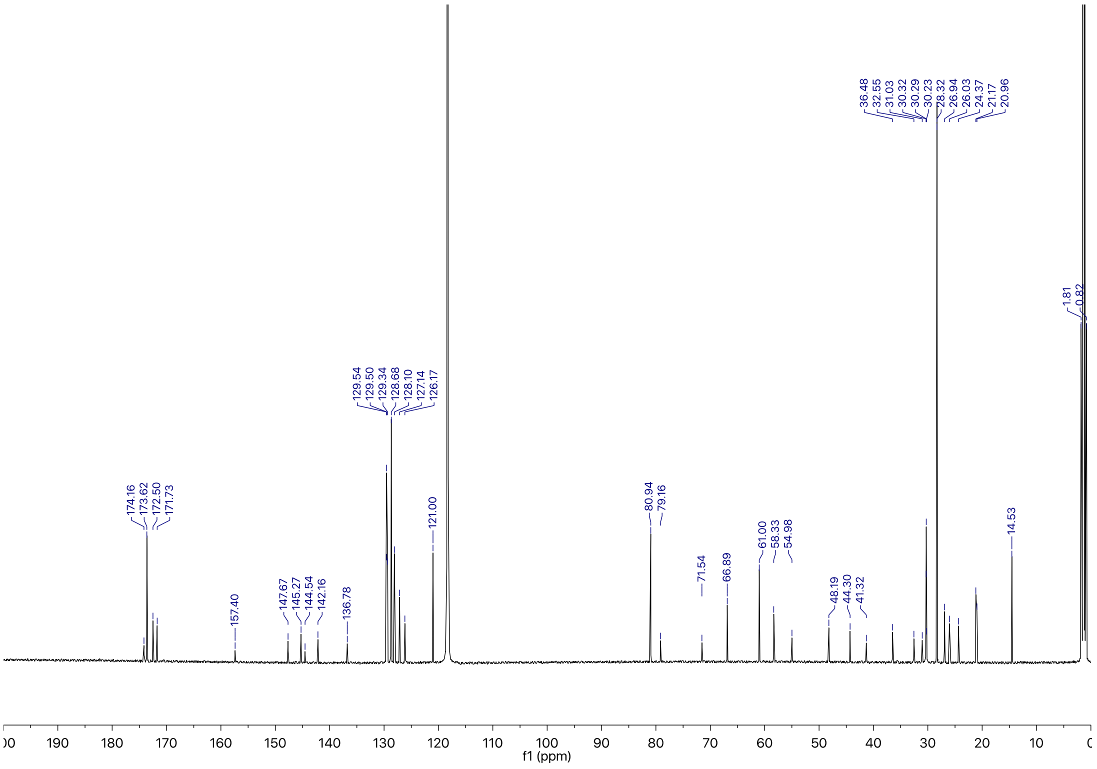

(*S*)-5-((5-(6-((((9*H*-Fluoren-9-yl)methoxy)carbonyl)amino)hexanamido)-6-((1,7-di-*tert*-butoxy-4-(3-(*tert*-butoxy)-3-oxopropyl)-1,7-dioxoheptan-4-yl)amino)-6-oxohexyl)carbamoyl)-2-(6-(dimethylamino)-3-(dimethyliminio)-3*H*-xanthen-9-yl)benzoate (24)

^{1}H-NMR (500 MHz, CD_3CN)

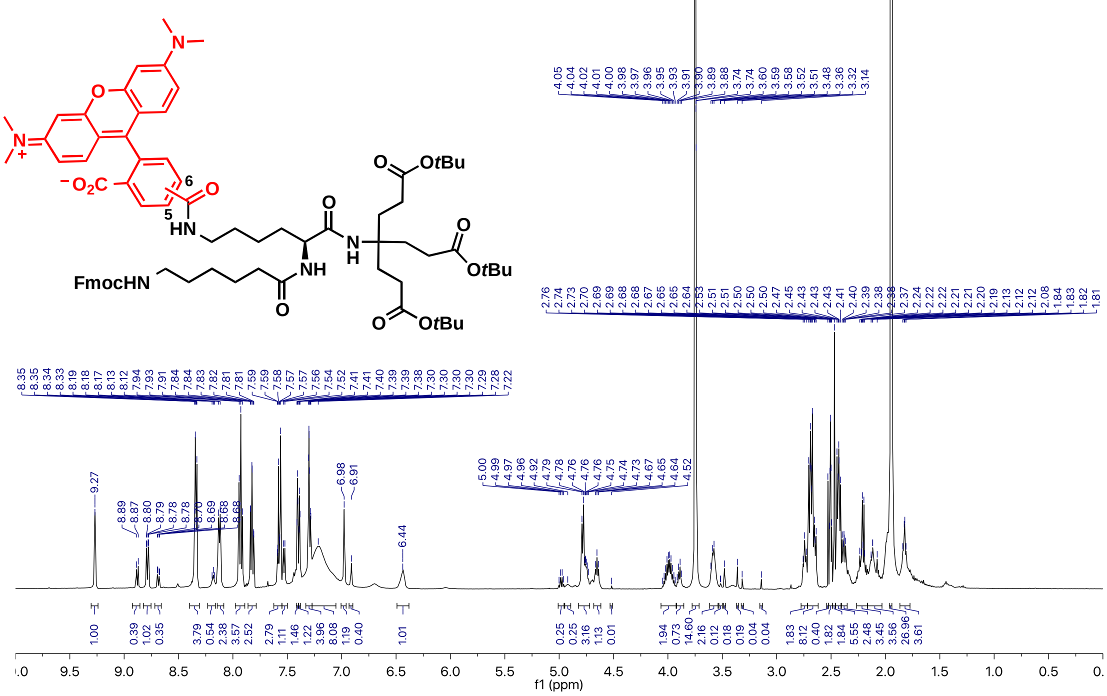

^{13}C-NMR (126 MHz, CD_3CN)

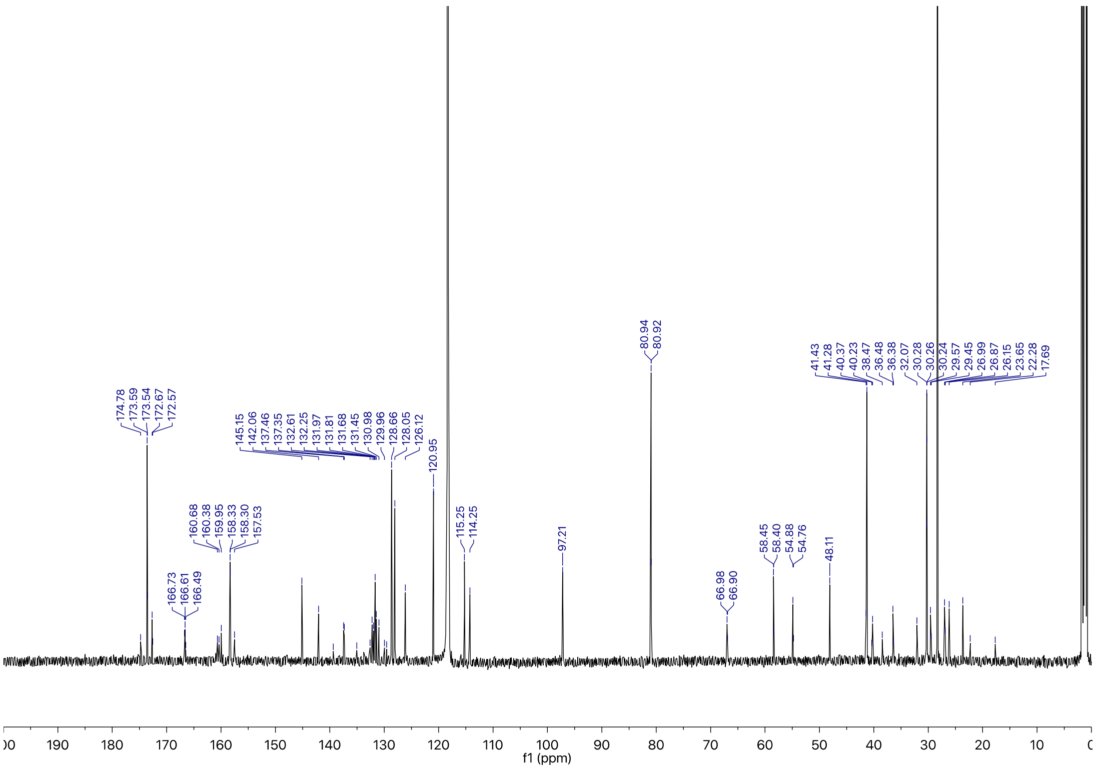

Compound (27) ^{1}H-NMR (600 MHz, D_2O)

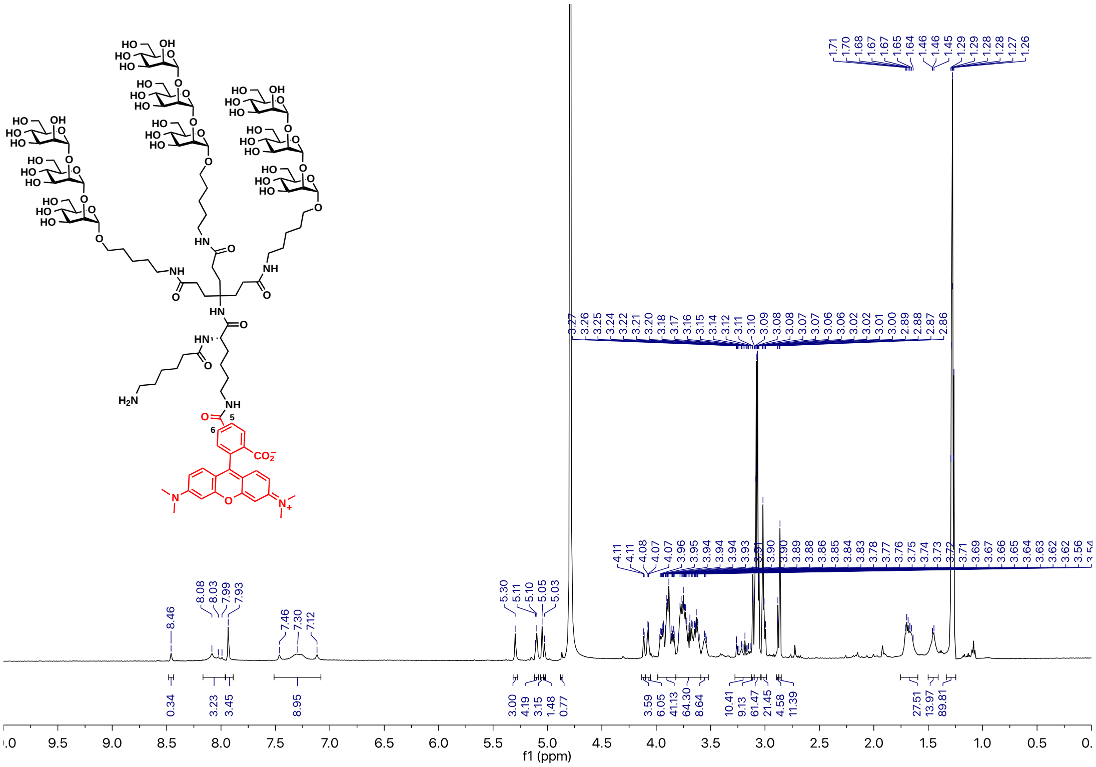

LCMS data (27)

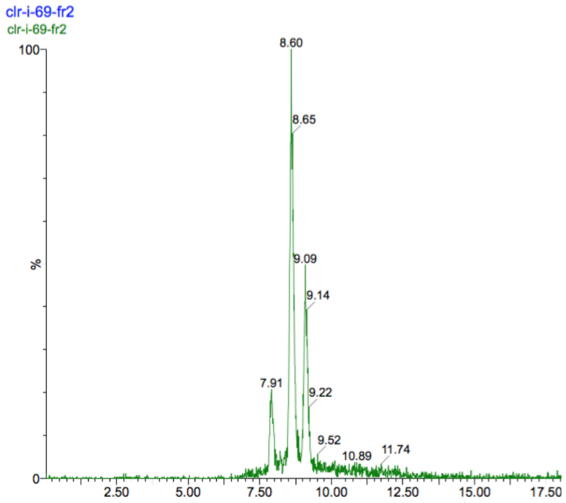

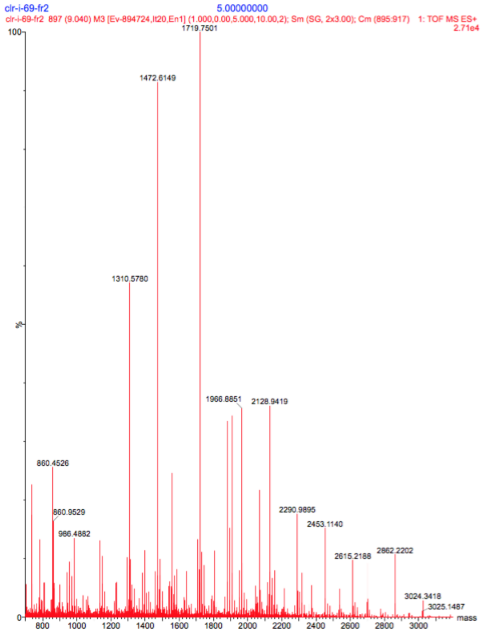

Supplement: File 1 — Experimental, characterization data and copies of spectra. [file Beilstein_J_Org_Chem-15-623-s001.pdf]
